# Advancing AI-driven thematic analysis in qualitative research: a comparative study of nine generative models on Cutaneous Leishmaniasis data

**DOI:** 10.1186/s12911-025-02961-5

**Published:** 2025-03-10

**Authors:** Issam Bennis, Safwane Mouwafaq

**Affiliations:** Mohammed VI International School of Public Health, Mohammed VI University of Sciences and Health, Casablanca, Morocco

**Keywords:** Artificial intelligence in qualitative research, Large language models, Thematic analysis, Grounded theory development, Natural language processing, Research automation, Cutaneous leishmaniasis

## Abstract

**Background:**

As part of qualitative research, the thematic analysis is time-consuming and technical. The rise of generative artificial intelligence (A.I.), especially large language models, has brought hope in enhancing and partly automating thematic analysis.

**Methods:**

The study assessed the relative efficacy of conventional against AI-assisted thematic analysis when investigating the psychosocial impact of cutaneous leishmaniasis (CL) scars. Four hundred forty-eight participant responses from a core study were analysed comparing nine A.I. generative models: Llama 3.1 405B, Claude 3.5 Sonnet, NotebookLM, Gemini 1.5 Advanced Ultra, ChatGPT o1-Pro, ChatGPT o1, GrokV2, DeepSeekV3, Gemini 2.0 Advanced with manual expert analysis. Jamovi software maintained methodological rigour through Cohen’s Kappa coefficient calculations for concordance assessment and similarity measurement via Python using Jaccard index computations.

**Results:**

Advanced A.I. models showed impressive congruence with reference standards; some even had perfect concordance (Jaccard index = 1.00). Gender-specific analyses demonstrated consistent performance across subgroups, allowing a nuanced understanding of psychosocial consequences. The grounded theory process developed the framework for the fragile circle of vulnerabilities that incorporated new insights into CL-related psychosocial complexity while establishing novel dimensions.

**Conclusions:**

This study shows how A.I. can be incorporated in qualitative research methodology, particularly in complex psychosocial analysis. Consequently, the A.I. deep learning models proved to be highly efficient and accurate. These findings imply that the future directions for qualitative research methodology should focus on maintaining analytical rigour through the utilisation of technology using a combination of A.I. capabilities and human expertise following standardised future checklist of reporting full process transparency.

**Supplementary Information:**

The online version contains supplementary material available at 10.1186/s12911-025-02961-5.

## Background

Thematic analysis is a cornerstone of qualitative research methodology and is quite variable from researcher to researcher due to reliance on sophisticated human reasoning and interpretative skills [1, 2]. These structured approaches may enable strong comparative analyses with established literature. A conceptual exploration within methodological frameworks requires strict logical processes and systematic data classification to recognise and articulate patterns and their subsidiary components [3]. Thematic analysis is appropriate when analysing extensive text-based material and when researchers want to reflect on people’s experiences, thoughts, and behaviours [3]. Indeed, the depth of complexity found in qualitative data requires significant mental pre-work and ongoing engagement throughout the analysis from researchers [4]. Contemporary qualitative analysis has evolved along two parallel but intertwined paths. The first trajectory includes analysing atypical data and allows researchers to discover latent logical patterns and possible correlations [5]. This form woven multi-disciplinary efforts based on inductive and abductive inference of contemporary ground theory [6, 7]​​. Such process approaches produce and test hypotheses based on new or novel observations beyond the original themes or patterns. The second trajectory is technological, concerned with Computer-Assisted Qualitative Data Analysis Software (CAQDAS), which allows researchers to quickly triangulate qualitative with quantitative approaches while working on datasets of considerable size. CAQDAS has saved 20 − 30% of the time in enabling the management of data storage, manipulation and retrieval processes [4, 8]. Generative artificial intelligence (A.I.) has sparked exciting advancements in qualitative data analysis in scientific fields [9, 10]. A recently developed prompt, now popular approach to analysing an enormous amount of textual data, is using large language models (LLMs such as ChatGPT) [11]. LLM can be trained on a vast corpus of text that is perfect for making Natural Language Processing (NLP) a headline-making technology and subsequently generating relevant keywords, patterns, and links at the level of micro semantics very quickly and efficiently [12].

There are several examples where A.I. is applicable to enhance the holistic components of qualitative analysis by automating the steps of qualitative research that most researchers consider tedious or repetitive, including transcription, translation and initial coding texts [11]. Automating these manual workflows turbocharges result generation, allows focus more on interpretative analytics and aids with potential bias [9]. They also indicate another potential advantage in that the analytical algorithms that A.I. use can be analysed by behavioural thresholds unattainable by humans so that more nuanced analyses beyond the scope (which humans may miss or overlook) are possible to run [13]. In addition, A.I. text can serve as a valuable comparator for research interpretation, potentially uncovering biases and expanding interpretative frameworks [1, 2, 11]. ChatGPT and other A.I. models can articulate their results, offering researchers valuable context. Moreover, with this transparency, the reproduction of the results can be assured with lower potential human subjectivity bias [14–16]. Some artificial intelligence models reorganise information based on questions, which improves data structuring and analysis [11, 17]. Alternatively, if data is uncertain or there are programming errors or inaccuracies in the data or inputs, one may distrust the results [11].

Furthermore, qualitative research requires immersive interpretation, acceptance of unusual reflections, and flexibility paradigms from the researcher, considered part of the analysis process, making it incredibly misunderstood for A.I. algorithms to prompt [2, 18]. Therefore, caution must be taken while using A.I. and interpreting A.I. based results [18, 19]. Hence, researchers need to check and verify their ongoing results by doing strict quality control procedures, including rigorous appraisal and validation of research outputs [12, 13, 17]. In this context, this study seeks to assess whether ChatGPT o1-Pro and a diverse set of eight other generative A.I. models can improve the accuracy of qualitative synthesis in complex evidence concerning the psychosocial burden of cutaneous leishmaniasis scarring when compared to traditional human-led qualitative analysis approaches.

## Materials

### Study design

This comparative study was conducted to evaluate the feasibility of use of artificial intelligence to inform social science inquiry in practice, here realised through thematic analysis versus human-led qualitative analysis. The central comparative question posed was whether contemporary generative A. I. models and their updated versions can offer advantages of accuracy, efficiency, and insightful perspectives as much or over traditional qualitative methods.

### Participants

This study used data from a preliminary study on cutaneous leishmaniasis psychological effects performed on Moroccan high school students (Bennis et al., 2017) [20]. This dataset was selected because it was included in the findings of a systematic review published in August 2024, which found that it was an important source for exploring the psychosocial dimensions of cutaneous leishmaniasis among male and female students [21]. This dataset consisted of 448 direct quotations extracted directly from the primary study’s student responses, enabling direct comparison of the two methodological approaches [20].

The first approach employed traditional qualitative analysis in two stages, the second author, a Professor of Public Health, with a qualitative background and more than ten years of experience in the field. The second approach done by the first author using nine generative AI models. The first author had an experience with qualitative research, including with a number of QACDAS qualitative analysis software packages.

July 2024 and December 2024 were two time slots for choosing the different A.I. models. The selected models reflect the latest in deep learning for language generation and was promoted as applicating better natural language-processing algorithms. Models from the July cohort included Llama 3.1 405B, Claude 3.5 Sonnet, NotebookLM, Gemini 1.5 Advanced Ultra and ChatGPT o1-preview models. While from the December cohort included ChatGPT o1 that replaced the preview one, GrokV2, DeepSeekV3, and Gemini 2.0 Advanced. The 9th model that was added was in December 2024 a recently released very advanced commercial model ChatGPT o1-Pro.

The results from both approaches were compared with reference findings (Named **Reference A)** corresponding to the human decision with Nvivo software, as shown in Suplementary material 1. These reference A findings were issued from a multi-disciplinary analysis by a multi-national team of anthropologists, sociologists, professors and specialists in veterinary and human public health built earlier by Bennis et al., 2017 [20].

### Study location

The study was conducted in a regulated academic environment to minimise the influence of external factors and ensure the accuracy of the results. All analyses employing manual (Man_1 & Man_2 done by the second researcher) or computer-assisted analysis done by the first researcher in two periods.

### Description of instruments used

A.I models are chosen based on the reputation of developers among artificial intelligence experts and some latest use of 2024 updates. Llama 3.1 405B from Meta A.I. (formerly Facebook A.I. Research) was initially taken. This model is optimised for NLP formative tasks, has high integrity processing understanding abilities, and performs accurately in textual data [22]. **Claude 3.5 Sonnet** by Anthropic [23]. Both can produce contextually based text, which renders them able candidates for complex qualitative analysis studies.

A language model powered by machine and deep learning and developed at Google Research known as **Notebook LM**, scientists-interactive-exploration allows for analysis and synthesis of large text corpora [24]. The DeepMind **Gemini 1.5 Advanced Ultra** is a NLP model that supports more intricate analysis and exact synthesis as marketed [25]. This makes both tools suitable to analyse academic or professional content since they have been designed with architectures for synthesising large volumes of data. In December, other updated and new models were introduced. **Gemini 2.0 Advanced** is the model that improves capabilities in complex tasks like programming, mathematics, logic, and teaching [26]. **GrokV2** is X’s A.I. chatbot model solution ended up building directly into the X platform (Former Twitter) [27]. **DeepSeekV3** is famous for its large open-source language model with a mixture of expert architecture fully free of charge [28]. **ChatGPT o1** is the new version of GPT4 (Generative Pre-trained Transformer). This natural language processing model replaced in December the o1-preview functionality. It is presented with the particularity to spend more time reasoning before understanding the task structure and solving it more effectively [29]. Lastly, **ChatGPT o1-Pro**, a model produced by OpenAI that costs 200 dollars per month, is the most useful for professional tasks including academic research and analysis that need consistent, high-quality A.I. results across multiple requests interactions, understanding, and reasoning [30].

### Data collection and preparation procedures

The quotes were written by 454 students who noticed six refusals to participate in the main study (Bennis et al. 2017). Therefore, 448 quotes were collected and anonymised as PDF files available as supplementary materials at this link [31] and Supplementary material 2. Every quote is a separate response unit for this current thematic analysis.

### Data analysis process

**Fig. 1:**
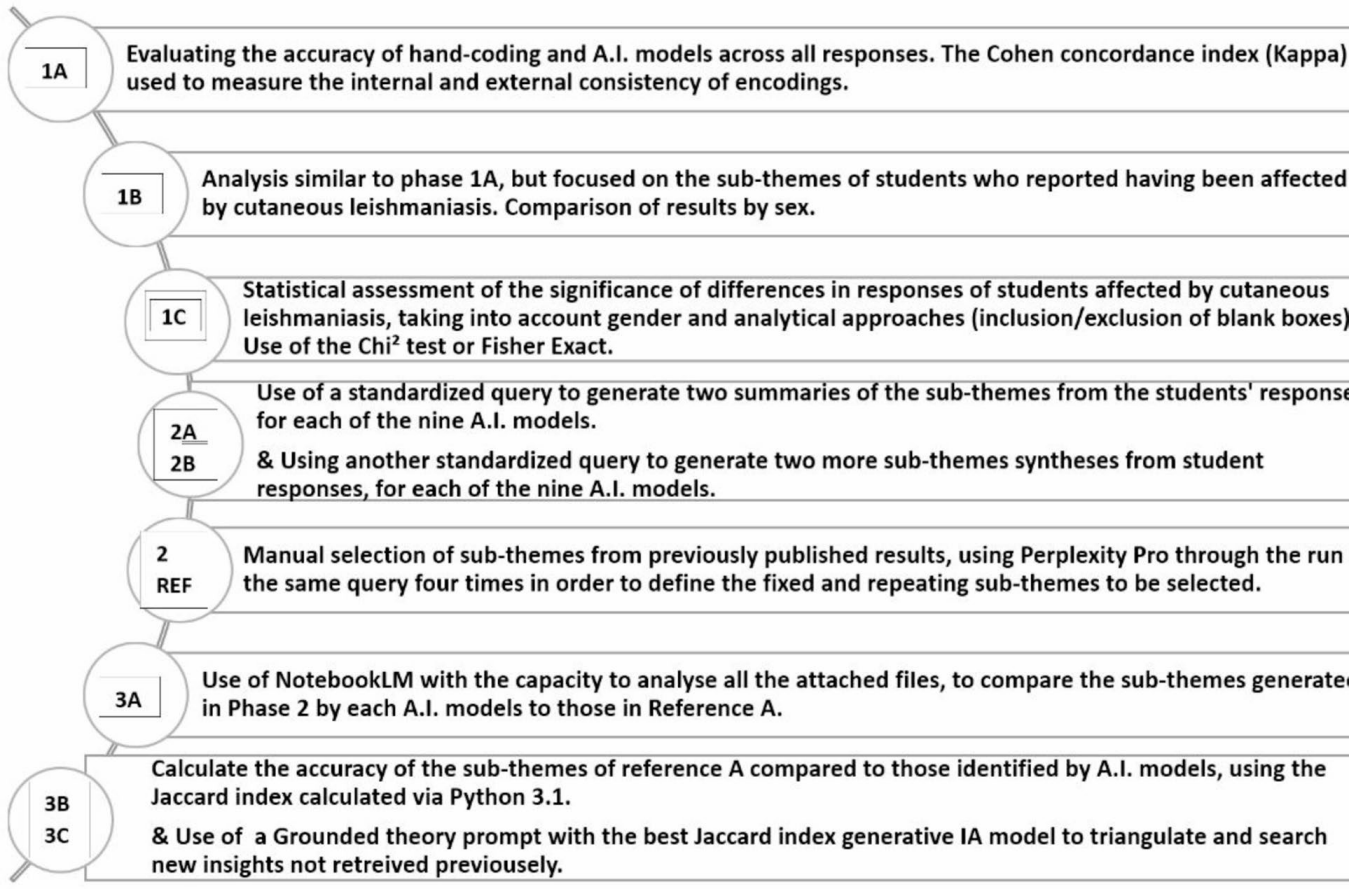
The three phases framework for evaluating and comparing AI-generated sub-themes for this study

This process involved three main phases, as shown in Fig. 1.

#### Phase 1A: analysis of the accuracy of qualitative coding of student responses

In the first step of the analysis, great attention was paid to the qualitative coding of the 448 respondents’ answers to a single open-ended question: “Can you write a short sentence about the likely psychological state of the person with the cutaneous leishmaniasis scar?

Each response was coded independently, using five categories: 1: Negative psychological effect; 2: Normal effect or no effect; 3: Mixed effect between negative and normal; 4: No specific response to the question; 0: No response (empty box). The nine generative A.I. models were used during this phase to analyse the quotes twice (coded 1st, 2nd), overwriting the results of the first analysis before launching the second to avoid any learning effect on the model. Moreover, the A.I. models were used for their paid versions except for Llama and DeepSeek, which are fully available in open source. An advantage was given only to the manual coding done by the second researcher by comparing the results of its first analysis (Man_1st) with the results of reference A (Taken from the previous results published [20]) and by requesting that the second analysis be carried out only by targeting the responses subject to discordance (Man_2nd). This procedure guaranteed a systematic improvement in external consistency with the reference results to ensure that the results of the second analysis were more consistent with those of the second analysis by manual coding.

However, the same prompt was formulated for the A.I. models without prior learning (See prompts of Phase 1A in Supplementary material 3). The results obtained were saved in Excel or text CSV format. Analysing the data was accompanied by a video capture to record the process. Carrying out the same analysis twice for each model made it possible to compare the internal consistency of all the students’ responses (Supplementary material 1). For instance, a cross-classification of all students’ responses was facilitated using Cohen’s kappa index, which was used to determine how well specific patterns derived from internal and external coding performed compared to those derived from the reference codebook (Supplementary material 4).

Nevertheless, to understand students’ experiences with cutaneous leishmaniasis and gender aspects, phase 1B was performed.

#### Phase 1B: analysis of the accuracy of the qualitative coding of the students’ responses declared to be affected by cutaneous leishmaniasis, comparing them according to gender

For this new analysis, only the subgroup of 79 students who declared themselves affected with cutaneous leishmaniasis was targeted. To calculate the Cohen Kappa using the Jamovi software, a new Excel file was prepared to include only the data selection about the targeted students (Supplementary material 5).

#### Phase 1C: analysis of the significance level between the students’ responses declared affected by cutaneous leishmaniasis, comparing them by gender about the analysis methods used

Qualitative analysis form is assessed by qualitatively assessing the following variables (gender, types of response and analysis models) for each response option to a participant on the presence or absence of a psychosocial effect related to the psychosocial consequences of CL sufficed that the variety number is limited. The types of categories have been reduced as follows:

- (P) Presence of psychosocial effect. Re-categorisation 1 to P (Psychological effect).
- (N) No or maybe of psychological effect (N). Re-categorisation 2 or 3 to N (No psychological effect).
- (U) No specific reply to the question or no reply at all. Re-categorisation 0 or 4 to U (Undecided).

In addition, the types of responses were considered by analysing first all the seventy-nine students who said they had been affected by cutaneous leishmaniasis (Supplementary material 6). Then, by analysing, in the second round, only sixty-three students (Supplementary material 7 excluding the sixteen empty boxes considered to have no response). Data were analysed using Jamovi software v2.5.4, and the statistical significance was calculated using Chi-squared or Fisher exact test provided the p-value was below 0.05. The software results of this Phase 1 are reported in Supplementary material 8 and Supplementary material 9.

#### Phase 2: qualitative summary of themes and sub-themes

The second phase of the analysis was specific to the A.I. models and aimed to verify their capacity for precision in the qualitative synthesis of themes and sub-themes about the published results. This phase included an assessment of the robustness of the A.I. responses compared to the reference framework. The results were reached using two prompts, available in Supplementary material 10.

The **method 2‐1 prompt** was done twice for all the nine A.I. generative models (After each completion, prior results were deleted before rerunning the same prompt). As a result of this prompt, two file texts were created per model, recorded as PDF files known as “1st” and “2nd”. Meanwhile, **method 2‐2 prompt** used another unified request, leading to two additional PDFs named “3rd” and “4th”. To better understand this process, two video demonstrations are available in [32] and [33].

The logbook results from Llama 3.1 405B were coded as Model “B”. NotebookLM results coded Model “C”; Gemini 1.5 Advanced Ultra results coded Model “D”; Claude 3.5 Sonnet results belong to Model “E”; ChatGPT o1-Pro results fall under Model “F”; ChatGPT o1 results coded Model “G”; GrokV2 were coded as Model “H”; DeepSeekV3 coded as Model “K” and finally, Gemini 2.0 Advanced coded as Model “M”.

Phase 2 **Reference A’s prompt** was introduced in a separate A.I. model named Perplexity Pro to independently develop this Reference A themes and sub-themes [34]. Indeed, using structured prompts (Supplementary material 11) that synthesise information from the published peer-reviewed text and framework previously included in the Bennis et al. 2017 article and presented in Supplementary material 12. Four iterations of the same prompt were made using the Perplexity model to cover the targeted results shared between the four successive prompts generated, as shown in [35]. This approach aims to ensure consistency with previously established knowledge while leveraging A.I.‘s potential for systematic thematic synthesis and organisation.

#### Phase 3: comparative analysis of the sub-themes accuracy of the synthesis by models B, C, D, E, F, G, H, K and M supported by A.I. Compared to reference A

**Phase 3A** allowed the comparison of the 24 sub-themes of reference A to each of Models B, C, D, E, F, G, H, K and M. Indeed, the results of phases 2‐1 and 2‐2, based on the initial file containing all the students’ responses, enabled each model to generate four thematic analyses noticed 1st, 2nd, 3rd, and 4th. A response matrix (Supplementary material 13) included the 24 sub-themes of reference A and for each column as a variable, the sub-themes 1st, 2nd, 3rd, and 4th of each model, in addition to the three following combination 1st + 2nd, 3rd + 4th and 1st + 2nd + 3rd + 4th. Apart from this, each of the four models’ thematic analysis and their combinations were compared to the 24 sub-themes of reference A using a P/A matrix defining each sub-theme as either ‘Present’ or ‘Absent’. The comparison was made possible by employing the NotebookLM model. This model involved uploading at the same time all four PDF files (1st, 2nd, 3rd, and 4th) of each of the nine A.I. models as resources, with the adapted Canvas comparison with Reference A (Supplementary material 14).

Then, a prompt for Phase 3A (see Supplementary material 15) was applied systematically for each specific model. By introducing the “X” letter, there was no need to replace manually for each prompt the specific model letter B, C, D, E, F, G, H, K, and M. (as shown in the video demonstration) [36]. It should be noted that using NotebookLM was motivated by being the only model that could accept more than 50 resources as attachments for the same project, which helped the reproducibility of the results by rerunning the same repetitive prompts. Moreover, the possibility of selecting precise resources each time was perfect for avoiding any unintended learning that could influence the generation of specific model results.

Then, **phase 3B**, calculated the accuracy of the sub-themes identified using the Models supported by A.I. compared to the reference results (A) with the application of **Jaccard’s index**.

Indeed, Jaccard’s index is defined as the ratio between the intersection and union of the sets of reference sub-themes concerning the sub-themes of each of the models used by applying the following formula: J (A, X) =∣A∩X∣ / ∣A∪X∣.

The Jaccard index is a widely used statistical measure for assessing similarity between sets, particularly in information retrieval and text mining [37]. This index calculates the intersection ratio to the union of two sets, yielding a value between 0 (no similarity) and 1 (perfect similarity). Its scope covers the most superficial keyword comparison to the more complex levels of entire documents, especially concerning document clustering and text mining. It has simple computations and excellent results in comparison of various text similarities in many fields of analysis and retrieval of information [38, 39]. In phase 3B, the Jaccard index was calculated in this current study based on the Excel file collected Supplementary material 16, helping to use the algorithmic code shared in Python version 3.13.0, as notified in Supplementary material 17.

#### Grounded theory for new framework insights

Based on the external reviewers’ suggestions, A final phase 3C was added by developing an AI-grounded theory prompt using the most performant AI model and including the 448 initial students quotes (As available in Supplementary material 18). The prompt was created by asking about innovative and explanatory conceptual models using thematic analysis and applying a grounded theory to investigate non-comparable ideas as discussed in the three cited references [3, 5–7]. Then, with the same model, a triangulation prompt was started with this sentence: *‘Triangulate your findings with the following insights while presenting an original and non-classical conceptual framework’* adding all the gathered new additional subthemes generated by the most performant A.I. models reaching the highest Jaccard index in the final step of phase 2 and reported in Supplementary material 19. This triangulation generated new themes and subthemes useful for creating a new framework, including insightful ideas not already presented during the study thematic analysis nor in the published article several years ago [20]. The full process took less than 15 min, as notified in the video demonstration as [40]. The Napkin A.I. generative visual tool was used to develop the proposed framework [41] using the generative synthesis of the results reached (See Supplementary material 20).

The study meets the SRQR (Standards for Reporting Qualitative Research) found in Supplementary material 21 [42].

## Results

Table 1 demonstrates the comparative performance of various AI models in automated qualitative analysis against traditional manual methods. The weighted Cohen Kappa coefficients revealed varying performance levels regarding internal consistency and alignment with the initial reference standard (Reference A). The results showed that Claude_1st, NoteboookLM_1st and Gemini_1st models achieved high weighted Kappa scores in the first evaluation with low inter-evaluation variability. Regarding external consistency with Reference A, the performance across models ranged from moderate to strong agreement. ChatGPT o1-Pro achieved the highest external consistency (0.79 [0.74, 0.85]), followed by Claude (0.78 [0.73, 0.84]) and Llama (0.78 [0.72, 0.83]). Manual analysis showed progression from initial external consistency (0.74 [0.68, 0.80]) to second evaluation (0.82 [0.77, 0.87]).

**Table 1:** The weighted Cohen kappa coefficients with lower and upper values of the A.I. Generative models about their internal coherence and the comparison with the initial reference A for the 448 responses analysed in phase A1

Pair-Wise comparaison	Estimation of internal consistency (1st vs. 2nd)	Estimation of the external consistency with the initial reference A
ManA_1st	0.88 [0.83, 0.92]	0.74 [0.68, 0.80]
ManA_2nd		0.82 [0.77, 0.87]
Claude_1st	0.99 [0.97, 1.00]	0.78 [0.73, 0.84]
Claude_2nd		0.78 [0.73, 0.84]
NoteboookLM_1st	0.93 [0.89, 0.96]	0.72 [0.65, 0.78]
NoteboookLM_2nd		0.76 [0.71, 0.82]
Gemini1.5_1st	0.92 [0.89, 0.96]	0.73 [0.67, 0.79]
Gemini1.5_2nd		0.77 [0.72, 0.83]
LlaMA_1st	0.79 [0.73, 0.86]	0.75 [0.68, 0.82]
LlaMA_2nd		0.78 [0.72, 0.83]
ChatGPT-o1_1st	0.80 [0.75, 0.85]	0.77 [0.71, 0.82]
ChatGPT-o1_2nd		0.71 [0.65, 0.76]
ChatGPT-o1PRO_1st	0.97 [0.94, 0.99]	0.79 [0.74, 0.85]
ChatGPT-o1PRO_2nd		0.79 [0.73, 0.84]
GrokV2_1st	0.78 [0.72, 0.84]	0.66 [0.60, 0.73]
GrokV2_2nd		0.77 [0.71, 0.83]
DeepSeekV3_1st	0.90 [0.86, 0.94]	0.76 [0.70, 0.81]
DeepSeekV3_2nd		0.75 [0.69, 0.81]
Gemini2.0_1st	0.79 [0.74, 0.85]	0.63 [0.57, 0.69]
Gemini2.0_2nd		0.76 [0.70, 0.82]

The results in Table 2 documented specific patterns across gender subgroups in AI-driven qualitative analysis capabilities. Llama 3.1 405B demonstrated consistent external alignment with Reference A (Kappa = 0.82 [0.68–0.97] for the first analysis, 0.83 [0.68–0.97] for the second analysis), maintaining performance across gender subgroups. ChatGPT o1-Pro achieved perfect internal consistency (Kappa = 1.00 [1.00–1.00]) across all subgroups, with consistent external agreement scores (Kappa = 0.81 [0.69–0.94]). Claude 3.5 Sonnet’s analysis of female student responses showed perfect internal consistency (Kappa = 1.00 [1.00–1.00]) and maintained stable external consistency (Kappa = 0.80 [0.52-1.00]). NotebookLM and Gemini 1.5 Advanced Ultra recorded strong performance metrics.

**Table 2:** Cohen’s kappa estimates with lower and upper confidence intervals for external consistency (compared to reference A) and internal consistency (1st vs. 2nd analysis) for all 79 students previously affected by CL (35 females and 44 males) analysed in phase 1B

Model	Kappa_All_1st Vs Ref_A	Kappa_All_2nd Vs Ref_A	Internal_Consistency All 1st Vs 2nd	Kappa_Female_1st Vs Ref_A	Kappa_Female_2nd Vs Ref_A	Internal_Consistency Female 1st Vs 2nd	Kappa_Male_1st Vs Ref_A	Kappa_Male_2nd Vs Ref_A	Internal_Consistency Male 1st Vs 2nd
Man	0.59 (0.42–0.77)	0.77 (0.63–0.92)	0.82 (0.72–0.93)	0.47 (0.15–0.79)	0.76 (0.44-1.00)	0.57 (0.25–0.90)	0.63 (0.44–0.83)	0.78 (0.61–0.94)	0.88 (0.80–0.96)
Claude 3.5 Sonnet	0.66 (0.51–0.81)	0.71 (0.54–0.87)	0.98 (0.94-1.00)	**0.80 (0.52-1.00)**	**0.80 (0.52-1.00)**	**1.00 (1.00–1.00)**	0.64 (0.47–0.81)	0.70 (0.51–0.89)	0.97 (0.92-1.00)
NoteboookLM	0.76 (0.64–0.88)	**0.82 (0.71–0.93)**	0.91 (0.81-1.00)	0.64 (0.38–0.90)	0.78 (0.56-1.00)	0.73 (0.41-1.00)	**0.80 (0.67–0.93)**	**0.83 (0.71–0.95)**	0.97 (0.91-1.00)
Gemini1.5 Advanced Ultra	0.77 (0.63–0.90)	**0.82 (0.71–0.93)**	0.97 (0.92-1.00)	0.78 (0.58–0.99)	**0.88 (0.70-1.00)**	0.90 (0.73-1.00)	0.76 (0.59–0.93)	0.80 (0.67–0.93)	0.99 (0.98-1.00)
LlaMA 405B	**0.82 (0.68–0.97)**	**0.83 (0.68–0.97)**	0.97 (0.92-1.00)	**0.82 (0.51-1.00)**	**0.82 (0.51-1.00)**	**1.00 (1.00–1.00)**	**0.82 (0.66–0.98)**	**0.83 (0.67–0.99)**	0.95 (0.88-1.00)
ChatGPT o1	0.78 (0.64–0.92)	0.70 (0.58–0.83)	0.79 (0.67–0.92)	**0.80 (0.52-1.00)**	0.64 (0.38–0.90)	0.85 (0.63-1.00)	0.77 (0.62–0.93)	0.73 (0.59–0.86)	0.78 (0.62–0.93)
ChatGPT o1_PRO	**0.81 (0.69–0.94)**	**0.81 (0.69–0.94)**	**1.00 (1.00–1.00)**	**0.80 (0.52-1.00)**	**0.80 (0.52-1.00)**	**1.00 (1.00–1.00)**	**0.82 (0.68–0.96)**	**0.82 (0.68–0.96)**	**1.00 (1.00–1.00)**
GrokV2	0.76 (0.64–0.87)	0.79 (0.66–0.91)	0.90 (0.80–0.99)	0.77 (0.56–0.98)	**0.80 (0.52-1.00)**	0.74 (0.50–0.99)	0.75 (0.61–0.89)	**0.80 (0.67–0.94)**	0.94 (0.86-1.00)
DeepSeekV3	0.78 (0.66–0.90)	0.75 (0.61–0.90)	0.92 (0.81-1.00)	0.64 (0.38–0.90)	**0.80 (0.52-1.00)**	0.85 (0.63-1.00)	**0.83 (0.71–0.95)**	0.76 (0.59–0.93)	0.93 (0.80-1.00)
Gemini2.0 Advanced	0.69 (0.54–0.84)	0.73 (0.62–0.85)	0.80 (0.65–0.94)	**0.96 (0.90-1.00)**	**0.80 (0.52-1.00)**	0.85 (0.85-1.00)	0.63 (0.45–0.82)	0.74 (0.60–0.87)	0.80 (0.64–0.95)

The analysis of Table 3 revealed response distribution patterns across two sample sizes (63 and 79 responses). In the 79-response dataset, Claude 3.5 Sonnet, ChatGPT o1-Pro, and ChatGPT o1 demonstrated statistical significance (*p* < 0.05) in both analyses. ChatGPT o1-Pro showed reduced undecided (U) categorisations compared to earlier versions, while Llama 3.1 405B recorded minimal undecided (U) categorisations in the 63-response sample.

**Table 3:** Contingency table of gender difference showing the Chi² for each model comparing two types of responses from females and males of the seventy-nine students affected by cutaneous leishmaniasis and their subgroup of sixty-three students after discarding the sixteen empty responses (The calculation using jamovi software version 2.5.4)

	Gender repartition	1st Results				2nd Results			
		P	N	U	Chi²	P	N	U	Chi²
Ref A for 63 responses	*F = 31*	25	6	0	0.65				
	*M = 32*	23	8	1					
Ref A for 79 responses	*F = 35*	25	6	4	0.14				
	*M = 44*	23	8	13					
Man for 63 responses	*F = 31*	24	7	0	0.01*	27	4	0	0.04*
	*M = 32*	21	4	7		22	4	6	
Man for 79 responses	*F = 35*	24	7	4	0.006*	27	4	4	0.01*
	*M = 44*	21	4	19		22	4	18	
Claude Sonnet for 63 responses	*F = 31*	26	4	1	0.13	26	4	1	0.1
	*M = 32*	21	5	6		21	4	7	
Claude Sonnet for 79 responses	*F = 35*	26	4	5	0.027*	26	4	5	0.02*
	*M = 44*	21	5	18		21	4	19	
NoteboookLM for 63 responses	*F = 31*	24	5	2	0.39	25	5	1	0.28
	*M = 32*	22	4	6		22	5	5	
NoteboookLM for 79 responses	*F = 35*	24	5	6	0.06	25	5	5	0.05
	*M = 44*	22	4	18		22	5	17	
Gemini1.5 for 63 responses	*F = 31*	25	5	1	0.28	24	6	1	0.25
	*M = 32*	22	5	5		23	4	5	
Gemini1.5 for 79 responses	*F = 35*	25	5	5	0.05	24	6	5	0.04*
	*M = 44*	22	5	17		23	4	17	
LlaMA for 63 responses	*F = 31*	26	5	0	0.75	26	5	0	0.36
	*M = 32*	25	7	0		23	9	0	
LlaMA for 79 responses	*F = 35*	26	5	4	0.18	26	5	4	0.12
	*M = 44*	25	7	12		23	9	12	
ChatGPT o1 for 63 responses	*F = 31*	26	4	1	0.09	25	5	1	0.21
	*M = 32*	22	3	7		19	10	3	
ChatGPT o1 for 79 responses	*F = 35*	26	4	5	0.02*	25	5	5	0.04*
	*M = 44*	22	3	19		19	10	15	
ChatGPT o1 PRO for 63 responses	*F = 31*	26	4	1	0.19	26	4	1	0.19
	*M = 32*	23	3	6		23	3	6	
ChatGPT o1 PRO for 79 responses	*F = 35*	26	4	5	0.03*	26	4	5	0.03*
	*M = 44*	23	3	18		23	3	18	
GrokV2 for 63 responses	*F = 31*	24	6	1	0.74	26	4	1	0.52
	*M = 32*	23	6	3		24	4	4	
GrokV2 for 79 responses	*F = 35*	24	6	5	0.13	26	4	5	0.08
	*M = 44*	23	6	15		24	4	16	
DeepSeekV3 for 63 responses	*F = 31*	25	5	1	0.31	26	4	1	0.17
	*M = 32*	23	4	5		22	4	6	
DeepSeekV3 for 79 responses	*F = 35*	25	5	5	0.05	26	4	5	0.03*
	*M = 44*	23	4	17		22	4	18	
Gemini2.0 for 63 responses	*F = 31*	25	4	2	0.11	26	4	1	0.28
	*M = 32*	18	7	7		22	6	4	
Gemini2.0 for 79 responses	*F = 35*	25	4	6	0.01	26	4	5	0.07
	*M = 44*	18	7	19		22	6	16	

(P) Presence of Psychosocial effect. Recoding 1 to P (Psychological effect)

(N) No or maybe of psychological effect (N). Recoding 2 or 3 to N (No psychological effect)

(U) No specific reply to the question or no reply at all. Recoding 0 or 4 to U (Undecided)

F = 31 M = 32 Without analysing empty responses (Students = 63)

F = 35 M = 44 With analysing empty responses (Students = 79)

(*) Chi² or Fisher exact significance level if the p-value is inferior to 0.05

The analysis identified five main themes encompassing 24 distinct sub-themes, as presented in Table 4: Self-Concept (four sub-themes addressing personal identity), Body Image (three sub-themes focusing on appearance), Social Stigma (five sub-themes examining interpersonal effects), Self-Stigma (six sub-themes detailing psychological responses), and Health Seeking Behaviour (six sub-themes covering coping and treatment).

**Table 4:** The 24 sub-themes resulting from the initial thematic analysis mentioned in reference A are used to compare the accuracy of the qualitative synthesis process

Main theme	Sub-theme reference A	Brief explanation of sub-theme of reference A
Self-Concept	Self-Confidence	Loss of self-confidence due to scars
	Self-Esteem	Reduced self-esteem linked to appearance
	Self-Awareness	Increased awareness of physical appearance
	Self-Contempt	Self-loathing because of scars
Body Image	Body Beauty	Preoccupation with body beauty
	Face Appearance	The importance of facial appearance
	Scars Cosmetic Effects	Cosmetic effects of scars
Social Stigma	Family Relationship	Family relationships affected by fear of contagion
	Avoidance by Others	Avoidance by others because of scars
	Social Contempt	Social contempt for scars
	Marriage Difficulties	Marriage difficulties linked to physical appearance
	Fear of rejection	Fear of social rejection and contagion
Self-Stigma	Embarrassment	Feelings of discomfort associated with scars
	Shame	Ashamed of the way you look in public
	Anxiety	Anxiety due to the perception of scars
	Sadness	Sadness linked to physical condition
	Depression	Depression caused by scars
	Suicidal Ideas	Suicidal thoughts associated with scars
Health Seeking Behaviour	Traditional Remedies	Using traditional remedies to treat scars
	Conventional Treatments	Conventional medical treatments are often ineffective.
	Spiritual Healing	Spiritual acceptance of illness (God’s will)
	Coping Strategies	Coping strategies to hide scars
	Psychological Support	Need psychological support to deal with scars.
	Government Intervention	Call for government intervention to ensure affordable care.

Table 5 documented the thematic alignment capabilities of newer A.I. model versions. ChatGPT o1-Pro, ChatGPT o1, GrokV2, and DeepSeekV3 aligned with Reference A in their final iterations, each identifying all 24 sub-themes (Jaccard index = 1.00).

**Table 5:** The Jaccard index values of the models used, alone or combined, in the qualitative analysis compared with reference A using python3.13.0

Model(s)	Jaccard (A, X1_X2)	Jaccard (A, X3_X4)	Jaccard (A, X1_X2_X3_X4)	Shared sub-themes ∣A∩X1_X2_X3_X4∣	Single sub-themes ∣A∩X1_X2_X3_X4∣	The formula for calculating the Jaccard index for four qualitative syntheses of the same model J (A, X)
B: LlaMA 3.1	0.67	0.63	0.79	19	24	19 / (24 + 19–19)
C: NotebookLM	0.54	0.54	0.63	15	24	15 / (24 + 15–15)
D: Gemini1.5 Adv Ultra	0.58	0.71	0.75	18	24	18 / (24 + 18–18)
E: Claude 3.5 Sonnet	0.50	0.83	0.83	20	24	20 / (24 + 20–20)
F: Chat GPTo1 PRO	0.96	1.00	1.00	24	24	24 / (24 + 24–24)
G: Chat GPTo1	0.87	0.96	1.00	24	24	24 / (24 + 24–24)
H: Grok V2	0.92	0.96	1.00	24	24	24 / (24 + 24–24)
K: DeepSeek V3	0.83	1.00	1.00	24	24	24 / (24 + 24–24)
M: Gemini2.0 Advanced	0.87	0.92	0.92	22	24	22 / (24 + 22–22)

‘X’ can be replaced by the letter B, C, D, E, F, G, H, K, or M. Knowing that B represents the LlaMA 3.1 model, C represents the NotebookLM model, D represents the Gemini1.5 Advanced Ultra model, E represents the Claude 3.5 Sonnet model, F represents the Chat GPTo1 PRO model, G represents the Chat GPTo1 model, H represents the GrokV2 model, K represents the DeepSeekV3 model, and M represents the Gemini2.0 Advanced model. The calculation formula used is as follows J(A, X)=∣A∩X∣/(∣A∣+∣X∣-∣A∩X∣)

Finally, the A.I. grounded theory followed in phase 3C allowed us to get new themes and subthemes results presented in Additional file 10qua. Those results were used to create the final framework.

**Fig. 2:**
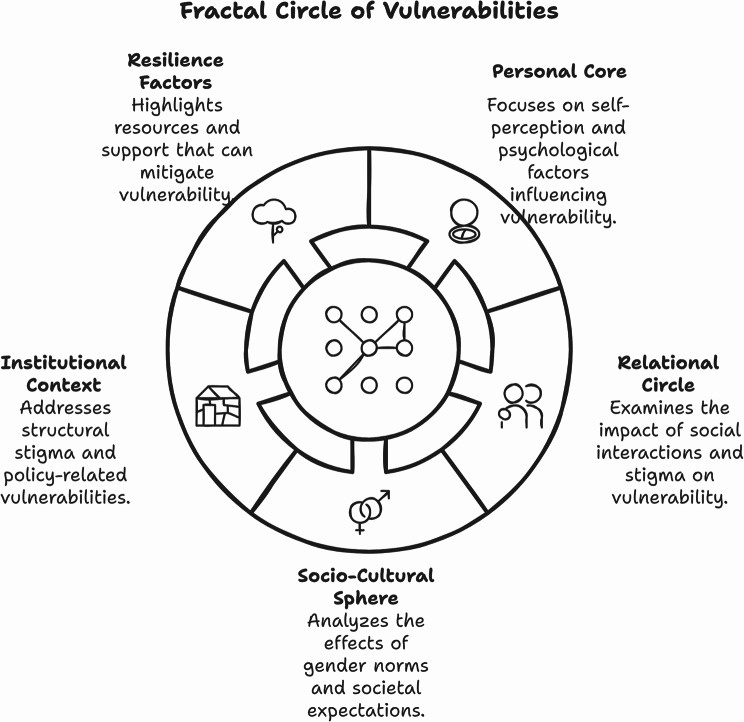
Fractal circle of vulnerabilities framework

The analysis generated the Fractal circle of vulnerabilities framework, an integrated framework for understanding multi-level psychosocial impacts of cutaneous leishmaniasis (Fig. 2), comprising five interconnected spheres: Personal Core, Relational Circle, Socio-Cultural Sphere, Institutional Context, and Resilience Factors. This framework incorporated previously unaddressed dimensions, including stigma by association, structural stigma, and gender-specific experiences.

The framework’s circular structure, highlighting continuous interactions between spheres, introduces a more nuanced understanding of how different levels of influence interact and perpetuate vulnerabilities. Of particular significance are the newly identified elements such as stigma by association affecting family members and close contacts, structural stigma encompassing systemic barriers, and gender-specific experiences highlighting disproportionate impacts on women and girls.

## Discussion

This study assessed the potential of artificial intelligence in performing thematic analysis, emphasising their application to qualitative studies of subjective experiences regarding the self-perceived effects of scarring from cutaneous leishmaniasis. The results show that AI-driven methodologies improve consistency, reproducibility and generalisability compared to standard qualitative analytical methods.

### The accuracy and consistency of A.I. evolutive models

The accuracy and consistency of A.I. revolutionaries’ models showed significant improvements in accuracy and stability (Especially the Gemini and ChatGPT models). ChatGPT o1-Pro excelled through the analysis compared to other A.I. models reviewed. Quantifiable examples of these performance differentials were realised via their weighted Kappa coefficients and their Jaccard indices, widely accepted measures of trustworthiness of analytics [1]. One important insight related to treating ambiguous responses: earlier model versions were much more prone to label responses as undecided (U), especially when complex data were involved. This addresses the notable improvement of new models’ ability to process topographic-qualitative in-depth analysis and better understand psychosocial behavioural transformations [43]. This enhancement of analytic capacity fulfils a necessity for the reliability of such A.I. responses categorisations.

Quality control processes in terms of development or making a conclusion based on the potential of reproducibility of the analysis more and more in some models is needed urgently [16]. For example, it would now be possible to conduct sentiment analysis using the most advanced linguistic models, especially those targeting social media like Grok and Llama, which was not the case with LLM just three years before [44]. A worldwide race for creating more advanced reasoning A.I. generative models capable of conducting delicate, sentimental qualitative data analysis has not yet been reached [45]. Nevertheless, based on the Jaccard index, it is easy to confirm that in this recent study, the latest versions of new A.I. models (ChatGPT, Gemini, DeepSeek) over the past two through four months tend to be more accurate for any qualitative analysis. Therefore, in future, there will be more pre-trained models and fewer manual prompts for an easier accuracy analysis and review [17, 46].

### AI-assisted triangulation efficacy

Triangulation is an indispensable qualitative technique that enhances the validity of qualitative research findings by combining multiple methods or data [47]. This study on A.I. application for thematic analysis takes an approach to triangulation that requires the description and understanding of the setting within which traditional thematic synthesis is done first [48].

This study shows that A.I. models can serve as automated triangulation, making analyses go beyond the initial data and producing more interpretative frames or hypotheses [48]. These models could translate languages and dialects spoken in similar sentences (or quotes) like Moroccan Darija, Moroccan Amazigh, Moroccan Arabic or French without much problem. An anticipated rise in iterations was expected to affect quality control measures and triangulation positively.

### A.I. grounded theory insights

The term “AI-augmented grounded theory” marks a methodological development worth noting regarding how A.I. models can aid and improve grounded theory approaches in qualitative research [49]. Depending on the actual capacities of each model, about accepting or not external files, how many and what size with or not easy to give additional commands or instructions to the unified prompts, the A.I. results be deep and/or detailed.

Then, the decision to keep only the four models that reached the full Jaccard index allowed a very strong synthesis of new insights and helped to create the new Fractal circle of vulnerabilities framework developed through this hybrid approach. The consistency in this framework (Fig. 2), especially concerning gender-specific studies, implies the facility to understand the difference between psychosocial effects and the existence of such psychosocial effects, jumping the possibility of over-classification to explore some of these ideas and constructs that stress resilience. Indeed, resilience points towards broader ways to deal with individual experiences and structural conditions associated with CL vulnerabilities. Such evidence is particularly salient in gender-specific analyses, where A.I. has demonstrated its capacity to capture nuanced differences in lived experiences, a process vital to grounded theory development [14] and methodology [7]. Moreover, the captured subthemes based on the quote analysis included all the aspects selected, like what was presented in the systematic review exploring the cultural effects of gender on perceptions of CL [50]. This proposed way to deal with this qualitative phenomenon using A.I. is based on the concept first proposed in 2021 that focused on developing harmonious coexistence and collaboration between A.I. generative models and humans in qualitative data analysis [51].

### CAQDAS vs qualitative -AI systems

The arrival of AI-driven analytical tools has posed new methodological challenges for CAQDAS software, which has traditionally faced resistance from anthropologists and sociologists alike. One major concern is whether qualitative sampling can be representative when using A.I, such as ones employed specifically for context-sensitive research. As critics would argue, focusing on maximal variation sampling may hamper generalising broader insights from qualitative studies beyond immediate field contexts, thereby making their results hardly transferable to larger populations [52].

A new AI-based generative model targets qualitative researchers to enable them to analyse larger volumes of qualitative data and improve its quality, coverage and importance. Additionally, such A.I. generative models could be applied in many other health disciplines, and most recent AI reasoning models achieved results exceeding human physicians’ reasoning without any language or communication barriers [53–55].

### Prerequisites for AI qualitative research practice

For instance, incorporating A.I. into qualitative research requires adapting teaching approaches and revising course curricula. Tools like the SRQR (standards for reporting qualitative research) checklist ensure that A.I. integration maintains transparency and reproducibility [56]. This is the reason that researchers need to give a detailed explanation about their A.I. models used during the whole or a part of the qualitative analysis, what they can and cannot do and how they fit in with the classical approaches, for more rigorous thinking on qualitative methods based on non-ordinary experiences [57]. Researchers may need to foster other skills, such as A.I. triangulation, to read and assess the quality of such findings. Other than this, A.I. has the potential to help streamline some aspects of qualitative analytic processes by thereby minimising the number of investigators’ heterogeneity while maintaining human analysis depth.

### Limitations and prospects

Some limitations need to be discussed in the context of this study on responses supported by generative A.I. toward cutaneous leishmaniasis scars. Though, these findings show significant progress in AI-assisted qualitative analysis, in a particular geographically and culturally specific context of cutaneous leishmaniasis, replication of this study in other geographical and cultural contexts will validate the observations made. This extension would be especially helpful in elucidating how A.I. models work through different socio-cultural manifestations of the psychosocial impact globally. In addition, planning to use an A.I. algorithm to enable qualitative research method, special care must be taken for bias regarding the A.I. algorithms to be used, with full access to the used prompts, videos demonstrations and reproducibility of the analysis depending on the introduced modalities, categories or variables and the targeted outcomes from A.I. that should match the researchers main objectives, as well as being able to decide how to divide the labour of time and effort between A.I. and human [58]. This includes limitations into researcher bias, respondent bias, and social desirability bias, as well as how AI may mitigate or exacerbate these biases.

Another limitation methodologically manifested itself in the analysis is linked to the Llama 405B model that presented a distinguishing analytical pattern, especially when handling the 63-response sample, where it showed remarkable decisiveness by reducing undecidable categorisations, lowering uncertain categories and demonstrating a high ability to make binary distinctions between presence and absence of psychosocial effects. However, this decisiveness must be taken caution for fear of its potential over-classification [59]. Another weighty consideration is that A.I. technology is fast advancing. The findings represent what A.I. can do at a given time. However, as shown in the results section for Gemini and ChatGPT, future versions may have better features and advancements. Accepting or not using A.I. generative tools by senior university researchers or by researchers with high research productivity could be a subject of debate linked to the ethics of using A.I. in qualitative research [60]. Further research should aim to conduct wide-ranging studies within diverse cultural and linguistic backgrounds, examine A.I. performance across different health conditions and psychosocial contexts, and establish standardised frameworks for evaluating AI-supported qualitative research. Some productions are already under review, and more predictable ones will follow shortly [61, 62]. This would broaden the reliability and usefulness of AI-enhanced qualitative analysis in healthcare research for a better publication with the highest influential impacts rather than citations.

## Conclusion

This comprehensive evaluation of nine A.I. models analysing psychosocial perceptions of cutaneous leishmaniasis offers robust evidence for the transformative potential of generative artificial intelligence in qualitative research. Based on the three phases, the proposed study method could be applied to assess the accuracy and consistency of future A.I. models including deep learning process. For example, sophisticated deep learning models that will follow ChatGPT o1-Pro (Such the upcoming o3 orient, DeepSeek R) will be expected to have higher positive correlation between qualitative analytic precision and depth of understanding of people’s complex experiences. The finding argues that the relationship between AI capabilities and human experience needs to be synergetic for the best qualitative research outcomes, which should be thoroughly investigated and overseen by human qualitative experts for any definitive validation. Finally, it is essential to develop a standardised guidelines expanding the items to do for A.I. qualitative research or reporting A.I. conceptual frameworks, to facilitate standardised broader use in various worldwide research contexts.

## Electronic supplementary material

Below is the link to the electronic supplementary material.

Supplementary Material 1: Additional file 1bis. Phase 1A Full database 31 12 2024

Supplementary Material 2: Additional file 0. English translated quotes

Supplementary Material 3: Additional file 1. Prompts used in Phase 1A

Supplementary Material 4: Additional file 1ter. Phase1A Kappa Cohen R calculation 31 12 2024

Supplementary Material 5: Additional file 2. Phase 1B Analysis of 79 students binary M & F affected by CL 31 12 2024

Supplementary Material 6: Additional file 3. Phase 1C Analysis of 79 students with CL coded P N U 31 12 2024

Supplementary Material 7: Additional file 4. Phase 1C Analysis of 63 students with CL Coded P N U 31 12 2024

Supplementary Material 8: Additional file 3bis. Phase 1C 79 Students with CL PNU Jamovi results 31 12 2024

Supplementary Material 9: Additional file 4bis. Phase 1C 63 Students with CL PNU Jamovi results 31 12 2024

Supplementary Material 10: Additional file 5. Prompts used in Phase 2‐1 and Phase 2‐2. Additional file 5bis. Phase 2‐1 Claude Sonnet 3.5 1st video demonstration. YouTube [32]. Additional file 5ter. Phase 2‐2 Gemini 2.0 Advanced 4th video demonstration. YouTube [33]

Supplementary Material 11: Additional file 6. Phase 2 Reference A Prompt Perplexity sub-themes generation

Supplementary Material 12: Additional file 6bis. Phase 2 Reference A Text extracted from the primary article. Additional file 6ter. Phase 2 Reference A Perplexity results video demonstration. YouTube [35]

Supplementary Material 13: Additional file 7. Matrix of comparative results of reference A Vs other generative A.I. models

Supplementary Material 14: Additional file 7bis. Simplified X Matrix to compare with Reference A themes and subthemes

Supplementary Material 15: Additional file 8. Prompt Phase 3A. Additional file 8bis. Phase 3A All A.I. Models results 2025-01-06-video demonstration. YouTube [36]

Supplementary Material 16: Additional file 9bis. Jaccard index raw database

Supplementary Material 17: Additional file 9. Phase 3B Code Python used to calculate Jaccard Index

Supplementary Material 18: Additional file 10. Phase 3C Grounded theory AI prompt development

Supplementary Material 19: Additional file 10bis. FGHK Additional themes & sub-themes insights issued from Phase 3A. Additional file 10ter. Grounded theory analysis 2025-01-06 video demonstration. YouTube [40]

Supplementary Material 20: Additional file 10qua. Phase 3C Grounded theory AI framework results

Supplementary Material 21: Additional file 11. SRQR checklist

## Data Availability

Data is provided within the manuscript and supplementary information files.

## Abbreviations

A.I.: Artificial intelligence (Generative)
CAQDAS: Computer-assisted qualitative data analysis software
CL: Cutaneous Leishmaniasis
LLMs: Large Language Models
NLP: Natural language processing
SRQR: Standards for Reporting Qualitative Research
