# Supplementary material for: Advancing AI-driven thematic analysis in qualitative research: a comparative study of nine generative models on Cutaneous Leishmaniasis data
Source: BMC Med Inform Decis Mak. 2025 Mar 10;25:124. doi: 10.1186/s12911-025-02961-5 (PMC11895178; doi:10.1186/s12911-025-02961-5)
Supplement: Supplementary file 2 — Supplementary Material 2: Additional file 0. English translated quotes [file 12911_2025_2961_MOESM2_ESM.pdf]

Here are the 454 written responses from students to the following open-ended question:

FR: Female respondent from Rissani Moulay Ali Cherif High School, Errachidia Province (predominantly Arabic speakers).

MR: Male respondent from Rissani Moulay Ali Cherif High School, Errachidia Province (predominantly Arabic speakers).

FT: Female respondent from Tinejdad Ferkla High School, Errachidia Province (predominantly Amazigh speakers).

MT: Male respondent from Tinejdad Ferkla High School, Errachidia Province (predominantly Amazigh speakers).

Symbols used:

(+): This symbol preceding a participant's code indicates that the respondent has reported in another previous questionnaire answer being affected by cutaneous leishmaniasis.

(*): This symbol preceding a participant's code indicates that the respondent has reported in another previous questionnaire answer an uncertainty about whether they have been affected by cutaneous leishmaniasis.

Six students refused to participate in the study, corresponding to the following response numbers: 162, 163, 164, 165, 393, and 394. Then, from 454 students 448 participated

FR001: In my opinion, cutaneous leishmaniasis creates a complex psychological state in affected individuals, particularly in women. Women are more concerned about their faces and fear that a prospective suitor may withdraw after seeing the scars.

FR002: The psychological state of an affected person may become highly complicated and critical.

+FR003: In my view, a person affected by this disease will feel a weakened psychological state and lose confidence in their beauty.

FR004: The disease leaves visible scars on the affected individual. However, I am unsure if it influences their psychological state; you would need to tell us.

FR005: The individual remains deeply embarrassed because the scars and effects of the disease appear on their body, particularly on the face.

FR006: The likely psychological state of an individual with these scars is very poor—absolutely unbearable.

+FR007: The psychological state of an affected person is characterised by discomfort and embarrassment, especially when the disease occurs in an area that cannot be concealed.

+FR008: They will feel embarrassed because of the appearance of disfigurements, particularly on the face.

+FR009: It influences me whenever I meet my friends; they stare at my scar, which diminishes my value in their eyes.

+FR010: The affected individual questions, "Why am I afflicted with this disease while others are not?" without diminishing their own worth.

+FR011: The feelings of fear and anxiety about not finding a treatment for the disease affect the psychological state of the affected person.

+FR012: An individual suffering from cutaneous leishmaniasis endures multiple psychological issues, the most significant being sadness, depression, and constant anxiety.

FR013: They feel ashamed of the disease's effects and experience a sense of alienation.

***FR014**: In my opinion, this disease influences the psychological state of the affected person. When you are afflicted, the disease leaves its effects, and the major problem is that these scars remain on your face. You lose self-confidence and think, "I have lost my face." The face is extremely sensitive.

FR015: Empty response.

FR016: Empty response.

FR017: The affected person is in a very bad state because this disease brings shame and may prevent them from maintaining a good condition. This disease disfigures the skin on any affected part.

FR018: The psychological state of a person affected by these scars is slightly poor. This is because the scars distort beauty, particularly in women, as women care deeply about their outward appearance. If these scars appear on the face, the girl feels ashamed to show her face and may even avoid leaving the house, worsening her psychological suffering.

FR019: The affected person experiences a psychological collapse due to fear of disfigurement and the marks left by the disease, knowing these scars will not disappear.

FR020: The affected person does not feel peace of mind due to fear and a sense of unease.

FR021: I would say that the psychological state of an affected individual, whether male or female, with these scars may lead to psychological disorders that prevent them from going out in public, especially if these scars are on the face of a girl.

FR022: The psychological state of an affected person may weaken. They may feel their face is deformed and feel ashamed to leave the house or engage in similar activities. This could manifest as a psychological illness.

FR023: The affected individual feels a degree of embarrassment, particularly if the scar is on the face.

FR024: I believe that cutaneous leishmaniasis affects the psychological state of the afflicted. However, there are some remedies to prevent this disease, such as vaccination or traditional treatments using herbs.

FR025: Once afflicted with this disease, the individual becomes very afraid for themselves.

+FR026: These scars and blemishes have a profound impact on the psychology of the affected person, especially if these scars are located in exposed areas such as the face. In my view, this could sometimes lead to depression and self-rejection, particularly in girls.

FR027: These scars leave devastating consequences on the psychology of the affected individual, especially if the scars are on the face. This can lead to isolation and self-hatred, particularly in girls.

+FR028: Empty response.

FR029: Perhaps.

FR030: The probable psychological state of a man or woman affected by these scars is one of turmoil, especially if the scars are on the face. The individual feels fear and anxiety.

FR031: This disease influences the probable psychological state of the afflicted individual, whether male or female. It leads to a loss of self-confidence and perhaps even a dislike of others at certain times. The affected person may experience depression and a sense of guilt towards their family, thinking, “I am a curse to my family.”

FR032: The psychological state of a person affected by these scars is diminished. They feel devalued because others say, “Why you and not others?”

FR033: In cases of infection with this disease, it is necessary to seek medical assistance urgently.

FR034: If a girl is affected by this disease and it leaves scars on her face, she will be psychologically impacted and feel sadness.

+FR035: I am affected by this disease, and I suffer from its consequences. The scar on my face causes significant problems in my life. I am forced to apply creams before going anywhere to try to conceal it.

FR036: People affected by this disease feel embarrassed, especially if the scars are on their face, particularly for girls.

FR037: Yes, a person affected by cutaneous leishmaniasis suffers greatly, especially when the disease affects the face. For instance, if it affects a girl in her youth, she desires to live

a normal life but faces significant embarrassment and is compelled to apply creams constantly.

FR038: The psychological state of a person affected by cutaneous leishmaniasis scars is somewhat challenging, especially if the scars are on the face. This creates feelings of embarrassment because the scars do not fade over time and remain visible on the skin.

FR039: The psychological state of a person affected by these scars may deteriorate due to the disease. However, this is not always the case, as various factors can cause lesions on the body. Could you suggest treatments for this disease? Thank you.

+FR040: Yes, these scars can affect the person to the point where they can no longer tolerate the disfigurements on their face or body in general. People may mock them, causing them to feel that their beauty is diminished. This is just my opinion.

FR041: If cutaneous leishmaniasis occurs on the face, it can impact the psychological state of the affected person. They will see others with normal faces while theirs is scarred.

FR042: The psychological state of people affected by these scars makes them dissatisfied and sad, particularly girls.

***FR043**: This disease does not only affect the skin but also the psychology of the affected individuals and their families. The question remains: what is the solution to this disease?

FR044: What I can say about the probable psychological state of a person affected by these scars is that they feel a sense of disfigurement and disappointment because of the disease, which leaves marks on their face. Sometimes, affected people end up detesting themselves.

+FR045: The psychological state of a person affected by these scars is negatively influenced because they leave visible marks on their skin. This is particularly true for girls whose faces are disfigured by these scars, resulting in disdain from others.

FR046: I would say that individuals affected by these phenomena should seek a solution at the hospital as soon as symptoms appear.

FR047: Empty response.

FR048: I believe that people affected by this disease should immediately visit a hospital before the disease spreads to others. Hygiene campaigns should also be conducted to ensure a clean environment, free from all diseases.

FR049: Often, the scars on men and especially on women are an obstacle to marriage because these scars are visible, which is unacceptable to a fiancé.

FR050: The psychological state of an affected female can become unstable and complex due to scars and blemishes on her face. This will cause psychological distress every time she encounters people who look at her scornfully and avoid her, leaving her isolated and insecure.

FR051: The affected individual feels burdened by their body, experiencing fatigue, exhaustion, and dissatisfaction both psychologically and physically. They also feel pain in the affected area.

FR052: The psychological state of the affected person may involve sadness, weakness, and a deplorable condition.

FR053: This disease may affect the psychology of the person due to the difficulty of healing scars.

FR054: It can influence the psychological state when the scar is on the face, particularly when the blemishes do not disappear.

FR055: If cutaneous leishmaniasis affects the face, it significantly influences the psychological state, as the scars diminish beauty and may create suffering for some individuals.

+FR056: Empty response.

+FR057: Empty response.

+FR058: This disease affects both men and women psychologically because of the scars on their skin, particularly on the face. They cannot bear this condition.

+FR059: In the case of being affected by this disease, the individual remains in a normal state because the disease does not affect their psychological state, as it is a well-known disease with no impact on the affected person's life.

+FR060: For girls, they feel disappointed and embarrassed if the scars are on the face. As for men, I have no idea.

+FR061: The psychological state of affected individuals includes embarrassment and disappointment.

FR062: The probable psychological states of affected individuals are fear, embarrassment, and shame due to the body deformities caused by the disease.

+FR063: The disease appeared on my right foot, leaving a scar. I was afraid it would spread to other parts of my body. However, thanks to God, I was cured using a herb from the Sahara after numerous medical treatments proved ineffective.

+FR064: Empty response.

+FR065: A normal psychological state.

+FR066: The scars caused by this disease have notable psychological effects. They lead to depression, especially among women when the scars are on their faces and do not fade. This results in self-hatred and self-contempt.

FR067: Empty response.

+FR068: The psychological state of the affected person may deteriorate into depression due to the lack of methods to remove the scars left by leishmaniasis. As someone who has been affected by this disease, I suffer from its consequences. Is there a solution to cure these scars? Thank you.

FR069: Empty response.

FR070: For women and girls affected by this disease, it can be considered a cause and an obstacle that prevents them from marrying.

MR071: An affected individual would experience a difficult psychological state due to the prolonged illness and the loss of various health privileges, especially when scars appear on the faces of females.

+MR072: They feel a sense of shame and discomfort. This disease greatly influences the psychology of the patient, creating negative feelings as they struggle to accept the presence of these scars and blemishes.

+MR073: Empty response.

+MR074: Empty response.

MR075: I think this is a very serious disease.

***MR076**: Empty response.

MR077: Empty response.

***MR078**: A person affected by this disease must adopt all forms of hygiene to avoid it.

MR079: I believe the treatment is available; one only needs to visit the nearest hospital without fear. I hope all patients will eventually recover.

MR080: From my perspective, the psychological state of a person affected by this disease can influence them in society, such as being abandoned by their friends due to fear that the disease might spread to them.

MR081: An affected person experiences fear of the disease worsening and not being cured. They feel disturbed by their appearance when seen by others and fear that friends and family will distance themselves due to the perceived risk of transmission.

MR082: Empty response.

***MR083**: An individual affected by these scars may feel discomfort due to the harm caused by these scars and possible superinfection.

***MR084**: Affected individuals may feel disturbed and ashamed, struggling to accept themselves or their external appearance, especially if the scars are on visible areas such as the face and hands.

+MR085: Depression may result from this disease, especially if scars appear on the face, exposing the person to ridicule and mockery from others.

***MR086**: The individual will feel fear, and it is necessary to provide them with help.

MR087: Scars on the face could lead to psychological distress, making it difficult for the affected person to go outside.

MR088: The disease disfigures the face and leaves scars, causing psychological distress. They may fear appearing unattractive in front of others, which could cause their psychological state to collapse, though this may improve over time.

MR089: The psychological state of the affected individual is not good because the disease significantly influences their psychological well-being.

***MR090**: A treatment for this disease should be available at all urban and especially rural health centres, where this severe illness is common.

MR091: When this disease affects an individual, it causes psychological impacts.

***MR092**: No, it does not affect the individual. The mark lasts only a week. This disease is not significant and does not cause pain as we are accustomed to it.

MR093: Perhaps this disease causes psychological distress in the affected person. Therefore, the relevant authorities or the government must find a solution to this disease rather than leaving it to the public.

MR094: They feel disdain from their friends and themselves, which leads to marginalisation in society.

MR095: The affected person feels fear and shame due to the scars, both within their family and among friends. They also fear negative consequences from these scars.

MR096: The individual affected by these scars may feel some fear during the initial stages of the disease's appearance. However, over time, they may become accustomed to the scars, and it may become a normal part of life.

+MR097: For the affected person, there may be a fear that these scars will remain on their skin for life and that they will not heal.

MR098: The affected person feels fear and weakness due to the scars and concerns about their health.

MR099: The scars might influence the psychological state of the affected person, creating feelings of isolation due to the appearance of their skin, especially if the scars are on the face. This causes the person to feel ashamed and hesitant to integrate into society, which, in my opinion, affects their psychological well-being.

+MR100: In cases of infection with this disease, fever may occur, similar to other illnesses. In addition, they may experience at least three bites but will not contract the disease a second time, according to what I've heard.

MR101: Empty response.

MR102: When spots and scars appear on the face, the individual feels embarrassed and ashamed, often trying to use a handkerchief to hide them from others.

+MR103: Empty response.

+MR104: Since I was affected by this disease, I was very afraid it would spread more extensively across my body and that it might be transmitted to my family.

***MR105**: Fear of facing society arises due to the multiple scars that disfigure their appearance.

MR106: This is a very serious disease caused by accumulated waste. It affects the skin, leaving scars.

MR107: It is a disease transmitted through blood and direct contact. It is very harmful, and I am afraid of contracting it myself.

MR108: The scars cause disfigurement to the individual's appearance, such as on the face and hands. This negatively affects society and can influence the probable psychological state of the affected person. For this reason, we must combat the causes of these scars.

MR109: Empty response.

MR110: The disease negatively affects the individual because its impacts are severe. The person feels ashamed in front of their friends due to the scars.

MR111: Empty response.

MR112: From my perspective, a treatment must be found for this disease because the affected person will carry the scars for the rest of their life.

MR113: Fear of death.

+MR114: They may be exposed to a slow death.

MR115: They may feel a bit of weakness in their personality. Over time, however, they may begin to forget about it, even as the scar remains on their skin.

+MR116: The psychological state of the affected person can be influenced even after treatment, as the problem is that the scars never disappear.

+MR117: They feel anxious and disturbed by this disease because it influences their psychological state and leaves marks on their skin.

***MR118**: Affected individuals should be patient and pray for divine assistance. They should visit their doctor regularly and follow medical advice. Psychologically, they might struggle to face society and may isolate themselves due to the shame caused by the scars.

***MR119**: Yes, these scars influence the mental health of the affected person, who must take precautions to prevent this disease.

MR120: They feel lonely and lack stability in their daily life due to society's negative perception of patients.

***MR121**: When cutaneous leishmaniasis affects a person, they should consult a doctor because it is a serious disease that affects the nerves and leads to immune collapse. It also causes psychological and dermatological effects for a prolonged period, necessitating visits to a psychiatrist.

+MR122: Empty response.

MR123: This disease often affects some individuals by leaving scars that can drive them to the point of suicide.

MR124: The cure is found in the Quran. Additionally, the person can use **Tamaghot**, found in Merzouga.

***MR125**: They should not feel despair because every disease has a remedy, either now or in the future.

+MR126: Empty response.

+MR127: Affected individuals worry about their health because they fear this disease might kill them. Their families also share this fear. The deformities caused by the disease place them in a state of discomfort and shame.

MR128: Empty response.

+MR129: At the time of the infection, the psychological state of the affected person is painful because the disease does not disappear for many years.

MR130: The individual cannot express their feelings to others because of their illness.

+MR131: I do not know much, but when someone is affected by leishmaniasis, they lose sleep, become anxious, and their mother worries about them.

MR132: Yes, because the affected individual cannot talk about their condition to others for fear that they will distance themselves.

***MR133**: Empty response.

***MR134**: The psychological state of the affected person is linked to the scars and other severe illnesses.

***MR135**: Yes.

MR136: Empty response.

+**MR137**: Empty response.

MR138: A person affected by this disease feels fear because of the scars on their face, which cause a slight loss of beauty.

***MR139**: They may develop a psychological disorder if these scars are on the face or in a similar prominent area.

MR140: When the disease occurs, the individual feels intense pain in the affected area and the appearance of a scar.

+**MR141**: During the period when the individual is affected by this disease, they feel fear, which influences their mental state.

MR142: It is understood that rats and mosquitoes are the origin of cutaneous leishmaniasis infection.

MR143: Empty response.

MR144: This disease has no effect on the psychological state of the affected person, in my opinion.

MR145: People must be cautious of mosquitoes as they are the primary cause of this severe disease, as well as rats.

MR146: In the case of cutaneous leishmaniasis infection, the affected person's psychological state may be influenced, manifesting as fear and psychological disturbances.

+**MR147**: Regarding the probable psychological state, there is no effect, even after scars remain from this disease, as there is no significant fear.

+**MR148**: It can be said that cutaneous leishmaniasis is curable; however, after treatment, the scars remain permanently, requiring the purchase of a treatment such as "Cicatrisol."

MR149: In cases where scars are present on the face, the psychological state of the affected person is negatively impacted, and they feel ashamed.

***MR150**: This affected individual cannot admit they are infected with this disease for fear that others will avoid them.

MR151: If a person is affected in the face, the disease may influence their psychological state, causing shame in showing their face, especially in front of women (if the affected person is male). This leads to feelings of unattractiveness, negatively impacting their psychological state.

+MR152: Every disease has a treatment.

+MR153: The affected person always wonders, "Why am I the one who has this disease and carries this mark on my face?" As you know, society and the streets are not kind.

+MR154: Empty response.

+MR155: A completely normal psychological state.

+MR156: Empty response.

MR157: They must exhibit patience.

+MR158: The affected individual feels embarrassed because their skin is disfigured and no longer normal. I wish there were a treatment for this disease that could prevent permanent scars.

MR159: When afflicted with this disease, individuals feel pain and distress. It would be beneficial if solutions were available to prevent this.

+MR160: The affected individual cannot show the signs of their condition to friends because they would not accept sitting with them if they realised they had leishmaniasis.

+MR161: Empty response.

MR162: Refused to participate.

MR163: Refused to participate.

MR164: Refused to participate.

MR165: Refused to participate.

MR166: Empty response.

MR167: Perhaps the individual feels different from their friends due to the blemishes and scars on the affected parts of their body. This may lead to a sense of inferiority and exposure to mockery from others.

MR168: In my opinion, there is no effect on the psychological state of the affected person because there is treatment, even if scars are present. The patient should have no fears if treated.

MR169: The affected person might fear their face becoming disfigured or that scars will persist on their body. They may also worry that the condition will last indefinitely.

MR170: Empty response.

MR171: Empty response.

MR172: I have not personally been affected by this disease, but my friend was. He was completely unsettled both mentally and physically due to the threat posed by the disease. This is why we must eradicate such diseases.

+MR173: The affected person detests the scars on their face and remains anxious. Their psychological and emotional states are affected as they believe this disease has no cure.

MR174: This disease must be treated promptly to avoid its effects on the psychological state of the affected person.

MR175: This disease has no effect on the psychological state because it is not significant. We are used to seeing this disease. The government should find a solution to this disease.

FT176: The psychological state of a person affected by these scars is very poor, especially for girls, as it diminishes their beauty.

+FT177: When the individual sees the scars, they experience a lamentable psychological state and are plunged into suffering, realising there is no treatment.

+FT178: The psychological state of a person affected by these scars is very difficult, particularly for girls, as it diminishes their beauty.

FT179: A person suffering from this disease feels constant distress. For example, you would not want anyone else to experience this disease because it causes so much suffering.

FT180: The psychological state is slightly affected because they fear infecting their family and loved ones.

+FT181: The psychological state of a person affected by these scars is poor, as it leads to feelings of inferiority in the scarred individual.

FT182: The affected person would find it completely unbearable, as it results in a loss of academic performance and motivation.

FT183: The probable psychological state of a person with these scars could either be dangerous or have no effect at all.

***FT184**: I pray to God to heal all those affected by this disease. Amen.

FT185: The appearance of scars and blemishes caused by cutaneous leishmaniasis may influence the psychological state, especially in girls. They may feel inferior and fear showing their appearance to friends. They might develop self-loathing when looking at themselves in the mirror due to the scars and blemishes.

FT186: The appearance of scars and blemishes from cutaneous leishmaniasis may affect the psychological state of the affected person. They may feel too weak to appear in front of their friends with these scars, especially if they are on the face. The individual might believe others are looking at them with disdain, leading to disgust with their own reflection.

FT187: For scars located on the face, especially in girls, these can represent a psychological issue due to the unsightly appearance and the long period it takes for the scars to fade.

FT188: The probable psychological state of a person affected by these scars may include psychological disorders.

FT189: The visual appearance of scars and blemishes from cutaneous leishmaniasis may influence the psychological state of the affected person, particularly if the scars are on the face.

FT190: If these scars are located in an area that can be covered, such as the back, there is no psychological impact. However, if they are in a visible area like the face, there is a psychological effect.

FT191: If scars are in a place that can be covered by clothing, like the back, their psychological impact is lessened. However, if the scars are in a visible location like the face, the psychological effect is very negative.

FT192: The psychological state of the person might collapse, and they might feel ashamed to appear in public with these scars, which could become an obstacle for them.

FT193: To prevent cutaneous leishmaniasis, it is necessary to fight against waste and eliminate it.

FT194: This disease particularly impacts girls when it leaves a blemish on their face. A girl might think it endangers her beauty, influencing her psychological state, especially in our traditional society, which is not kind to those with facial blemishes, as they assume it to be hereditary.

+FT195: Yes, the scars from leishmaniasis leave a severe psychological impact, especially for girls with scars on their faces. The scars cause significant shame. Is there a treatment for these scars? I hope the answer is yes.

***FT196**: Yes, leishmaniasis influences the psychological state of the affected person. When afflicted, one might feel that others are avoiding or distancing themselves. Upon seeing scars on their own face or hands, the person might experience fear and depression, fearing the disease could worsen and become fatal.

***FT197**: Empty response.

***FT198**: I ask God to heal all those affected by this disease as quickly as possible and to spare my family and all Muslims.

+FT199: Personally, I have no psychological issues caused by this disease, even though I have a scar. It has not caused me suffering.

FT200: Empty response.

+FT201: If spots and scars from cutaneous leishmaniasis appear on the face, the affected person will fear confronting this reality. For girls, it may hinder their chances of marriage, as their faces become disfigured, causing severe psychological effects. I hope you can eliminate this disease.

+FT202: In my view, girls are more affected by this disease, especially if it affects the face. They fear for their future and dreams, particularly regarding marriage. In contrast, for boys in our society, scars are not considered an issue.

+FT203: Personally, I was affected by this disease but did not suffer any psychological issues.

FT204: I think this disease does not affect the psychological state of the affected person. Generally, it has no impact in my opinion.

FT205: These scars affect the person on the face or hands and cause other issues. It is advisable to consult a doctor.

FT206: The psychological state of the person affected by this disease is somewhat poor because they feel the disease will persist over time, all due to mosquitoes.

FT207: Based on what I have observed among affected individuals I know, they are influenced by these scars, especially girls whose faces are affected. This makes them lose their beauty, driving them to isolate themselves and avoid hearing any comments or rumours about their appearance.

FT208: I pray for the person affected by these scars to recover. Whether they use medical or traditional treatments, it does not matter to me as long as they recover and avoid developing psychological issues.

FT209: I pray to God for the quick recovery of those affected by these scars and that this disease, cutaneous leishmaniasis, is eradicated.

FT210: The affected person must follow medical advice, including injections, to reduce the effects of these scars.

FT211: From my perspective, the psychological state of the affected person may be influenced, but this depends on the nurse's evaluation and the severity of the disease.

FT212: Based on what I have seen and heard, most girls affected by this disease feel cursed. The scars obstruct their beauty.

FT213: Yes, it is possible that the scars influence the psychological state of the affected individual, especially when the disease heals but leaves permanent marks on the face.

FT214: The psychological state will be significantly affected as scars remain on the skin, particularly on the faces of girls.

FT215: The psychological state of people affected by this disease may be influenced. These scars and blemishes can become obstacles for them. If the disfigurements are on

visible areas, the individuals feel inferior and experience a sense of imbalance in their beauty, threatening their psychological stability.

FT216: The psychological state of the affected person manifests as a feeling of inferiority and even a loss of interest in life because they perceive themselves as different from others.

FT217: The psychological state of the person affected by these scars is marked by disgust because others avoid them. They may also lose hope of finding a treatment for these scars.

FT218: These scars significantly influence the psychological state of the affected individual, particularly if they are in visible areas like the face or hands. The person feels ashamed to go out in public. Many cases involve individuals staying indoors due to the embarrassment caused by these scars.

FT219: These scars greatly affect the psychological state of the affected person, creating a sense of shame when they are identified as someone suffering from this disease.

FT220: The psychological state of the affected person is influenced by a sense of rejection from friends and family.

FT221: In my opinion, this type of disease negatively influences the health of individuals because it leaves blemishes on the skin and may be transmitted from one person to another. This is why it must be combated by all possible means.

FT222: The visual appearance of scars from cutaneous leishmaniasis may influence the psychological state of the affected person, as these scars do not disappear, especially when located on the face.

FT223: The scars influence the psychological state, making the individual very depressed. They also affect their emotions, instilling a sense of shame in showing themselves.

FT224: The person affected by this disease, known as leishmaniasis, will be psychologically affected, sometimes experiencing depression or similar conditions, particularly if they lack the financial resources for scar treatment.

FT225: The affected individual feels anxiety and shame due to the disease and avoids social gatherings out of embarrassment.

***FT226**: The psychological state of the affected individual manifests in irritability and a propensity to anger easily.

FT227: The psychological state of the affected individual might lead them to self-loathing and contempt. They might lose interest in everything, particularly girls, who feel especially embarrassed if the disease affects their face. I hope you meet our expectations in eradicating this disease.

FT228: These scars might influence the psychological state, particularly if the disease persists or if the scars become highly visible and extensive, especially on the face.

FT229: The psychological state of the person affected by these scars may collapse, resulting in feelings of inferiority, weakness, and depression.

FT230: The probable psychological state of an affected girl is more pronounced compared to that of a boy. Scars on the face diminish women's beauty, and Moroccan society remains unaware and unsympathetic.

FT231: Some girls, particularly those with scars on their faces, may resort to unsuitable methods to eliminate these scars. This is generally misguided, as they believe that if these scars remain, they will not be able to marry, worsening their psychological state.

FT232: Yes, they become very irritable, constantly scratch, lose sleep, and feel lonely.

FT233: The affected person becomes anxious and avoids family and friend gatherings due to feelings of shame.

FT234: Cutaneous leishmaniasis is a chronic dermatological disease. It may cause irritability and shame, along with significant fear.

FT235: Empty response.

FT236: We wish for the quick recovery of every patient suffering from this disease, with the help of God.

FT237: Cutaneous leishmaniasis is a recently emerging dermatological disease that causes suffering, particularly in rural Moroccan populations living near forests. It is transmitted by rats, mosquitoes, and insects, as well as water and animals. Solutions exist if the affected person seeks treatment quickly. Otherwise, it leads to depression, persistent fear, shame, and an inability to sit with friends due to fear of mockery.

FT238: The affected person becomes anxious and should recite the following supplication: "O Lord of all people, remove this affliction and heal me. You are the Healer who gives absolute recovery."

FT239: This disease affects the psychological state of girls as the scars impact their beauty. As we know, Moroccan society is unforgiving.

FT240: Empty response.

FT241: Yes, I agree with the people who live in this psychological state, especially girls. Our society is harsh and judges people based solely on their appearance.

FT242: The psychological state may be significantly affected because the disease can embed itself in the skin and spread through scratching. This makes the person irritable and unhappy, weighing heavily on their mental health. We pray to God for the recovery of affected individuals.

FT243: In my opinion, the psychological state of a person affected by these scars can be influenced, leading them to avoid going outside to prevent others from seeing them. They

become introverted, stop communicating with others, and no longer wish to see themselves due to the scars.

FT244: In my view, cutaneous leishmaniasis may influence the psychological state of the affected person due to the scars caused by the disease. The individual might avoid being seen by others and struggle to tolerate the presence of these scars.

FT245: Empty response.

FT246: Empty response.

FT247: Empty response.

FT248: The appearance of scars and lesions from cutaneous leishmaniasis influences the psychological state of the affected person, causing disturbances such as fear, anxiety, feelings of inferiority, and embarrassment.

FT249: They feel a sense of inferiority.

+FT250: In most cases, the appearance of scars and blemishes from leishmaniasis influences the psychological state of affected individuals. For example, girls may feel shame and timidity, particularly when the scars are on their face.

FT251: Empty response.

FT252: It is possible that the appearance of scars and blemishes from cutaneous leishmaniasis affects the psychological state of the affected individual.

FT253: It is advisable to consult a doctor to reduce these scars, as they leave marks on the skin.

FT254: The person affected by these scars may develop an abnormal psychological state, which requires prompt treatment.

FT255: In our daily lives, we see people influenced by these scars, which create a sense of fear about the dangers of the scars. This can lead to psychological illnesses such as depression.

FT256: Everyone should take care of their health and avoid anything that might harm them, such as waste. Whenever we see a mosquito, we should drive it away to prevent this disease.

FT257: The affected person may feel distressed because the disease causes negative health effects and dissatisfaction with their disfigured appearance, further impacting their psychological state.

FT258: Empty response.

FT259: It could be said that the psychological state of a person affected by these scars is entirely normal, and the scars have no impact on their life.

FT260: This disease may appear on a person's skin and remain for a period of time. However, if it is not treated, it can lead to a dangerous situation.

FT261: Cutaneous leishmaniasis is considered dangerous and can lead to the death of the affected person.

FT262: The scars left by cutaneous leishmaniasis negatively influence the individual's state. They develop a psychological complex and feel ashamed to appear in front of friends because these scars are marks of shame and disdain.

+FT263: The effects caused by leishmaniasis clearly influence the psychological state of the affected person. A psychological complex develops, and they feel ashamed to appear in front of their friends.

FT264: The person affected by these scars likely experiences a disturbed psychological state due to constant feelings of isolation and anxiety, which inevitably lead to depression.

+FT265: Without a doubt, the scars left by leishmaniasis negatively influence the psychological state of the affected person. They develop a psychological complex and feel ashamed to appear in front of their friends because these scars are marks of shame and disdain.

FT266: We hope for the recovery of those suffering from this disease, with the help of God's power.

FT267: The psychological state of the affected person can become very critical. They may feel shy and ashamed to leave the house during this contagious disease, fearing that their friends might refuse to associate with them.

FT268: Empty response.

FT269: Empty response.

FT270: Yes, the psychological state of the affected person can deteriorate for several reasons, such as fear of how others will perceive them, especially if they are female. This is a serious disease.

FT271: When affected by this disease, their psychological state worsens, particularly if the scars are on their face. Every time they look in the mirror, they feel inferior and may suffer from chronic psychological crises requiring specialised care.

FT272: The psychological state of the affected person worsens as they fear what others think, particularly girls.

FT273: The psychological impact depends on the personality and beliefs of the affected person. Those with weaker foundations and who place high value on their appearance may be severely affected, possibly leading to madness over the scars. However, individuals with strong faith and a healthy mindset will not experience such fears.

***FT274**: Empty response.

***FT275**: Empty response.

***FT276**: Empty response.

FT277: The psychological state may deteriorate due to this dangerous disease. In some cases, individuals may avoid leaving the house out of fear of being seen.

FT278: I believe there are several methods to combat this contagious disease and prevent its transmission to others. It is essential to visit doctors, especially if women are affected by this disease.

FT279: Women are at greater risk of contracting this disease because they endure a lot to protect their families and often sacrifice themselves for their loved ones.

FT280: Numerous psychological effects afflict those suffering from this disease, including avoiding others and preferring isolation due to frequent criticism about their condition.

FT281: Individuals affected by this disease suffer from psychological disturbances and develop a complex. They avoid people, opt for solitude, and begin to doubt themselves, losing self-confidence.

FT282: When affected by this disease, the individual may have an unstable psychological state, feeling inferior.

FT283: The affected person constantly feels fear and shame, especially if the disease is on the face. They believe they are isolated and feel anxious.

+FT284: We ask you to find a way to prevent this disease, as many people cannot afford treatment for this dangerous illness. We hope for the recovery of all those suffering.

FT285: When affected by this disease, the person must take precautions to avoid contracting additional illnesses resulting from the primary disease.

FT286: Affected individuals feel disfigured by the scars on their bodies. This leads to shame and panic. I support raising awareness campaigns against this disease.

FT287: Cutaneous leishmaniasis impacts the psychological state of the affected person, causing fear and even thoughts of death. Therefore, it is necessary to protect oneself and one’s family. If the disease appears, seeking a specialist doctor is crucial.

FT288: It is likely that a person affected by this disease experiences psychological disturbances.

FT289: Cutaneous leishmaniasis affects the psychological state and can lead to death. To reduce its effects, sufficient medical or traditional treatments should be made available.

FT290: Cutaneous leishmaniasis is a dangerous and widespread phenomenon, especially in cities. It impacts the psychological state of the affected person, causing fear. Awareness campaigns about hygiene are needed. We wish recovery for those affected.

FT291: The affected individual likely suffers from a disturbed psychological state.

FT292: The psychological state of the affected person is marked by panic and intense fear. They avoid meeting people as others begin to avoid them, leading to sadness.

FT293: When people are angry or ignorant, they may develop psychological problems. We ask for God's forgiveness and health.

FT294: Cutaneous leishmaniasis impacts the psychological state, causing fear, even fear of death. Awareness campaigns about the importance of hygiene and preventing wild rats and mosquitoes should be organised. People must protect themselves and their families. We wish a quick recovery for those affected.

FT295: The affected individual's psychological state collapses due to societal judgment and their living conditions. They feel depressed, avoid socialising with friends and relatives, and imprison themselves at home.

MT296: Exclamation point (no additional content).

MT297: Depression and fear of losing beauty, especially in girls, negatively affect the psychological state, including fears about marriage prospects.

MT298: This disease leaves scars on the skin, making the affected individual more aware of changes in their body, leading to psychological effects.

***MT299**: I believe the affected person will experience disruptions in their daily life.

MT300: They should visit the nearest doctor as this disease is very dangerous. I wish them a speedy recovery.

***MT301**: The affected person perceives their appearance as different from others, which may cause psychological disturbances.

MT302: The psychological state of a person affected by this disease is negative. They think, "I am infected, and others will distance themselves to avoid contamination," leading to feelings of loneliness.

MT303: The individual with scars feels ashamed around healthy people. They become angry when they realise that the scars do not fade over time. Their psyche weakens, depending on their level of faith.

+MT304: Empty response.

MT305: The person lives under the psychological weight of societal judgment. This leads to social isolation as they distance themselves from others out of fear of spreading the disease.

MT306: Empty response.

+MT307: The psychological state of the affected individual depends on the location of the scars. Scars on the face cause more distress compared to scars on the hands, for instance. The person suffers more if the scars are on the face.

MT308: Empty response.

MT309: I think the psychological state of the affected individual will be marked by shame.

***MT310**: Isolation from the outside world and loss of self-confidence.

MT311: The appearance of scars may influence the psychological state of the affected person. They feel their appearance is no longer similar to others, leading to feelings of being permanently different. This drives them toward isolation, solitude, and other psychiatric illnesses.

MT312: It can be said that the person affected by this disease will suffer from both psychological and physical issues.

MT313: Forgiveness.

MT314: I cannot explain this because I do not know much about this disease.

***MT315**: The affected person might or might not feel shame.

MT316: Empty response.

***MT317**: Empty response.

***MT318**: Empty response.

***MT319**: Empty response.

MT320: Empty response.

***MT321**: Empty response.

MT322: Empty response.

MT323: Empty response.

MT324: Their psychological state will be highly dangerous.

MT325: Empty response.

MT326: The scars might influence the psychological state of the affected person or even lead to death.

MT327: Empty response.

MT328: If I were to be affected by this disease, I might feel scared and anxious about my condition.

MT329: The scars may influence the affected individual, as they remain on the body and disfigure their beauty.

+MT330: When I was affected by this disease, I developed scars and was very afraid.

MT331: Empty response.

MT332: When affected by this disease, the person often hides because most people avoid talking to them. The individual wants to remove or hide the scars using any possible method.

MT333: The person affected by these scars might suffer from a psychiatric illness because the consequences of leishmaniasis can expose individuals to mental health issues.

MT334: The affected person must inform a doctor immediately to prevent the spread of the disease.

MT335: I will write about the psychological state of individuals affected by leishmaniasis. These individuals may doubt themselves because people talk about them. I hope everyone takes the elimination of this disease and its virus seriously.

MT336: Visiting the doctor, combating rats and mosquitoes, and disposing of waste properly are essential.

MT337: Cutaneous leishmaniasis is difficult to cure and leaves permanent scars and blemishes on the body, altering the affected individual’s natural appearance. This disease causes many psychological problems, reducing the individual’s external beauty and leading to irritation because society sees only flaws and assumes the affected person is negligent, unhygienic, and careless about their body.

+MT338: Combating this disease requires insecticides and plastic surgery campaigns.

MT339: These scars might lead to numerous psychological issues, including depression.

MT340: The scars may influence the psychological state of the affected individuals, causing multiple psychological disturbances in their daily and emotional lives.

***MT341**: Empty response.

MT342: Everyone must use insecticides against mosquitoes and rats that transmit this serious disease. I hope no one is affected, and thank you.

MT343: Empty response.

MT344: The scars, especially those visible on the skin, can cause other illnesses. However, in my opinion, they have no psychological effect unless their size increases,

potentially leading to death, particularly in children and the elderly. Young women, however, might endure it.

+MT345: This disease may affect people if they are physically vulnerable.

MT346: The affected individual suffers from this disease because they fear it might eventually lead to death. I fear this disease myself.

MT347: The person affected should visit a doctor for treatment, if available. I pray to God for the recovery of all those suffering from this disease. Thank you for addressing such issues.

MT348: We ask you to find a solution to prevent this disease, which scares us due to the scars it leaves behind.

+MT349: One must rely on God. As the Quran says: "I place my trust in God."

***MT350**: This disease may influence health first, and then the psychological state, due to the scars and disfigurement it causes. These scars are seen as a handicap or a source of shame in our society. These factors must be considered, and efforts to combat this disease should be strengthened. Thank you very much.

+MT351: Often, individuals affected by this disease are in a normal psychological state, even when they are suffering.

MT352: This disease causes psychological disturbances, including isolation from society, due to fear of mockery, especially from close acquaintances.

+MT353: Empty response.

MT354: Empty response.

MT355: To prevent this disease and its dangerous spread, authorities must fulfil their responsibilities, and the population must actively participate.

MT356: Society tends to avoid individuals affected by this disease, fearing that it may spread to others.

MT357: Empty response.

MT358: The affected person may suffer a psychological crisis, especially if the scars from this disease remain visible on their face, for example.

MT359: The inability to sleep, constant thoughts about the disease, and fear of being affected by it.

MT360: It is likely that the individual will experience some fear and shame in front of friends. They may even avoid sharing meals with family.

***MT361**: This disease is not common in society, and there are several communities where this disease does not exist because they lack the animals that carry and transmit it to humans.

MT362: The scars might influence the individual, particularly if they are located in sensitive, exposed areas and cannot be healed with modern medical treatments. We pray for God's forgiveness and health.

MT363: The psychological state of this person is somewhat difficult. However, some individuals may develop psychological issues, while others remain unaffected.

+MT364: The person affected by this disease may suffer if the scars are located in sensitive areas, such as the face. This causes embarrassment in front of friends, colleagues, and society at large, hindering their academic and professional progress.

MT365: The affected individual fears leaving the house because they might spread the disease to their friends.

MT366: It is possible for the individual to lose their psychological stability within their family.

MT367: This disease might be caused by mosquitoes and lead to itching, leaving scars.

MT368: We are ready to collaborate with doctors to propose a method to eliminate this disease and stop its transmission.

MT369: I have no idea about the psychological state of a person affected by this disease.

MT370: I cannot explain the psychological state of someone affected by this disease. It is too difficult, as each person has their own psychology.

MT371: Empty response.

MT372: I don't know.

MT373: Individuals affected by this disease may suffer from a psychological crisis if the disease leaves scars on visible areas such as the face.

MT374: It is possible for the individual to develop psychological problems if their acquired immunity is compromised.

MT375: The person affected by this disease may not tolerate the blemishes on their skin, which cause uninfected people to avoid them out of fear of contracting the disease. This results in psychological suffering.

MT376: Life becomes difficult due to the psychological state of the affected individual. The scars influence their psychological well-being. Fortunately, this disease has declined compared to earlier times.

MT377: I have absolutely no idea.

+MT378: For example, a sense of shame in front of friends and fear about the beauty of one’s face.

MT379: The affected person feels ashamed to face others and suffers from the disfigurement caused by this disease, especially on their face.

MT380: Empty response.

MT381: I pray to God for recovery. If there are rats in their region, they should lead a campaign to eliminate them. I hope everyone unites to fight this disease.

MT382: It is possible for the affected individual to experience psychological effects due to the loss of immunity.

MT383: I believe the psychological state of the affected person tends toward depression and disturbance. This is due to two main factors: fear of infecting a loved one and fear of the disease itself.

MT384: For the person affected by this disease, their psychological state weakens, leading to significant feelings of inferiority. This may result in psychiatric illness, affecting their academic and social life.

MT385: I wish them a quick recovery.

MT386: The affected person may doubt themselves. The scars influence them and push them to see a doctor for psychological recovery, which may help restore some health. If their psychological state is normal, the scars won’t be an issue.

+MT387: Empty response.

MT388: They fear for themselves and feel anxious. They may not know what to do, leading to significant irritation.

MT389: The psychological state of the affected person depends on the individual. Some may endure it, while others cannot. If they cannot, especially when the disease affects a crucial part of their body, it may cause a psychological complex.

MT390: Empty response.

MT391: Empty response.

MT392: There will be no effect on the psychological state. Long live the King.

MT393: Refused to participate.

MT394: Refused to participate.

MT395: When affected by this disease, one must visit the hospital for vaccination. This disease is very dangerous and leaves scars on affected skin areas. Long live the King.

MT396: For women, these scars create obstacles in their friendships, work, and other aspects of life.

***MT397**: I wish those affected by this disease a quick recovery. Campaigns should be conducted to ensure cleanliness and combat wild rats (in forests). I hope neither I nor others will ever suffer from this dangerous disease, which causes many other problems.

***MT398**: I think this disease is very serious and causes many issues worldwide. I simply want to wish good health to anyone suffering from leishmaniasis. Thank you all.

***MT399**: Empty response.

***MT400**: The person fears others' remarks, leading them to stay at home and feel isolated. More campaigns are needed in under-informed areas.

MT401: Empty response.

MT402: A person affected by this disease will experience a very disturbed psychological state.

MT403: The affected person should wash their scars with soap, eliminate mosquitoes, and clean thoroughly.

MT404: The appearance of these scars might influence the psychological state of the affected individual. They may feel some worry and dissatisfaction, particularly if the disease affects their face.

MT405: As a student, I have seen such situations or phenomena. I encourage everyone to practice daily cleanliness and combat all insects, especially rats and mosquitoes, using insecticides.

***MT406**: They must seek treatment as soon as possible to avoid suffering too much from this disease. May God grant them a quick recovery.

MT407: Even though no efforts have been made to combat this disease, we thank God for sparing us and our families. We pray for the quick recovery of those affected.

MT408: The affected person may feel inferior or believe their external appearance is disfigured and disrupted.

MT409: Their psychological state is disturbed due to facial disfigurement, feelings of isolation, and inferiority.

MT410: We pray for a quick recovery. They should seek healing through certain herbs from the Sahara.

MT411: I can only say that one should consult a doctor as soon as possible. May God grant recovery and treatment.

MT412: The affected person should be patient and not focus on the scars, as they may appear in many places.

MT413: If a young person is affected by this disease on their face, they may be unable to face society due to fear of social discrimination and disdain, as well as other factors that drain their psychological resilience.

MT414: I do not think I can understand the psychological state of someone affected by these scars.

MT415: I can write a brief statement about the probable psychological state of a person affected by these scars: they can overcome this affliction with medication.

MT416: A young person affected by this disease might face psychological issues.

MT417: Empty response.

MT418: I have no information. I wish for recovery and to never experience this disease.

MT419: The scars are not the problem; the problem is you (government). You do nothing; you're just "f."

***MT420**: I don't know.

MT421: Everyone affected by this disease develops red spots, becomes ill, scratches the affected area, and the scratched spots grow larger, leaving scars on the skin.

MT422: I think cutaneous leishmaniasis and its scars influence the psychological state of the affected individual by repulsing their appearance when they compare themselves to others. This may lead to choosing either positive or negative paths, such as using traditional medicines or surgery.

MT423: I know nothing.

MT424: Yes, the person may feel fear of this disease.

MT425: Of course, there may be some fear of this phenomenon, but being cautious and protecting oneself is the best remedy.

MT426: I know someone who has been affected by this disease since the fifth grade, but it did not impact them. The scars disappeared after just a month using traditional treatments like traditional soap, salt, and rosewater.

MT427: Girls are often affected and suffer from cutaneous leishmaniasis, particularly when it occurs on the face, causing a loss of beauty.

***MT428**: Empty response.

MT429: I do not consider cutaneous leishmaniasis to be a dangerous disease. Instead, I view mental illnesses, which impact human life, as truly dangerous.

MT430: Mental illness is the most dangerous; it negatively influences a person’s life.

***MT431**: I wish for the recovery of all affected individuals. Measures should be taken to eliminate wild rats and combat insects to eradicate this disease.

MT432: Empty response.

MT433: These scars might lead to psychological disorders because they do not disappear, and the person might re-experience the same feelings if the blemishes remain visible on their skin.

***MT434**: They continue visiting the doctor for this disease and isolate themselves from family and society as much as possible.

***MT435**: The affected person develops a psychological complex and avoids going outside because their face is entirely covered with large spots.

+MT436: The affected person is very fearful of infecting their family members and avoids spending time with them. They also fear this disease may cause additional illnesses in the future.

MT437: When affected by this dangerous disease, the individual feels significant fear, which leads them to think only about their condition, greatly impacting their psychological state.

MT438: They may feel somewhat isolated.

+MT439: If the disease affects a visible area like the face or hands and worsens without modern medical treatment, it can cause internal psychological depression. This occurs because they do not want others to see their scars.

MT440: These scars might lead the individual to isolate themselves from others, especially if the scars are on their face and visible to others, potentially causing depression.

+MT441: The person may feel embarrassment and shame when leaving the house, especially if the leishmaniasis scars are on the face and highly visible.

MT442: I don’t know. Only God knows. Every disease has a treatment, and healing comes from God.

MT443: The psychological state is lamentable, especially for girls, as they care the most about their beauty and appearance.

MT444: These scars might hinder communication and integration with others as they leave significant marks on sensitive areas of the skin, particularly the face.

MT445: Empty response.

***MT446**: I don’t know.

MT447: I wish them recovery and encourage them to take every precaution to avoid this disease.

MT448: The individual might develop a psychological complex, especially if they are an adolescent, as they feel unhappy about their physical appearance. They view their body as an essential asset to attract girls.

MT449: Psychological disorders, absence of peace of mind, and self-hatred, especially for adolescents like me.

MT450: The affected person experiences psychological distress, fearing they might infect and transmit the disease to their family members.

***MT451**: The affected person searches for the ideal medication. Doctors must work to find ways to combat this disease.

+MT452: The psychological state is marked by complexity because there is no treatment for these scars, and they will remain forever. The person wonders how to make these scars disappear.

MT453: Empty response.

***MT454**: I am unfamiliar with this disease, so I cannot speak about it. I apologise for my ignorance about this condition. "The only thing I know is that I know nothing."
