# Supplementary material for: Advancing AI-driven thematic analysis in qualitative research: a comparative study of nine generative models on Cutaneous Leishmaniasis data
Source: BMC Med Inform Decis Mak. 2025 Mar 10;25:124. doi: 10.1186/s12911-025-02961-5 (PMC11895178; doi:10.1186/s12911-025-02961-5)
Supplement: Supplementary file 3 — Supplementary Material 3: Additional file 1. Prompts used in Phase 1A [file 12911_2025_2961_MOESM3_ESM.pdf]

English translation of the Phase 1 A Prompt

Please classify the responses below from the study participants. The classification should target all 454 responses, from 1 to 454.

All students were expected to answer this open-ended question:
"Can you write a brief statement about the likely psychological condition of a person bearing the scar of cutaneous leishmaniasis?"

Thus, each response from the students is to be coded as **1, 2, 3, or 4**, following an analysis of their answers, as follows:

1 if coded as Bad psychological effect

2 if coded as Normal effect or no effect

3 if coded as Mitigated effect between bad and normal

4 if the response is considered as "No specific response to the question"

0 for case number (15, 16, 28, 47, 56, 57, 64, 67, 69, 73, 74, 76, 77, 82, 101, 103, 109, 111, 122, 126, 128, 133, 136, 137, 143, 154, 156, 161, 166, 170, 171, 197, 200, 235, 240, 245, 246, 247, 251, 258, 268, 269, 274, 275, 276, 304, 306, 308, 316, 317, 318, 319, 320, 321, 322, 323, 325, 327, 331, 341, 343, 353, 354, 357, 371, 380, 387, 390, 391, 399, 401, 417, 428, 432, 445, 453)

The classification should cover all 454 responses, numbered from 1 to 454. However, it should take into account that six students refused to participate in the study, corresponding to the following response numbers: 162, 163, 164, 165, 393, and 394.

Additionally, 76 students did not write anything, leaving their response field blank.

Original used Prompt in French :

Prière de classer les réponses ci-dessous des participants effectifs à l'étude Le classement doit cibler l'ensemble des 454 cases de 1 à 454.

Tous les élèves ont été censé repondre à cette question ouverte : **Est-ce que vous pouvez écrire une petite phrase sur l'état psychologique probable de la personne portant la cicatrice de la leishmaniose cutanée ?**

Ainsi, il est demandé de coder par 1, 2, 3 ou 4 après analyse de chaque réponse de chacun des éléves soit :

1 : si codée comme effet psychologique négatif

2 : si codée comme effet normal ou absence d'effet

3 : si codée comme effet mitigé entre négatif et normal

4 : si la réponse est considérée comme "pas de réponse spécifique à la question"

0 : pour les numéros de cas suivants : 15, 16, 28, 47, 56, 57, 64, 67, 69, 73, 74, 76, 77, 82, 101, 103, 109, 111, 122, 126, 128, 133, 136, 137, 143, 154, 156, 161, 166, 170, 171, 197, 200, 235, 240, 245, 246, 247, 251, 258, 268, 269, 274, 275, 276, 304, 306, 308, 316, 317, 318, 319, 320, 321, 322, 323, 325, 327, 331, 341, 343, 353, 354, 357, 371, 380, 387, 390, 391, 399, 401, 417, 428, 432, 445, 453.

Le classement doit cibler l'ensemble des 454 cases de 1 à 454. Tout en prenant en considération que 06 éléves ont réfusé de participer il s'agit des numéros (162, 163, 164, 165, 393 et 394) à l'étude. De même, 76 élèves n'ont rien écrit en laissant la case vide.

Voici les 454 réponses écrites des élèves à la question ouverte suivante :

FR Répondant Féminin du lycée de Rissani Moulay ali cherif province Errachidia (prédominance des arabophones)

MR Répondant Masculin du lycée de Rissani Moulay ali cherif province Errachidia (prédominance des arabophones)

FT Répondant Féminin du lycée de Tinejdad Ferkla province Errachidia (prédominance des amazigophones)

MT Répondant Masculin du lycée de Tinejdad Ferkla province Errachidia (prédominance des amazigophones)

(+) Ce symbole qui précède parfois le code du participant dans le document sert de mentionner que le répondant a déclaré être touché par la maladie de la leishmaniose cutanée

(*) Ce symbole qui précède parfois le code du participant dans le document sert de mentionner que le répondant a déclaré de ne pas savoir s'il a ou non été touché par la maladie de la leishmaniose cutanée

FR001 Selon mon point de vu, la maladie de la leishmaniose donne un état psychologique compliqué chez la personne touchée et surtout chez la femme. Car les femmes ont plus peur sur leurs visages, peur que le jeune homme qui va se présenter pour demander sa main disparait après avoir vu ces taches.

FR002 Peut être que l'état psychologique de la personne touchée sera très compliquée et critique.

+FR003 Selon mon point de vu, la personne touchée par cette maladie va sentir un état psychologique faible et va perdre confiance en sa beauté

FR004 Elle laisse des marques visibles sur la personne touchée. Mais je ne sais pas si elle influence ou non son état psychologique c'est à vous de nous le dire

FR005 la personne reste très gênée, car les cicatrices et les effets de cette maladie apparaissent sur son corps et surtout au niveau du visage

FR006 l'état psychologique probable de la personne touchée par ces cicatrices est très mauvais. Absolument insupportable

+FR007 l'état psychologique de la personne touchée est qualifiée par la gêne et l'embarrât. Surtout si la maladie se trouve dans une zone qui ne peut être cachée

+FR008 il va sentir la gêne à cause de l'apparition de ses malformations surtout au niveau du visage

+FR 009 Elle m'influence chaque fois que je rencontre mes amies, elles regardent ma cicatrice, cette dernière diminue ma valeur devant les gens.

+FR010 la personne touchée se dit pourquoi je suis malade par cette maladie et les autres gens ne le sont pas et ne diminue pas de leurs personnes

+FR011 la sensation de peur et d'inquiétude de ne pas trouver un traitement à cette maladie est ce qui affecte la personne touchée par cette maladie

+FR012 la personne touchée par la maladie de la leishmaniose souffre de plusieurs maladies psychologiques dont les plus importantes sont la tristesse, la dépression et l'inquiétude permanente

FR013 elle a honte à cause des effets de cette maladie et elle a une sensation d'être une étrangère.

*FR014 selon mon point de vu je dis que cette maladie influence l'état psychologique de la personne touchée. Car quand vous êtes touché par cette maladie il reste des effets de cette maladie. Et le grand problème est que ces marques restent sur ton visage. Tu perds ton auto-confiance et tu te dis j'ai perdu mon visage. Le visage est trop sensible.

FR015 vide

FR016 vide

FR017 le touché se trouve dans un état très mauvais, car cette maladie apporte la honte et il peut ne plus être dans un bon état. Cette maladie déforme la peau sur n'importe partie touchée.

FR018 L'état psychologique de la personne touchée par ces cicatrices est mauvaise un petit peu. Car elles déforment la beauté (dans le cas de la femme) car la femme s'occupe beaucoup de son apparence extérieure. Et dans le cas que ces cicatrices apparaissent au niveau du visage, la fille a honte de monter son visage ou même les cicatrices peuvent l'empêcher de sortir de la maison ce qui augmente sa souffrance psychologique.

FR019 La personne touchée par cette maladie a un psychisme effondré à cause de la peur de la déformation et de l'effet que laisse la maladie quand il saura que les marques ne vont pas disparaitre.

FR020 La personne touchée par cette maladie ne sens pas la paix de l'esprit car elle a un sentiment de peur et de manque de réconfort.

FR 021 Je peux dire que l'état psychologique de la personne touchée homme ou femme par ces cicatrices va conduire à des troubles psychologiques qui vont l'empêcher de sortir à la rue. Et surtout si ces cicatrices sont sur le visage des filles.

FR 022 Il se peut que l'état psychologique de la personne touchée soit affaibli. Il se peut qu'il sente la déformation de son visage et qu'il ait honte de sortir de la maison ou quelque chose de semblable. Et ceci est représenté comme une maladie psychologique.

FR023 La personne touchée sent un peu de gêne Surtout Si la cicatrice et au niveau du visage

FR 024 Jeudi que la maladie de la leishmaniose est une maladie qui affecte l'état psychologique de la personne touchée. Mais malgré cela Il y a quelques remèdes Pour éviter cette maladie comme traité par la vaccination ou traité par l'utilisation des moyens traditionnels et des herbes…

FR025 une fois atteint par cette maladie de la personne a très peur sur soi-même

+FR 026 ses cicatrices et tâches une influence grandiose sur la psychologie de la personne touchée surtout si ces sacrifices se trouvent dans des zones exposées comme le visage. Elle peut conduire parfois selon mon point de vue un état de dépression et le refus du soi (surtout pour les filles).

FR 027 ses cicatrices laissent des conséquences dévastatrices sur la psychologie de la personne touchée est surtout si la cicatrice se trouve au niveau du visage. Cela peut conduire à l'isolement et la haine de soi. Surtout pour les filles.

+FR 028 Vide

FR 029 peut-être

FR 030 l'état psychologique probable de l'homme ou la femme touchée par ses cicatrices est boulversée et surtout si les cicatrices sont au niveau du visage (j'aurai peur) la personne sent la peur et l'inquiétude

FR 031 cette maladie de leishmaniose influence d'état psychologique probable de la femme ou l'homme touché. On est sur la route de la perte de confiance en soi et de déteste les gens peut-être dans certaines périodes certains moments. La personne touchée sent la dépression avec un sentiment de culpabilité de la part de sa famille car il se dit que je suis une malédiction pour ma famille.

FR 032 états psychologiques comparables la personne touchée par ses cicatrices etat est abaissée et diminue de sa valeur les gens lui dit les autres gens ne sont pas touchés pourquoi toi.

FR 033 dans le cas des atteintes par cette maladie il faut aller vers un hôpital de toute urgence

FR 034 c'est la fille est touché par cette maladie est que cette maladie lui laisse des cicatrices sur le visage elle sera psychologiquement affectée et elle sera triste

+FR 035 je suis affecté par cette maladie et je souffre de ses conséquences la cicatrice sur mon visage me crée un grand problème dans ma vie. Je suis obligé de mettre des crèmes avant de partir à n'importe quel endroit pour essayer de les cacher

FR 036 des personnes touchées par cette maladie sont gênées surtout si les cicatrices sont au niveau du visage et surtout pour les filles.

FR 037 oui la personne touchée par la maladie de la leishmaniose souffre énormément car il est touché au niveau du visage. Comme vous le savez ce fut une fille dans l'âge des fleurs elle veut être normale mais elle est exposée à une grande gêne et on l'oblige à mettre des crèmes tout le temps.

FR 038 l'état psychologique de la personne touchée par leishmaniose à travers ses cicatrices est un peu difficile surtout si les tâches trouvent sur les visages ce qui crée un sentiment de gêne car les cicatrices ne disparaissent pas avec le temps et restent visible sur la peau

FR 039 il se peut que la personne touchée par ses cicatrices à un état psychologique détériorée à cause de cette maladie. Mais ce n'est pas d'une manière systématique. Car plusieurs causes peuvent creer ses lésions au niveau de son corps laissant les cicatrices et des tâches. Est-ce que vous pouvez nous proposer le traitement des médicaments pour cette maladie est merci beaucoup

+FR 040 oui ses cicatrices peuvent influencer la personne touchée qui ne pourra plus supporter de voir ces déformations sur son visage où son corps en général. Les gens peuvent se moquer de lui et il peut paraître ses qualités de beauté. Donc il peut influencer l'état psychologique de la personne. C'est juste mon avis

FR 041 si la maladie de la leishmaniose se trouve au niveau du visage cela peut retentir sur son état psychologique. Il va voir que les autres ont des visages normaux alors que son visage et cicatrisé.

FR042 états psychologiques propres de personnes touchées par ces cicatrices vont les rendent insatisfait et tristes surtout les filles

*FR 043 cette maladie n'affecte pas que la peau mais également la psychologie des personnes touchées et leurs proches. La question qui reste posée qu'elle est la solution pour cette maladie

FR 044 ce que je peux dire sur l'état psychologique probable de la personne touchée par ses cicatrices c'est qu'il sent une déformation au niveau de son corps avec un sentiment de déception à cause de cette maladie qui laisse des marques sur son visage. Parfois des personnes touchées finissent par se détester eux-mêmes.

+FR 045 L'état Psychologique de la personne touchée par ses cicatrices l'influence car elle laisse des traces sur sa peau visibles, comme c'est le cas chez Les filles qui ont des visages défigurés à cause de ses cicatrices source de méprise de la part des gens.

FR 046 je peux dire que les personnes touchées par ces phénomènes quand les symptômes apparaissent il doit aller chercher la solution à l'hôpital

FR 047 case vide

FR 048 je peux dire que les gens touchés par cette maladie de la Leishmaniose doivent partir à l'hôpital immédiatement avant que la maladie se transmette à d'autres personnes. Il faut réaliser des compagnes de propreté pour un environnement sain de toute maladie.

FR 049 souvent les cicatrices chez l'homme touché et surtout chez la femme touchée sont un obstacle lors du mariage car sa cicatrice est visible, chose que ne tolère pas le fiancé.

FR 050 il se peut que la situation de la personne touchée féminine soit psychologiquement instable et complexée à cause de la présence des cicatrices et des tâches sur son visage ceci va laisser un trouble psychologique à chaque fois qu'elle voit des gens-là regardant par méprise et l'évitent dans ce cas et sera seule est complexée.

FR 051 il peut sentir qu'il porte un poids sur son corps et il va sentir la fatigue et l'épuisement et la non satisfaction psychologique et corporelle. Il va sentir une douleur dans la zone touchée.

FR 052 l'état psychologique de la personne touchée peut être la tristesse la faiblesse et une situation méprisable.

FR 053 cette maladie peut influencer la psychologie de la personne à cause de la difficulté de la guérison des cicatrices.

FR 054 elle peut influencer l'état psychologique quand la cicatrice se trouve au niveau du visage et surtout quand ces tâches ne disparaissent pas.

FR 055 si la leishmaniose cutanée se trouve au niveau du visage elle va influencer une grande manière l'état psychologique car la cicatrice diminuée de sa beauté et peuvent créer des souffrances pour certaines personnes.

+FR 056 case vide

+FR 057 une case vide

+FR 058 cette maladie influence de l'homme et la femme touchée avec des troubles psychologiques liés à la présence des cicatrices sur la peau et surtout au niveau du visage. Il ne peut supporter cette maladie.

+ FR 059 Dans le cas de l'atteinte par cette maladie appelée Leishmaniose la personne touchée est dans une situation normale car il n'y a pas d'effet sur son état psychologique du faites que c'est une maladie connue qui n'a aucun effet sur la vie de la personne touchée.

+ FR 060 pour la fille surtout il va sentir la déception et la gêne si les cicatrices sont au visage. Mais pour l'homme je n'ai aucune idée.

+FR 061 état psychologique des personnes touchées et la gêne et la déception.

FR 062 états psychologiques probables des personnes touchées sont la peur, la gêne et la honte des malformations que touche le corps.

+ FR 063 la maladie de la leishmaniose s'est installé au niveau de mon pied droit et elle m'a laissé une cicatrice j'avais peur qu'elle commence à se développer dans plusieurs partis de mon corps. Mais grâce à dieu, l'herbe du Sahara j'ai guéri après que les nombreux traitements médicaux que j'ai utilisés n'ont pas donné des effets

+ FR 064 case vide

+FR 065 un état psychologique normal

+FR 066 les cicatrices provoquées des effets notables sur la psychologie de la personne touchée elle laisse une dépression surtout chez les femmes quand elles sont touchées au niveau du visage est que ces dernières ne disparaissent pas ce qui provoque la haine et le mépris du soi-même.

FR 067 VIDE

+FR 068 L'état psychologique de la personne touchée atteint la dépression à cause de l'inexistence de moyens pour faire disparaître les traces de la leishmaniose. Puisque je suis une personne touchée par cette maladie et je souffre de ses conséquences et ce qu'il existe une solution pour guérir les cicatrices ? Merci.

FR 069 case vide

FR 070 exemple chez les femmes et les filles touchées par cette maladie qu'on peut dire que c'est une cause est un obstacle qui les empêche de se marier.

MR 071 Il sera dans un état psychologique difficile à cause de la longueur de la maladie et de la perte de plusieurs privilèges de bonne santé surtout lors de l'apparition de cicatrices au niveau des visages chez les personnes de sexe féminin.

+MR 072 il sent un peu de honte et un malaise. Cette maladie influence de manière très grande sur la psychologie du malade et également à l'égard de ses sentiments négatifs car il n'accepte pas qu'il ait ces taches et ces cicatrices.

+MR 073 case vide

+MR 074 case vide

MR 075 Je pense que c'est une maladie très grave

*MR 076 case vide

MR 077 case vide

*MR 078 la personne touchée doit utiliser tous les types d'hygiène pour éviter cette maladie

MR 079 Je dis que le traitement est disponible il faut juste aller à l'hôpital le plus proche de votre lieu de résidence sans avoir peur de rien. Et je souhaite à la fin la guérison pour tout malade.

MR 080 Selon mon point de vue, je dis que l'état psychologique de la personne touchée par cette maladie peut l'influencer un peu dans la société comme qu'il soit quitté ou abandonné par ces amis par peur que cette maladie les touchent également.

MR 081 la sensation de peur chez la personne touchée par cette maladie, du risque qu'elle s'aggrave et ne guérisse pas. Elle sera dérangée par son apparence quand on la voie ; et la peur que ces amis et sa famille s'éloigne de lui et le rejette par peur d'être touché par la même maladie et sa transmission qui serait probable.

MR 082 case vide

*MR 083 Pour cette personne touchée par ces cicatrices il va sentir une gêne à cause du mal issu de ces cicatrices et de la surinfection.

*MR 084 il se peut que la personne touchée soit perturbée et sent la honte et ne s'accepte pas soi-même ni de son apparence externe surtout si les cicatrices sont visibles sur des zones souvent découvertes comme le visage et les mains.

+MR 085 Il se peut qu'il ait une dépression à cause de cette maladie sur son visage ce qui l'expose aux rires et aux moqueries par les autres.

*MR 086 Il va sentir la peur et il est obligatoire de le sauver

MR 087 il se peut qu'il développe une maladie psychologique si les cicatrices se trouvent sur son visage et il ne va plus supporter de sortir à l'extérieur.

MR 088 la maladie va défigurer son visage et va laisser des cicatrices et le visage ne sera plus sain ce qui va retentir sur son état psychologique, il va avoir peur de paraitre d'un apparence non convenable devant les filles ce qui rendra son état psychologique totalement effondré, mais avec le temps il va s'améliorer progressivement.

MR 089 L'état psychologique de la personne touchée n'est pas bonne, car cette maladie influence beaucoup son état psychologique.

*MR 090 Il faut qu'un traitement à cette maladie soit disponible au niveau de l'ensemble des dispensaires urbains et surtout ruraux où cette grave maladie est fréquente.

MR 091 Quand cette maladie touche la personne, elle lui fera naitre des effets psychologiques

*MR 092 Non elle n'a pas d'effet sur la personne touchée. Il reste juste sa trace pendant une semaine. Cette maladie n'est pas importante et elle ne crée pas de Paine car on est habituée sur cette dernière.

MR 093 Peut être que cette maladie laisse un trouble psychologique chez la personne touchée. C'est pour cela que la commission concernée ou le gouvernement en général doit trouver une solution pour cette maladie et non pas le peuple

MR 094 il se sent être mépris devant ses amis et avec soi-même, ce qui le rend marginaliser dans la société.

MR 095 la personne touchée sent la peur et la honte à cause de ces cicatrices et ceci dans sa famille et avec ses amis. Il a également peur d'avoir des effets négatifs à cause de ces cicatrices.

MR 096 il se peut que la personne touchée par ces cicatrices sente un peu de peur lors des premières étapes de l'apparition de cette maladie. Sauf qu'il va s'habituer sur ces cicatrices avec le temps pour que tout ceci redevienne une chose normale.

+MR 097 pour la personne est touchée par cette maladie, il se peut qu'il y est un peu de peur que ces cicatrices restent durant toute la vie sur sa peau et de ne pas guérir.

MR 098 la personne touchée sent la peur et la faiblesse à cause des cicatrices et elle a peur pour sa santé

MR 099 il se peut que les cicatrices influence sont état psychologique et créer chez la personne touchée la sensation de l'isolement des autres à cause de ces effets sur la peau et surtout s'ils sont sur le visage ce qui rend la personne touchée très honteuse de s'intégrer dans la société. Et ceci selon mon point de vue va influencer son état psychologique.

+MR 100 dans le cas de l'atteinte par cette maladie il y aura l'apparition d'une fièvre comme pour toute les autres maladies, en plus qu'il va être exposé à au moins trois piqures et qu'il ne développera pas cette maladie une deuxième fois selon ce que j'ai entendu.

MR 101 case vide

MR 102 dans le cas de l'apparition des taches et des cicatrices sur le visage, la personne sent la gêne et la honte et va essayer d'utiliser un mouchoir pour les cachés des autres.

+MR 103 case vide

+MR 104 Puisque j'ai été touché par cette maladie j'avais très peur qu'il apparaisse d'une manière plus grande sur mon corps et qu'il soit transmis à ma famille.

*MR 105 La peur d'affronter la société à cause des multiples cicatrices qui défigurent l'apparence extérieure.

MR 106 C’est une maladie très grave qui apparait à cause des multiples déchets existants et il influence la peau touchée et laisse des cicatrices.

MR 107 C’est une maladie qui se transmet par le sang et le contact direct et c’est une maladie très mauvaise et j’ai très peur d’être touché également

MR 108 Les cicatrices conduisent à des malformations dans l’apparence de la personne comme le visage et les mains. Et ceci va influencer négativement la société. Cela peut aller jusqu’à affecter l’état psychologique probable de la personne touchée par ces cicatrices. C’est pour cette raison qu’il faut combattre tout ce qui peut être la cause de ces cicatrices

MR 109 case vide

MR 110 Il peut influencer la personne touchée négativement, car les effets de cette maladie sont trop grands. La personne sent la honte devant ses amis à cause des cicatrices.

MR 111 Case vide

MR 112 Selon mon point de vue, il faut trouver un traitement pour cette maladie, car la personne touchée gardera les cicatrices le restant de sa vie.

MR 113 la peur de la mort

+MR 114 Il peut être exposé à la mort lente

MR 115 Il se peut qu’elle sente un peu de faiblesse dans sa personnalité. Mais avec le temps il commence à oublier tout cela même avec la permanence de la cicatrice sur sa peau.

+MR 116 L’état psychologique de la personne touchée peut être influencé après le traitement car le problème est que les cicatrices ne disparaissent jamais.

+MR 117 il est inquiet et perturbé à cause de cette maladie car cette dernière influence son état psychologique et laisse des traces sur sa peau.

*MR 118 il faut que toute personne touchée par cette maladie d’être patient et prier Dieu de l’aider. Et de visiter le médecin régulièrement et suivre ce que dit le médecin. Pour les effets psychologiques

que peut avoir le malade est de ne pas pouvoir affronter sa société et ne pas être capable de sortir et il va s'isoler totalement à cause de sa honte due aux cicatrices sur sa peau.

* MR 119 oui ces cicatrices influencent la santé psychique de la personne touchée et qui doit être prudent pour se prévenir de cette maladie.

MR 120 le sentiment de la solitude et l'absence de la stabilité dans la vie quotidienne à cause du regard négatif de la société vers patient.

*MR 121 quand la maladie de la leishmaniose touche la personne elle doit aller voir le médecin, car c'est une maladie grave et qui influence les nerfs et l'immunité va s'effondrer. Egalement il cause chez le patient des effets psychologiques et dermatologiques pendant une durée très longue ce qui nécessite la visite d'un psychiatre.

+MR 122 case vide

MR 123 Cette maladie influence souvent quelques personnes car elle laisse depuis le début des cicatrices qui conduisent la personne jusqu'au suicide

MR 124 le remède est le Coran en plus la personne peut utiliser TAMAGHOT qui se trouve à Merzouga.

*MR 125 Il ne doit pas sentir la peine car pour toute maladie il y a un remède soit maintenant soit dans le futur.

+MR 126 case vide

+MR 127 l'état de la personne touchée est la peur sur sa santé, car il croit que cette maladie peut le tuer, et sa famille également a peur. Il existe des malformations sur la personne touchée ce qui le met dans une situation inconfortable et de honte.

MR 128 case vide

+MR 129 Lors de cette époque l'état psychologique de la personne touchée est douloureux car la maladie ne disparait pas qu'après de longues années.

MR 130 oui la personne ne peut exprimer aux autres ce qu'il sent à cause de sa maladie

+MR 131 Je ne sais pas mais la personne quand elle est touchée par la leishmaniose il perd le sommeil et devient inquiet et sa mère a peur pour lui.

MR 132 oui car la personne touchée ne peut pas dire ce qu'elle a aux autres personnes par peur qu'ils s'éloignent de lui.

*MR 133 case vide

*MR 134 L'état psychologique de la personne touchée par cette maladie est lié aux cicatrices et aux autres maladies graves

*MR 135 Oui

MR 136 case vide

+MR 137 case vide

MR 138 La personne touché par cette maladie sent la peur à cause des cicatrices qui se localisent sur le visage ce qui lui fait perdre un peu de sa beauté

*MR 139 il peut avoir un trouble psychologique s'il est touché par ces cicatrices au niveau de son visage ou quelque chose de semblable

MR 140 lors de l'atteinte vous allez sentir une grande douleur au niveau de la zone touché et vous aurez l'apparition d'une cicatrice …

+MR 141 dans la période où la personne est touché par cette maladie il sent la peur ce qui influence son psychique.

MR 142 on retient que les rats et les moustiques sont l'origine de l'atteinte de la personne par la leishmaniose cutanée.

MR 143 case vide

MR 144 cette maladie ne donne aucun effet sur la psychologie de la personne touchée, selon mon point de vue

MR 145 il faut faire attention des moustiques car ils sont à l'origine de l'apparition de cette grave maladie et aussi faire attention des rats.

MR 146 Dans le cas de l'atteinte par la leishmaniose il se peut que l'état psychologique soit influencé de la personne touchée et ceci apparait dans la peur et les troubles psychiques

+MR 147 en ce qui concerne l'état psychologique probable est qu'il n'y a aucun effet sur l'état psychologique même après que les cicatrices restent à cause de cette maladie car il n'y a pas de grandes peurs

+MR 148 on peut dire que la maladie de la leishmaniose peut être guérie mais après traitement il reste les cicatrices pour toujours et il faut acheter un traitement "cicatrisol*"

MR 149 dans le cas de la présence des cicatrices sur le visage de la personne touché il y aura bien évidement un effet psychologique et la personne va sentir la honte.

*MR 150 cette personne touchée n'a pas le pouvoir de dire qu'il est atteint par cette maladie par peur que les gens s'éloignent de lui.

MR 151 si la personne est touché au niveau du visage, il se peut que la maladie affecte son état psychique avec l'apparition de la honte de montrer son visage et surtout devant les filles si la personne touchée est de sexe masculin et il va sentir également une laideur ce qui influence son état psychologique négativement.

+MR 152 pour toute maladie il y a un traitement

+MR 153 la personne touchée est toujours entrain de se demander pourquoi je suis celui qui a eu cette maladie et je porte cette marque sur le visage. Et comme vous le savez la société et la rue ne sont pas clémentes.

+MR 154 case vide

+MR 155 état psychologique tout à fait normal

+MR 156 case vide

MR 157 il faut se munir de la patience

+MR 158 la personne touché est dans un état embarrassant car sa peau est défigurée et ne reste pas normale et j'aimerai bien qu'il y est un traitement pour cette maladie qui ne laisse pas de cicatrices éternelles.

MR 159 lors de l'atteinte par cette maladie on va sentir la douleur et la peine à cause de cette maladie s'il y a des solutions possibles pour l'éviter cela serait agréable.

+MR 160 la personne touchée ne peut montrer cette atteinte à ses amis car ils ne vont pas accepter de s'assoir avec lui s'ils se rendent compte qu'il est touché par la leishmaniose.

+MR 161 case vide

MR 162 Refus de participer

MR 163 Refus de participer

MR 164 Refus de participer

MR 165 Refus de participer

MR 166 case vide

MR 167 peut être la sensation d'être différent par rapport à ses amis qui l'entoure à cause des taches et des cicatrices sur le corps dans les parties atteintes. Ce qui va le faire sentir un complexe d'infériorité et peut être qu'il sera exposé aux moqueries par les autres

MR 168 selon mon point de vue il n'y a aucun effet sur l'état psychologique de la personne puisqu'il y a un traitement même en cas de présence de cicatrices. Et il n'y aura aucune peur sur le malade en cas de traitement.

MR 169 il se peut que la personne touché a peur que son visage se défigure ou il y aura la persistance de la cicatrice sur son corps ou la peur que cela dure.

MR 170 case vide

MR 171 case vide

MR 172 je n'étais pas touché moi-même par cette maladie, mais mon ami l'avait, il n'était absolument pas en paix dans sa tête ni dans sa santé à cause de ce danger. Et c'est pour cela qu'il faut vaincre une telle maladie.

+MR 173 la personne touché déteste les cicatrices sur son visage surtout et il reste inquiet et son état psychologique et sentimental sont influencés car il se dit cette maladie n'a pas de traitement.

MR 174 il faut soigner rapidement cette maladie pour qu'il n'y ai pas d'effet sur l'état psychologique de la personne touchée

MR 175 cette maladie ne donne aucun effet sur l'état psychologique car ce n'est pas une chose essentielle. On est habitué à voir cette maladie. Et il faut que le gouvernement trouve une solution à cette maladie.

FT176 on peut dire que l'état psychologique de la personne touché par ces cicatrices est un très mauvais état et surtout chez les filles car cela conduit à la diminution de leurs beautés.

+FT177 quand la personne voit les cicatrices il aura un état psychologique lamentable surtout il va plonger dans la souffrance quand il se rend compte qu'il n'y a pas de traitement.

+FT178 on peut dire que l'état psychologique de la personne touché par ces cicatrices est trop difficile et surtout chez les filles car cela va faire diminuer leurs beautés

FT179 on peut dire que la personne touché par cette maladie souffre de cette dernière dans tous les temps, par exemple vous ne voulez pas qu'une autre personne soit touchée par cette maladie car elle fait souffrir beaucoup.

FT180 son état psychologique est affecté un peu car il a peur de contaminer ses proches et sa famille

+FT181 l'état psychologique de la personne touché par ces cicatrices est mauvais car il conduit à l'infériorité chez le porteur de ces cicatrices.

FT182 la réponse sera que la personne ne va absolument pas supporter cela car il conduit à une perte de performance dans les études et à la démotivation.

FT183 l'état psychologique probable de la personne touché par ces cicatrices sera dangereux ou bien cela ne va absolument pas l'affecté

*FT184 je prie Dieu qu'il guéri toute personne touché par cette maladie. Amen.

FT185 il se peut que l'aspect des cicatrices et les taches de la leishmaniose cutanée qu'elles influencent l'état psychologique et surtout celui des filles. Car elles vont sentir l'infériorité et la peur qu'elles apparaissent sur cet aspect devant leurs amis. Elles vont s'auto-détester quand elles vont se voir dans le miroir à cause des cicatrices et des taches de cette maladie.

FT186 il se peut que l'apparence des cicatrices et des taches de la leishmaniose cutanée qu'elles influencent l'état psychologique de la personne touchée. Elle va se sentir trop faible pour paraitre devant les amies avec ces cicatrices et surtout si ces taches sont sur le visage. Car la personne pense que les gens sont entrain de jeter un regard de méprise. La personne touchée va se dégouter en se voyant dans le miroir.

FT187 au niveau du visage et surtout chez certaines filles, les cicatrices peuvent représenter un problème psychologique à cause de l'aspect visuel horrible et la longue période que cela prend pour commencer à disparaitre de la peau.

FT188 l'état psychologique probable de la personne touchée par ces cicatrices peut être les troubles psychologiques

FT189 il se peut que l'apparence visuelle des cicatrices et des taches de la leishmaniose cutanée, influence l'état psychologique de la personne touchée dans le cas de la localisation au niveau du visage.

FT190 si ces cicatrices se situent dans une zone intérieure il n'y a pas d'influence sur l'état psychologique mais si elles se trouvent dans une zone extérieure visible comme au niveau du visage, il y aura un effet sur l'état psychologique.

FT191 si ces cicatrices se trouvent dans une place qu'on peut couvrir et cacher par les vêtements, comme au niveau du dos, son effet sur la psychologie de la personne touchée sera de moindre intensité. Mais si ces cicatrices se trouvent dans une place qu'on ne pourra pas caché comme le visage il y aura un effet très négatif sur l'état psychologique de la personne.

FT192 il se peut que l'état psychologique de la personne devienne un peu effondré, et il se peut qu'il sente la honte de paraitre en public avec ses cicatrices. Ces derniers seront un obstacle pour lui.

FT193 Pour éviter la leishmaniose cutanée il faut lutter contre les ordures et les éliminer.

FT194 surtout chez les filles quand la maladie laisse une tache sur le visage par exemple, et la fille va penser que c'est dangereux sur son niveau de beauté ce qui va influencer son état psychologique. Surtout dans notre société traditionnelle qui n'est absolument pas clémente à l'égard de ceux qui ont des taches sur le visage car ils pensent que c'est héréditaire.

+FT195 oui les cicatrices de la leishmaniose vont laisser chez l'homme un état psychologique très grave. Et surtout chez les filles touchées au niveau du visage. Les cicatrices te rendent très honteux devant les autres. Y a-t-il un traitement pour les cicatrices ? J'aimerai bien que la réponse sera oui il y a un traitement.

*FT196 oui cette maladie de la leishmaniose influence l'état psychologique de la personne touchée car lors de l'atteinte par cette maladie tu vas sentir par exemple que les gens sont entrain de s'éloigner de vous et de vous éviter. Et quand la personne touchée par cette maladie voit les cicatrices sur sa propre peau au niveau des mains et du visage elle a un sentiment de peur et de dépression. Peur que cette maladie l'influence plus et devienne mortelle.

*FT197 case vide

*FT198 je demande à Dieu qu'il guéri toute personne touchée par cette maladie dans les plus brèves délais et qu'il épargne ma famille et tous les musulmans

+FT199 pour moi je ne souffre d'aucun problème psychologique à cause de cette maladie même si j'ai une cicatrice mais je n'ai pas souffert à cause d'elle.

FT200 case vide

+FT201 si apparait les taches et les cicatrices de la leishmaniose cutanée au niveau de son visage, il aura très peur de faire face à cette réalité. Il se peut que pour les filles de ne pas pouvoir se marier car leurs visages se sont défiguré et cela va créer des effets psychologiques dangereux. J'espère que vous allez vaincre cette maladie.

+FT202 selon mon point de vue la fille touchée est plus affectée à cause de cette maladie et surtout s'elle est touchée au niveau du visage. Elle aura peur sur son avenir et sur ses rêves et surtout peur pour le mariage. Alors que le garçon touché dans notre société il reste un homme et il n'y a aucun mal s'il a des cicatrices.

+FT203 pour ma part j'étais touchée par cette maladie et je n'ai souffert d'aucun problème psychique

FT204 je pense que cette maladie n'influence pas l'état psychologique de la personne touchée. Et de façon générale elle n'a pas d'effet selon mon point de vue.

FT205 ces cicatrices influencent la personne touchée au niveau du visage ou les mains et créé d'autres choses ; il est souhaitable de consulter un médecin.

FT206 l'état psychologique de la personne touchée par cette maladie est un peu mauvais car il sent que cette maladie va continuer dans le temps et tout cela à cause des moustiques

FT207 selon ce que j'ai pu constater auprès des personnes touchées que je connaisse, est qu'elles sont influencées par ces cicatrices. Surtout chez les filles qui ont une atteinte au niveau du visage ce qui leurs fait perdre leurs beautés. Et cela les poussent à s'isoler et de ne vouloir entendre aucune remarque ou rumeur sur sa beauté.

FT208 je prie pour la personne touchée par ces cicatrices qu'elle guérisse. Qu'elle utilise des traitements médicaux ou traditionnelles c'est pareil pour moi, il faut juste qu'il guérisse et de ne pas développer un problème psychologique.

FT209 Je prie Dieu qu'il guérisse vite les personnes touchées par ces cicatrices et qu'il éloigne de nous cette maladie de la leishmaniose cutanée.

FT210 il doit suivre quelques injections et les conseils du médecin pour diminuer l'effet de ces cicatrices

FT211 selon mon point de vue, il se peut qu'il y soit une influence sur l'état psychologique de la personne touchée. Mais cela dépend de l'évaluation donnée par l'infirmier et l'ampleur de la dangerosité de la maladie.

FT212 selon ce que j'ai vu et j'ai entendu chez la plupart des filles touchées par cette maladie, elles sentent qu'elles ont une malédiction et ces cicatrices font obstacle devant leurs beautés.

FT213 Oui il est possible que les cicatrices influencent l'état psychologique de la personne touchée et surtout si la maladie guérie et laisse des séquelles sur le visage plus précisément.

FT214 Son état psychologique sera très affecté du fait que les cicatrices vont rester sur la peau surtout au niveau du visage des filles

FT215 je peux dire que l'état psychologique peut être affecté chez les personnes touchées par cette maladie. Ces taches et cicatrices peuvent représenter un obstacle pour elles. Si les malformations se situent sur les parties extérieures visibles, ces personnes vont sentir de l'infériorité et un déséquilibre dans leurs beautés ce qui va menacer l'état psychologique.

FT216 l'état psychologique de la personne touchée se manifeste par une sensation d'infériorité dans la vie et même la sensation de la perte du gout de la vie car il se croit être diffèrent des gens.

FT217 l'état psychologique de la personne touchée par ces cicatrices est de sentir le dégout parce que les gens l'évitent et qu'il ne va pas trouver de traitement pour ces cicatrices.

FT218 ces cicatrices vont influencer d'une grande manière l'état psychologique de la personne touchée surtout si elles sont situées sur les parties extérieures visibles comme le visage et les mains. La personne a honte de sortir devant les gens. Il y a de nombreux cas qui restent dans la maison à cause de ces cicatrices car elles créent une sorte d'embarrât chez a personne touchée.

FT219 ces cicatrices influencent beaucoup l'état psychologique de la personne touchée qui créent une honte qu'elle s'affiche comme personne affectée par cette maladie

FT220 l'état psychologique de la personne touché par ces cicatrices est de sentir l'éloignement de ses amies et ses proches de lui.

FT221 selon mon point de vue, ce genre de maladie influence négativement la santé des personnes. Car il laisse des taches au niveau de la peau et de là il peut se transmettre d'une personne à une autre. C'est pour cela qu'il faut le combattre par tous les moyens.

FT222 il se peut que l'apparence visuelle des cicatrices de la leishmaniose cutanée influence l'état psychologique de la personne touchée. Car ces cicatrices ne disparaissent pas au niveau de la peau et surtout s'elles sont localisées sur le visage.

FT223 les cicatrices influencent l'état psychologique en le rendant très déprimé. Et influencent aussi sur les sentiments en installant la honte de se montrer.

FT224 la personne touchée par cette maladie qui s'appelle leishmania sera affecté psychologiquement et cela peut laisser apparaitre parfois une dépression. Ou quelque chose de semblable surtout si la personne touchée n'a pas les moyens financiers pour se faire soigner de ces cicatrices.

FT225 la personne touché est trop inquiète et n'est pas tranquille à cause de cette atteinte et le sentiment de la honte qui l'accompagne en présence des gens. Il s'auto interdit d'aller dans les rassemblements à cause de cette maladie.

*FT226 l'état psychologique de la personne se manifeste par l'irritation et la facilité de s'énerver.

FT227 il se peut que l'état psychologique de la personne touchée l'emmène à se détester soi-même et de se mépriser. Comme le fait qu'il ne va plus avoir envie en rien et surtout la fille quand elle est touchée au niveau du visage ceci est très embarrassant. J'espère que vous allez être à la hauteur de nos attentes pour se débarrasser de cette maladie.

FT228 il se peut que ces cicatrices influencent l'état psychologique dans le cas où la maladie persiste ou en cas de dérangement causé par ces cicatrices. Et surtout si elles apparaissent de manière très grande et très visible sur le corps et particulièrement sur le visage.

FT229 il se peut que l'état psychologique de la personne touchée par ces cicatrices soit effondré et qu'elle soit suivie par un sentiment d'infériorité, de faiblesse et de dépression.

FT230 l'état psychologique probable de la fille touchée est plus accentué que par rapport à celui du garçon. Les cicatrices au niveau du visage diminuent la beauté des femmes et notre société marocaine n'est pas avérée ni consciente.

FT231 chez certaines filles touches et surtout quand elles le sont au niveau du visage, elles vont utiliser plusieurs méthodes non convenables pour se débarrasser de ces cicatrices. Ce qui est en général faux car elles pensent que si ces cicatrices restent sur leurs visages elles ne vont pas pouvoir se marier ce qui aggrave leurs états psychologiques

FT232 Oui il devient très irrité et il ne cesse de gratter, il s'énerve et perd le sommeil et il sent la solitude

FT233 la personne touchée est inquiète par cette atteinte et il évite d'aller aux réunions de familles et d'amies car il sent de la honte devant les gens.

FT234 la leishmaniose est une maladie dermatologique chronique. Il se peut qu'il soit irrité et avoir honte avec une grande peur

FT235 case vide

FT236 on souhaite la guérison rapide de tout patient qui souffre de cette maladie, grâce à Dieu.

FT237 la leishmaniose est une maladie dermatologique qui est d'apparition récente. Cette maladie entraine la souffrance des habitants du Maroc et surtout les populations rurales où se trouvent les forêts. Et ceci est causé par les rats et les moustiques et les insectes et aussi par l'eau et les animaux. Il y a des solutions proposées de la part des médecins si la personne touchée va vite chercher un traitement. Sinon il va avoir une dépression et une peur permanente et la honte et il ne va absolument pas avoir le courage de s'assoir avec ces amis par peur de leurs moqueries.

FT238 La personne touché devient anxieuse et doit lire ce dicton « Dieu de tous les gens emporte cette malheur et guéri moi, vous êtes le guérisseur qui donne la guérison absolue »

FT239 cette maladie influence la psychologie des filles car les cicatrices affectent leurs beautés et comme on le sait la société marocaine est non miséricordieuse

FT240 case vide

FT241 oui je suis d'accord avec les personnes qui vivent cet état psychologique et surtout les filles. Car notre société n'est pas miséricordieuse et juge selon l'apparence extérieure de la personne.

FT242 l'état psychologique peut être très affectée car la maladie peut être incorporée dans la peau et va se transmettre à travers le grattage. Ce qui pousse la personne à devenir plus irritable et cela pèse sur son psychisme comme le sentiment d'être malheureux. Et on demande Dieu la guérison aux personnes touchées.

FT243 selon mon point de vue, l'état psychologique de la personne touchée par ces cicatrices peut être influencé comme s'il ne va pas sortir à l'extérieur pour ne pas être vu par les gens. Il deviendra (introverti) fermé sur soi-même et il ne va plus parler avec les gens et il ne va plus aimer se voir lui-même à cause de ces cicatrices.

FT244 selon mon point de vue la leishmaniose cutanée peut influencer l'état psychologique de la personne touchée par les cicatrices de cette maladie. Il se peut que l'Homme ne veuille plus être vu par les autres gens et il ne va plus supporter la présence de ces cicatrices.

FT245 case vide.

FT246 case vide.

FT247 case vide.

FT248 l'apparence des cicatrices et des lésions de la leishmaniose cutanée influencent l'état psychologique de la personne touchée car il se produit chez lui des perturbations comme la peur et l'anxiété et le sentiment de l'infériorité et la gêne.

FT249 Il sent l'infériorité.

+FT250 Dans la plus part des cas il se peut que l'apparence des cicatrices et des taches de la leishmaniose influencent l'état psychologique de personnes touchées. Comme par exemple chez les filles il se peut que la fille sente la honte et la timidité dans le cas d'une atteinte au niveau de son visage surtout.

FT251 case vide.

FT252 il est possible que l'apparence des cicatrices et des taches de la leishmaniose cutanée d'influencer l'état psychologique de la personne touchée.

FT253 Il faut consulter un médecin pour faire diminuer ces cicatrices. Car elles laissent des taches sur la peau.

FT254 Il est possible que la personne touchée par les cicatrices dans ce cas de développer un état psychologique non normal. Et il faut le traiter le plus rapidement possible.

FT255 on peut voir dans notre vie quotidienne quelques personnes qui ont été influencé par ces cicatrices ce qui crée chez elles un sentiment de peur des dangers de ces cicatrices et ceci va les conduire à des maladies psychologiques comme la dépression.

FT256 il faut que tous les gens prennent soin de leurs santés et l'entretiennent et il faut éviter tout ce qui peut lui créer des dégâts comme les ordures. Et dès qu'on voit un moustique on doit le chasser pour éviter cette maladie.

FT257 il se peut que son état soit mal et lamentable car la personne est touchée par cette maladie. Car elle crée des effets négatifs sur la santé et aussi l'absence de satisfaction sur la défiguration de son image ce qui influence plus son état psychologique.

FT258 case vide

FT259 on peut dire que l'état psychologique de la personne touché par ces cicatrices est un état très normal et que ces cicatrices n'exercent aucun effet sur sa vie.

FT260 il se peut que cette maladie apparaisse chez l'Homme sur sa peau et reste pendant une durée déterminée mais si elle ne la soigne pas cela peut l'amener à une situation dangereuse.

FT261 la maladie de la leishmaniose cutanée est considérée dangereuse et peut entrainer la mort de la personne.

FT262 évidement les séquelles liées à la leishmaniose cutanée influencent négativement l'état de la personne touché car il se développe un complexe psychologique et a honte de paraitre avec ces cicatrices devant ses amis car c'est une marque de honte et de méprise.

+FT263 c'est évident que les effets causes par la leishmaniose influencent négativement l'état psychologique de la personne touché, avec le développement d'un complexe psychologique et a honte de paraitre devant ses amis.

FT264 la personne touchée par ces cicatrices est probable qu'elle a un état psychologique perturbé à cause de son sentiment permanent d'isolement et d'anxiété par rapport aux autres, ce qui conduit obligatoirement vers la dépression.

+FT265 tout à fait, les séquelles laissées par la leishmaniose influencent négativement l'état de la personne touchée. Il y aura un complexe psychologique et elle va avoir honte de paraitre devant ses amis car c'est une marque de honte et de méprise.

FT266 on souhaite la guérison de cette maladie et que Dieu avec sa puissance le guérisse.

FT267 l'état psychologique de la personne touchée par ces cicatrices peut être très critique et la personne peut sentir la timidité et la honte de sortir en dehors de la maison pendant cette maladie contagieuse par peur que ses amis n'aillent pas lui tenir compagnie.

FT268 case vide

FT269 case vide

FT270 oui il se peut que l'état psychologique se détériore chez la personne touché par cette maladie à cause de plusieurs raisons : sa peur des gens de le voir avec cet apparence non correct et surtout chez les filles. C'est une maladie grave.

FT271 la personne quand elle est touché par cette maladie sens le Malheur et surtout si les cicatrices sont sur le visage. Chaque fois qu'elle se regarde au miroir il sent l'infériorité et peut être il sera affecté par des crises psychologiques chroniques qui nécessiteront un suivi chez le spécialiste.

FT272 quand la personne est touché son état psychologique s’aggrave et cela par la peur de ce que vont penser les autres, surtout pour la fille.

FT273 selon la psychologie et la personnalité de la personne touchée et le degré de sa foi, celui qui a une faiblesse dans ces fondements et donnait plus de valeur à la beauté et aime se voir il est possible qu’il va être touché par la folie à cause des cicatrices. Mais celui qui a une foi forte et une psychologie saine et hygiénique il n’y aura aucune peur pour lui.

*FT274 case vide

*FT275 case vide

*FT276 case vide

FT277 l’état psychologique peut se détériorer à cause de cette maladie dangereuse. Et dans certains cas il se peut qu’on n’aille pas sortir de la maison par peur que les gens le voient.

FT278 je pense qu’il y a plusieurs méthodes pour lutter contre cette maladie contagieuse pour stopper sa transmission d’une personne à une autre. Il faut visiter les médecins et surtout si on est une femme touchée par cette maladie.

FT279 je peux dire que la femme est la plus à risque d’être touchée par cette maladie car elle supporte trop pour sauver sa famille et se sacrifie pour eux.

FT280 il y a plusieurs effets psychologiques que les personnes touchés en souffrent à cause de cette maladie. Comme l’évitement des autres personnes en préférant l’isolement du milieu extérieur du faite des nombreuses critiques qu’on lui formule à propos de cette maladie.

FT281 la personne touché par cette maladie souffre de troubles psychiques et développe un complexe psychologique en évitant les gens en optant pour la solitude et il commence à douter en soi-même et perd son auto-confiance.

FT282 Dans le cas de l’atteinte par cette maladie, la personne touché peut avoir un état psychologique affectée qui ne connait pas la stabilité et sentira l’infériorité.

FT283 toujours la personne sent la peur et la honte surtout si la maladie est localisée sur le visage. Il va penser qu'il est isolé et qu'il va sentir l'anxiété.

+FT284 on vous demande de pouvoir éviter cette maladie, car plusieurs personnes n'avaient pas les moyens pour acheter un traitement pour cette maladie dangereuse. Et je souhaite la guérison à tous ceux qui en souffrent.

FT285 quand la personne est touchée par cette maladie il doit se protéger de plusieurs autres maladies qui seront causées par cette première maladie.

FT286 quand la personne est touchée par cette maladie il voit qu'il est devenu défiguré dans son corps à cause de la présence de ces marques cicatricielles sur sa peau, ce qui lui pousse à sentir la honte et la panique. Et je suis pour la réalisation d'une compagne de sensibilisation contre cette maladie.

FT287 la leishmaniose cutanée influence l'état psychologique de la personne touchée car elle lui pousse à avoir peur et même à la mort. C'est pour cela qu'il faut se protéger et protéger nos familles. Et en cas d'apparition de cette maladie il faut aller voir le médecin spécialiste.

FT288 il est probable que la personne touchée par cette maladie a un état psychologique perturbé.

FT289 la leishmaniose cutanée influence l'état psychologique et conduit à la mort. Et pour diminuer l'effet de cette maladie contagieuse il faut prévoir assez de traitements médicaux ou traditionnels.

FT290 la maladie de la leishmaniose est un phénomène dangereux et très répondu surtout dans les villes. Et cette maladie influence l'état psychologique de la personne touchée car elle conduit à la peur. Il faut réaliser des compagnes de sensibilisation sur l'intérêt de l'hygiène. Et on souhaite la guérison aux personnes atteintes.

FT291 il est probable que la personne touchée par cette maladie a un état psychologique perturbé.

FT292 l'état psychologique de la personne touché est marqué par le fait qu'il devient paniqué avec une grande peur, et il n'aime plus rencontrer les gens car ils commencent à l'éviter et ceci le rend triste.

FT293 quand la personne est énervée ou ignorante elle peut avoir des problèmes psychologiques, on demande à Dieu le pardon et la santé.

FT294 la leishmaniose cutanée influence l'état psychologique et conduit à la peur. La peur même de la mort. D'où il faut organiser des compagnes de sensibilisation sur l'importance de l'hygiène pour lutter contre les rats sauvages et les moustiques. Et il faut qu'on se protège de la leishmaniose cutanée et protéger nos familles. Et je souhaite la guérison rapide aux personnes touchées par cette maladie.

FT295 la personne touchée aura un état psychologique très effondré à cause du milieu où il vit et le regard de la société à son égard d'autre part. Il va sentir la dépression et il ne va plus vouloir sortir avec les amis et les proches et il va s'auto-emprisonner dans la maison.

MT296 point d'exclamation.

MT297 la dépression, la peur de perdre sa beauté surtout les filles et ceci va influencer négativement son état psychologique comme la peur de ne pas pouvoir se marier.

MT298 cette maladie laisse des cicatrices sur la peau et ceci pousse la personne à prendre conscience des changements de son corps et cela va être la cause d'une influence psychologique.

*MT299 je pense que cette personne touchée va avoir une perturbation dans sa vie quotidienne.

MT300 ils doivent visiter le médecin le plus proche car cette maladie est très dangereuse et je leurs souhaite une guérison rapide.

*MT301 la personne touchée pense que son apparence visuelle est différent des autres ce qui va lui créer des perturbations psychiques.

MT302 l'état psychologique probable de la personne touchée par cette maladie est négative, car il se dit je suis touché et les autres vont s'éloigner de moi pour qu'ils ne soient pas contaminés et ceci va engendrer une influence sur l'état psychologique de cette personne qui va vivre en solitude.

MT303 la personne porteuse de ces cicatrices, a honte des gens qui sont sains et qui ne portent pas de cicatrices. Et il sera delà en colère quand il sentira que les cicatrices ne diminuent pas avec le temps. Son psychisme va s'affaiblir un peu ou beaucoup selon le degré de sa croyance en Dieu.

+MT304 case vide

MT305 on peut dire qu'il va vivre sous l'effet du poids psychologique causé par le regard de la société à l'égard de la personne touchée par cette maladie. Cela va mener à l'isolement social et elle va vivre à l'écart des autres par peur de les contaminer.

MT306 case vide

+MT307 l'état psychologique de la personne touchée par cette maladie par ces cicatrices est en relation seulement avec la place de localisation de ces cicatrices. S'elles se trouvent au niveau du visage ce n'est pas comme s'elles se trouvent au niveau des mains … etc. la personne touchée souffre plus si la cicatrice est au niveau du visage.

MT308 case vide

MT309 je pense que l'état psychologique de la personne touchée par ces cicatrices sera marqué par la honte.

*MT310 l'isolement du monde extérieur et la perte de confiance en soi.

MT311 Il se peut que l'aspect des cicatrices influence l'état psychologique de la personne touchée. Car cette personne n'a plus la même apparence que les autres. Delà il va sentir qu'il est définitivement différent des autres ce qui lui pousse à l'isolement, à la solitude et à d'autres maladies psychiatriques.

MT312 On peut dire que la personne touchée par cette maladie aura des problèmes sur son état psychique et physique.

MT313 Le pardon

MT314 je ne peux pas expliquer cela car je ne connais pas bien cette maladie.

*MT315 il se peut ou non que la personne touchée sente la honte.

MT316 case vide

*MT317 case vide

*MT318 case vide

*MT319 case vide

MT320 case vide

*MT321 case vide

MT322 case vide

MT323 case vide

MT324 son état psychologique sera trop dangereux

MT325 case vide

MT326 il se peut que les cicatrices l'influence psychologiquement ou lui cause la mort

MT327 case vide

MT328 Si cette maladie me touche, il se peut que j'aie peur et je serai inquiet sur ma personne

MT329 il se peut qu'il influence la personne touchée par ces cicatrices qui restent sur son corps et qui défigurent sa beauté.

+MT330 Quand j'étais touché par cette maladie, j'ai eu l'apparition de marques et j'avais très peur

MT331 case vide

MT332 quand la personne est touchée par cette maladie, elle reste cachée car la plupart des gens évitent de lui parler. La personne touchée a envie d'enlever ces cicatrices par toute méthode possible ou les faire cacher.

MT333 il se peut que la personne touchée par ces cicatrices qu'il soit touché par une maladie psychiatrique car les séquelles de la leishmaniose peuvent exposées les personnes à une maladie psychiatrique

MT334 la personne touchée dès la première fois doit informer le médecin rapidement avant la multiplication des celulles.

MT335 je vais écrire sur l'état psychologique de la personne touchée par la maladie de la leishmaniose, cette personne peut douter en soi parce que les gens sont entrain de parler à propos de lui. J'espère que tout le monde sera sérieux pour l'élimination de cette maladie et le virus qui en est responsable.

MT336 la visite du médecin et la lutte contre les rats et les moustiques et le ramassage des ordures dans des endroits bien spécifiques.

MT337 en regard que la maladie de la leishmaniose cutanée est difficilement guérissable et laisse des cicatrices et des taches permanentes sur le corps et qui influencent l'apparence normale connue de la personne. Ainsi cette maladie provoque beaucoup de problèmes psychologiques car elle diminue la beauté de son apparence extérieure et cause l'irritation chez lui car la société n'aperçoit que les défauts et pense que la personne touchée est une personne négligente qui est non propre et qui ne prend pas soins de son état corporel.

+MT338 on peut dire que la lutte contre cette maladie nécessite l'utilisation des insecticides et les compagnes de chirurgie esthétique.

MT339 il se peut que ces cicatrices causent de nombreuses maladies psychologiques comme la dépression

MT340 il se peut que ces cicatrices influencent l'état psychologique des personnes touchées par cette maladie et conduit à des perturbations psychologiques multiples dans la vie quotidienne et également sentimentale.

*MT341 case vide

MT342 il faut que chaque personne utilise les insecticides contre les insectes et les rats qui transmettent cette maladie très grave. Et j'espère qu'il ne va toucher personne et merci.

MT343 case vide

MT344 ces cicatrices qui paraissent sur le corps et surtout la peau à des effets sur le corps comme la création d'autres maladies. D'autre part sur l'état psychologique selon mon point de vue elle ne donne aucun effet sauf si sa taille grandisse et conduit à la mort de la personne surtout pour le petit enfant et les vieux, mais pour les jeunes femmes elles peuvent supporter.

+MT345 Cette maladie peut toucher la personne s'il est fragile.

MT346 la personne touchée souffre de cette maladie à cause de sa peur de l'éventualité de mourir à cause de cette maladie. J'ai peur de cette maladie

MT347 il faut que la personne touchée parte chez le médecin pour qu'il le soigne s'il a un traitement. Et je prie Dieu qu'il guérisse toute personne de cette maladie. Et merci de s'intéresser à ce genre de maladie.

MT348 on vous demande une solution pour qu'on puisse éviter cette maladie qui nous fait peur par ces cicatrices qui restent.

+MT349 il faut compter sur Dieu. Comme il dit au Coran : « je me met à la volonté de Dieu »

*MT350 il se peut que cette maladie influence l'état de santé en premier et puis sur l'état psychologique à cause des cicatrices et les défigurements qui en résultent et qui sont considérée comme un handicap ou une honte dans les sociétés où on vit. Il faut prendre ces points en considération et renforcer les efforts pour lutter contre cette maladie et Merci beaucoup.

+MT351 souvent la personne touchée est dans un état psychologique normal même en étant touché par cette maladie.

MT352 l'apparition de signes caractéristiques de cette maladie par les troubles psychologiques et l'isolement de la société par peur de se moquer de lui surtout de la part de son entourage.

+MT353 case vide

MT354 case vide

MT355 pour éviter cette maladie et l'épidémie dangereuse il faut que les responsables assurent leurs rôles et que la population y participe.

MT356 la fuite de la société de la personne touchée par peur que la maladie contamine d'autres personnes.

MT357 case vide

MT358 la personne touchée va souffrir et va avoir une crise psychologique surtout si les cicatrices de cette maladie restent sur le visage par exemple.

MT359 l'impuissance de dormir et ne pas cesser de penser à sa maladie. Et la peur d'être touché par cette dernière.

MT360 il est probable qu'il sera atteint par un peu de peur et de honte devant ses amis. Et il ne va pas pouvoir partager les repas avec sa famille

*MT361 Je peux dire que cette maladie n'est pas fréquente dans la société et il y a plusieurs sociétés où cette maladie n'existe pas car il n'y a pas la présence de certains animaux porteurs de cette maladie et qui le transmettent à l'humain.

MT362 Il se peut que les cicatrices l'influencent si elles sont présentes dans des zones sensibles et découvertes et qu'elles ne guérissent pas par les traitements médicaux modernes. On demande à Dieu le pardon et la santé.

MT363 l'état psychologique de cette personne est un peu difficile. Mais il se peut qu'il y ait une personne qui peut développer des troubles psychologiques et une autre qui reste normale.

+MT364 la personne touchée par cette maladie peut souffrir s'elle est touchée dans une zone sensible comme au niveau du visage ce qui le rend embarrassé devant les amis et les collègues et la société en gros ce qui le pousse à se ralentir dans ses études et son travail.

MT365 il a peur de sortir de la maison à cause de cette maladie car s'il sort il va transmettre la maladie à ses amis

MT366 il se peut que la personne perde son état psychologique au sein de sa famille.

MT367 il se peut que cette maladie apparaissent à cause des moustiques et cause des démangeaisons qui laissent des cicatrices.

MT368 on est prêt à collaborer avec les médecins pour nous proposer une méthode pour éliminer cette maladie et stopper sa transmission.

MT369 je n'ai absolument aucune idée sur l'état psychologique de la personne touchée par cette maladie.

MT370 je ne peux pas expliquer l'état psychologique de la personne touchée par cette maladie. Ceci est trop difficile, car chacun a sa propre psychologie.

MT371 case vide

MT372 Je ne sais pas

MT373 pour la personne touchée par cette maladie il souffrira d'une crise psychologique si la maladie lui laisse des cicatrices sur la peau du visage par exemple.

MT374 il se peut que la personne ait des problèmes psychologiques en cas de la perte de son immunité acquise.

MT375 Il se peut que la personne touchée par cette maladie ne supporte pas les taches sur la surface de sa peau. Car ces dernières vont faire fuir les personnes indemnes par peur d'avoir eux aussi cette maladie. Ainsi il ne va pas supporter cela et va souffrir psychologiquement.

MT376 la vie devient dure à cause de l'état psychologique de la personne touchée. Les cicatrices influencent sur sa vie psychologique. Heureusement que cette maladie a commencé à diminuer par rapport auparavant.

MT377 je n'ai absolument aucune idée

+MT378 par exemple le sentiment de la honte devant ses amis. Et la peur sur la beauté de son visage.

MT379 la personne aura honte d'affronter les gens et aussi du fait des défigurations qui touchent son visage à cause de cette maladie.

MT380 case vide

MT381 je prie Dieu qu'il le guérisse. Si dans sa région se trouve les rats, il doit mener une compagne pour les éliminer. Et j'espère que tout le monde s'unit contre cette maladie.

MT382 il se peut que la personne touchée soit affectée à cause de la perte de l'immunité

MT383 je pense que l'état psychologique de la personne touchée tend vers la dépression et la perturbation. Cela à cause de deux facteurs principaux : la peur qu'il affecte l'un des membres de son entourage et la peur de la peur de cette maladie elle-même.

MT384 pour la personne touchée par cette maladie son état psychologique devient faible et il va sentir une infériorité importante ce qui amène à une maladie psychiatrique qui peut l'accompagner dans sa vie estudiantine et dans sa vie sociale d'une façon générale.

MT385 Je lui souhaite la guérison rapide

MT386 la personne touchée va avoir un doute en soi-même. Et les cicatrices vont l'influencer et le poussent à voir un médecin pour guérir son état psychologique et peut être cela va l'aider pour retrouver un peu de sa santé. Mais s'il est dans un état psychologique normal ce n'est pas un problème d'avoir ces cicatrices.

+MT387 case vide

MT388 Il aura peur pour soi-même et inquiet. Il ne saura pas ce qu'il doit faire ce qui va le rendre très irrité.

MT389 Pour l'état psychologique de la personne touchée, cela dépendra de la personne elle-même. Soit qu'il va supporter cela ou il ne va pas le supporter. S'il ne va pas le supporter du fait que cette maladie se trouve dans une partie considérée très importante pour sa mobilité, cela va lui poser un complexe psychologique.

MT390 case vide

MT391 case vide

MT392 il n'y aura aucun effet sur l'état psychologique et vive le Roi.

MT393 Refus de participer

MT394 Refus de participer

MT395 lors de l'atteinte par cette maladie il faut se rendre à l'hôpital pour faire la vaccination. Car cette maladie est très dangereuse et laisse place à des cicatrices sur les zones touchées de la peau. Et vive le Roi.

MT396 Pour la femme, ces cicatrices va lui créer un obstacle avec ces amies et son travail et d'autres choses comme ça.

*MT397 Je souhaite aux personnes touchées par cette maladie la guérison rapide. Il faut faire des compagnes de propreté et de lutte contre les rats sauvages (de la forêt). J'espère que je ne serai pas touché ni moi ni les autres par cette maladie qui est dangereuse et qui provoque beaucoup d'autres maladies.

*MT398 je pense que cette maladie est très forte et elle fait beaucoup de problèmes dans le monde. Je veux dire tout simplement que je veux souhaiter à l'homme qui a cette maladie (leishmaniose) la grande santé et merci à tous.

*MT399 case vide

*MT400 la personne a peur des remarques des gens. Ce qui va l'emmener à rester dans la maison et sentir la solitude. Il faut condenser les compagnes dans les zones ignorantes.

MT401 case vide

MT402 la personne touchée par cette maladie aura un état psychologique très perturbé.

MT403 la personne touchée doit laver ces cicatrices par le savon et il doit éliminer les moustiques et bien nettoyer.

MT404 l'aspect de ces cicatrices peut influencer la psychologie de la personne touchée par cette maladie. Il se peut qu'il aille sentir un peu d'inquiétude et de non satisfaction s'il a cette maladie sur le visage.

MT405 autant qu'élève j'ai vu de telles situations ou phénomène. J'oriente tous les gens pour adopter la propreté quotidienne et lutter contre tous les insectes et surtout les rats et les moustiques. Et cela par l'utilisation des insecticides.

*MT406 il doit chercher le traitement dans les meilleurs délais possible pour ne pas trop souffrir de cette maladie. Et on demande à Dieu la guérison rapide.

MT407 Même si vous n'avez fourni aucun effort pour lutter contre cette maladie. On remercie Dieu de nous avoir épargné nous et nos familles qui sont restées indemnes. Et on prie Dieu la guérison rapide.

MT408 il se peut que la personne touchée par ces cicatrices sente l'infériorité ou qu'il sente que son apparence extérieure est défigurée et perturbée.

MT409 son état psychologique sera perturbé à cause des malformations sur le visage et le sentiment d'isolement et d'infériorité

MT410 on demande à Dieu la guérison rapide. Il faut se guérir par certaines herbes du Sahara.

MT411 je ne peux dire qu'il faut aller à une consultation médicale dans les plus brefs délais. Et on prie Dieu la guérison et le traitement.

MT412 il faut que la personne touchée se munisse de la patience et ne pas s'intéresser à ces cicatrices car elles se trouvent souvent dans toutes les places.

MT413 Oui si un parmi les jeunes personnes est touché par cette maladie au niveau du visage, il sera incapable d'affronter la société par peur de la discrimination sociale et la méprise et par peur d'autres facteurs qui rendent sa psychologie épuisée.

MT414 je ne pense pas pouvoir connaitre l'état psychologique de la personne touchée par ces cicatrices

MT415 Je peux écrire dans une petite phrase à propos de l'état psychologique probable chez la personne touchée par ces cicatrices. Il peut guérir ce fléau par les médicaments

MT416 il se peut qu'il y ait une jeune personne touchée par cette maladie et donc il peut avoir un problème psychologique

MT417 case vide

MT418 Aucune information, je souhaite la guérison et ne jamais être touché.

MT419 la cicatrice n'est pas le problème, le problème c'est vous (GOV) vous avez rien à faire, vous êtes des « f »

*MT420 Je ne sais pas

MT421 toute personne touchée par cette maladie a l'apparition de boutons rouges et il devient malade et il va gratter la zone de l'atteinte et la place gratter va devenir visible et va grandir et laisser une cicatrice sur la peau atteinte.

MT422 je pense que la leishmaniose cutanée et ses cicatrices influencent l'état psychologique de la personne et cela du fait de sa capacité de répugner son apparence en se comparant aux autres. Et ceci va conduire à choisir des voies positives ou négatives comme l'utilisation des médicaments traditionnels et de la chirurgie.

MT423 Je ne sais rien

MT424 Oui il se peut qu'il y ait la peur de cette maladie

MT425 bien évidement il y aura un peu de peur de ce phénomène et un peu de prudence pour se protéger est le meilleur traitement.

MT426 Je connais quelqu'un qui est touché par cette maladie depuis la cinquième année du primaire mais elle n'a pas fait d'effet sur lui. Les cicatrices ont disparu après un mois seulement. Il a utilisé des traitements traditionnels comme j'ai cité plus haut (le savon traditionnel, le sel et l'eau de rose).

MT427 Souvent les filles sont touchées et souffrent de la leishmaniose cutanée et surtout lors de son atteinte sur le visage. Ceci peut la faire perdre sa beauté.

*MT428 case vide

MT429 je ne considère pas la leishmaniose cutanée comme maladie dangereuse, mais je considère les autres maladies mentales qui influencent la vie de l'être humain comme tel.

MT430 La maladie mentale est la plus dangereuse, elle influence négativement la vie de la personne.

*MT431 je souhaite la guérison à toute personne touchée par cette maladie. Il faut éliminer les rats sauvages et lutter contre les insectes pour faire disparaitre cette maladie.

MT432 case vide

MT433 Il se peut que ces cicatrices poussent les malades à avoir des troubles psychologiques ; tout en sachant que les cicatrices ne disparaissent pas et la personne peut revivre le même état si les taches restent dessiner sur sa peau.

*MT434 il va continuer à visiter le médecin pour cette maladie et il va s'isoler de sa famille et de la société autant que possible

*MT435 la personne touchée développe un complexe psychologique et il va éviter de sortir à l'extérieur car son visage est tout couvert par des grandes taches.

+MT436 La personne touchée a beaucoup peur qu'il contamine les membres de sa famille et il ne va pas s'assoir souvent auprès d'eux. Il va avoir peur que cette maladie lui provoque d'autres maladies dans le futur.

MT437 quand la personne est touchée par cette maladie dangereuse, il aura très peur, ce qui va le pousser à penser uniquement à sa maladie. Et delà il y aura une grande influence sur son état psychologique.

MT438 il va sentir un peu d'isolement

+MT439 si l'atteinte est sur une zone découverte comme le visage et la main, et que l'atteinte devient plus grande avec absence de traitements médicaux modernes, cela peut entrainer une dépression psychique interne principalement car il n'aime pas que les autres voient ces cicatrices.

MT440 ces cicatrices peuvent laisser la personne dans une situation d'isolement des autres personnes. Surtout si les cicatrices sont sur le visage et peuvent être visibles par les autres. Donc il aura une dépression.

+MT441 il se peut qu'il sentira une gêne et une honte quand il va sortir en dehors de la maison surtout si la tache de la leishmaniose se trouve sur le visage et est très apparente.

MT442 je ne sais pas. Dieu seul sait. Chaque maladie a un traitement. Et la guérison vient de Dieu.

MT443 l'état psychologique est lamentable et surtout les filles. Car les filles sont les plus intéressées par leurs beautés et apparences.

MT444 ces cicatrices peuvent conduire à l'incapacité de communiquer et de s'intégrer avec les autres, car elle laisse un grand effet sur les zones cutanées sensibles surtout au niveau du visage.

MT445 case vide

*MT446 je ne sais pas

MT447 je lui souhaite la guérison et qu'il utilise ce qu'il peut pour éviter cette maladie

MT448 Il va avoir un complexe psychologique surtout s'il est un adolescent, car il va rester malheureux pour son état de corporel. Alors que son corps est l'aimant attractif des filles.

MT449 des troubles psychologiques et l'absence de sentiment de paix et la haine de soi-même et surtout s'il est un adolescent comme moi.

MT450 la personne touchée aura un dérangement psychologique car il a peur de contaminer et de transmettre cette maladie aux autres membres de sa famille

*MT451 la personne touchée va chercher le médicament idéal et les médecins doivent chercher les moyens pour combattre cette maladie

+MT452 l'état psychologique est marquée par la complexité psychologique car il n'y a pas de traitement pour ces cicatrices et ils vont rester pour toujours. Et il voudra juste comment faire disparaitre ces cicatrices.

MT453 case vide

*MT454 moi je ne connais pas cette maladie pour pouvoir parler à propos de cela. Je m'excuse d'être ignorant pour cette maladie. « Tout ce que je sais est que je ne sais rien ».
