# Supplementary material for: Advancing AI-driven thematic analysis in qualitative research: a comparative study of nine generative models on Cutaneous Leishmaniasis data
Source: BMC Med Inform Decis Mak. 2025 Mar 10;25:124. doi: 10.1186/s12911-025-02961-5 (PMC11895178; doi:10.1186/s12911-025-02961-5)
Supplement: Supplementary file 4 — Supplementary Material 4: Additional file 1ter. Phase1A Kappa Cohen R calculation 31 12 2024 [file 12911_2025_2961_MOESM4_ESM.pdf]

R code used in Jamovi software

```
library(psych)

calculate_kappa <- function(var1, var2, label) {
  kappa_result <- cohen.kappa(cbind(var1, var2))
  print(paste("Kappa entre", label))
  print(kappa_result)
}

calculate_kappa(data$ManA_1st, data$ManA_2nd, "ManA_1st et ManA_2nd")
calculate_kappa(data$Ref_A, data$ManA_1st, "Ref_A et ManA_1st")
calculate_kappa(data$Ref_A, data$ManA_2nd, "Ref_A et ManA_2nd")

calculate_kappa(data$ClaudeSonnet_1st, data$ClaudeSonnet_2nd, "Claude_1st et Claude_2nd")
calculate_kappa(data$Ref_A, data$ClaudeSonnet_1st, "Ref_A et ClaudeSonnet_1st")
calculate_kappa(data$Ref_A, data$ClaudeSonnet_2nd, "Ref_A et ClaudeSonnet_2nd")

calculate_kappa(data$Gemini1.5_1st, data$Gemini1.5_2nd, "Gemini1.5_1st et Gemini1.5_2nd")
calculate_kappa(data$Ref_A, data$Gemini1.5_1st, "Ref_A et Gemini1.5_1st")
calculate_kappa(data$Ref_A, data$Gemini1.5_2nd, "Ref_A et Gemini1.5_2nd")

calculate_kappa(data$NoteboookLM_1st, data$NoteboookLM_2nd, "NoteboookLM_1st et
NoteboookLM_2nd")
calculate_kappa(data$Ref_A, data$NoteboookLM_1st, "Ref_A et NoteboookLM_1st")
calculate_kappa(data$Ref_A, data$NoteboookLM_2nd, "Ref_A et NoteboookLM_2nd")

calculate_kappa(data$LlaMA_1st, data$LlaMA_2nd, "LlaMA_1st et LlaMA_2nd")
calculate_kappa(data$Ref_A, data$LlaMA_1st, "Ref_A et LlaMA_1st")
calculate_kappa(data$Ref_A, data$LlaMA_2nd, "Ref_A et LlaMA_2nd")
```

```
calculate_kappa(data$`ChatGPT-o1_1st`, data$`ChatGPT-o1_2nd`, "ChatGPT_o1_1st et ChatGPT-o1_2nd")

calculate_kappa(data$Ref_A, data$`ChatGPT-o1_1st`, "Ref_A et ChatGPT_o1_1st")

calculate_kappa(data$Ref_A, data$`ChatGPT-o1_2nd`, "Ref_A et ChatGPT_o1_2nd")


calculate_kappa(data$`ChatGPT-o1PRO_1st`, data$`ChatGPT-o1PRO_2nd`, "ChatGPT_o1PRO_1st et ChatGPT_o1PRO_2nd")

calculate_kappa(data$Ref_A, data$`ChatGPT-o1PRO_1st`, "Ref_A et ChatGPT_o1PRO_1st")

calculate_kappa(data$Ref_A, data$`ChatGPT-o1PRO_2nd`, "Ref_A et ChatGPT_o1PRO_2nd")


calculate_kappa(data$GrokV2_1st, data$GrokV2_2nd, "GrokV2_1st et GrokV2_2nd")

calculate_kappa(data$Ref_A, data$GrokV2_1st, "Ref_A et GrokV2_1st")

calculate_kappa(data$Ref_A, data$GrokV2_2nd, "Ref_A et GrokV2_2nd")


calculate_kappa(data$DeepSeekV3_1st, data$DeepSeekV3_2nd, "DeepSeekV3_1st et DeepSeekV3_2nd")

calculate_kappa(data$Ref_A, data$DeepSeekV3_1st, "Ref_A et DeepSeekV3_1st")

calculate_kappa(data$Ref_A, data$DeepSeekV3_2nd, "Ref_A et DeepSeekV3_2nd")


calculate_kappa(data$Gemini2.0_1st, data$Gemini2.0_2nd, "Gemini2.0_1st et Gemini2.0_2nd")

calculate_kappa(data$Ref_A, data$Gemini2.0_1st, "Ref_A et Gemini2.0_1st")

calculate_kappa(data$Ref_A, data$Gemini2.0_2nd, "Ref_A et Gemini2.0_2nd"
```

Response

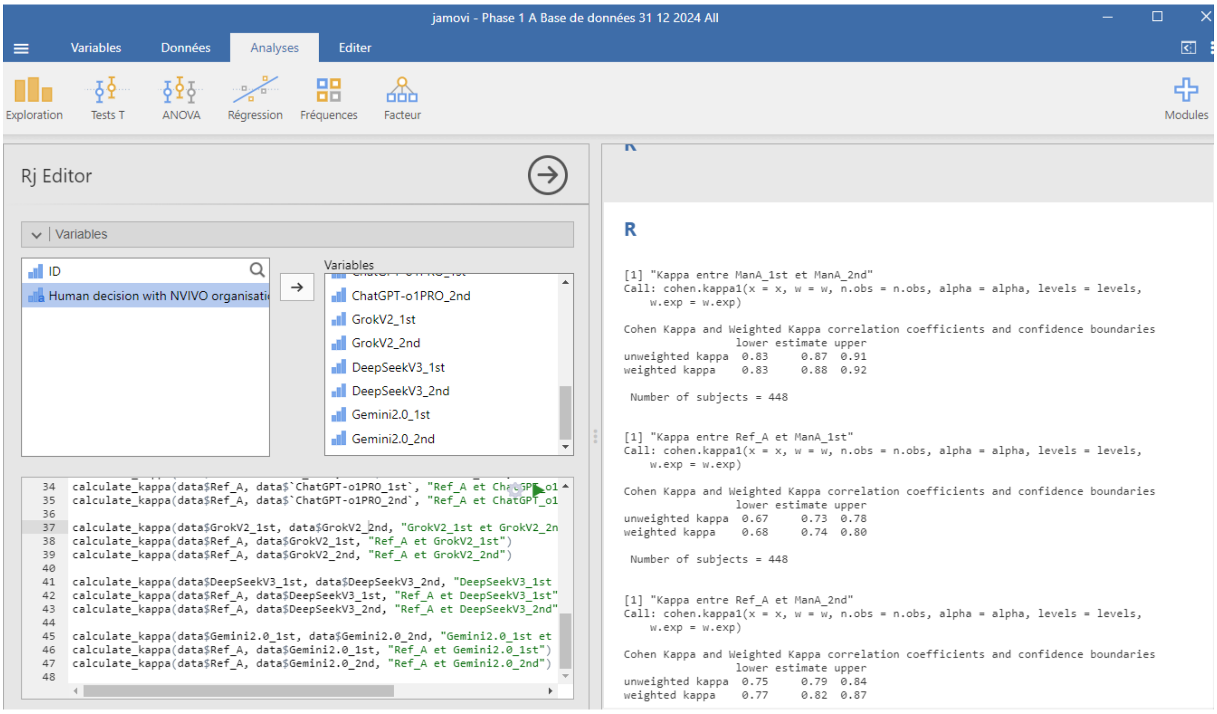

Results

R

```
[1] "Kappa entre ManA_1st et ManA_2nd"
Call: cohen.kappa1(x = x, w = w, n.obs = n.obs, alpha = alpha, levels =
levels,
    w.exp = w.exp)

Cohen Kappa and Weighted Kappa correlation coefficients and confidence
boundaries
                 lower estimate upper
unweighted kappa  0.83     0.87  0.91
weighted kappa    0.83     0.88  0.92

 Number of subjects = 448
[1] "Kappa entre Ref_A et ManA_1st"
Call: cohen.kappa1(x = x, w = w, n.obs = n.obs, alpha = alpha, levels =
levels,
    w.exp = w.exp)

Cohen Kappa and Weighted Kappa correlation coefficients and confidence
boundaries
                 lower estimate upper
unweighted kappa  0.67     0.73  0.78
```

```
weighted kappa    0.68     0.74  0.80

 Number of subjects = 448
[1] "Kappa entre Ref_A et ManA_2nd"
Call: cohen.kappa1(x = x, w = w, n.obs = n.obs, alpha = alpha, levels =
levels,
    w.exp = w.exp)

Cohen Kappa and Weighted Kappa correlation coefficients and confidence
boundaries
                 lower estimate upper
unweighted kappa  0.75     0.79  0.84
weighted kappa    0.77     0.82  0.87

 Number of subjects = 448
[1] "Kappa entre Claude_1st et Claude_2nd"
Call: cohen.kappa1(x = x, w = w, n.obs = n.obs, alpha = alpha, levels =
levels,
    w.exp = w.exp)

Cohen Kappa and Weighted Kappa correlation coefficients and confidence
boundaries
                 lower estimate upper
unweighted kappa  0.96     0.98     1
weighted kappa    0.97     0.99     1

 Number of subjects = 448
[1] "Kappa entre Ref_A et ClaudeSonnet_1st"
Call: cohen.kappa1(x = x, w = w, n.obs = n.obs, alpha = alpha, levels =
levels,
    w.exp = w.exp)

Cohen Kappa and Weighted Kappa correlation coefficients and confidence
boundaries
                 lower estimate upper
unweighted kappa  0.73     0.78  0.83
weighted kappa    0.73     0.78  0.84

 Number of subjects = 448
[1] "Kappa entre Ref_A et ClaudeSonnet_2nd"
Call: cohen.kappa1(x = x, w = w, n.obs = n.obs, alpha = alpha, levels =
levels,
    w.exp = w.exp)

Cohen Kappa and Weighted Kappa correlation coefficients and confidence
boundaries
                 lower estimate upper
unweighted kappa  0.73     0.78  0.82
weighted kappa    0.73     0.78  0.84

 Number of subjects = 448
[1] "Kappa entre Gemini1.5_1st et Gemini1.5_2nd"
Call: cohen.kappa1(x = x, w = w, n.obs = n.obs, alpha = alpha, levels =
levels,
    w.exp = w.exp)

Cohen Kappa and Weighted Kappa correlation coefficients and confidence
boundaries
                 lower estimate upper
unweighted kappa  0.85     0.89  0.93
weighted kappa    0.89     0.92  0.96
```

```
 Number of subjects = 448
[1] "Kappa entre Ref_A et Gemini1.5_1st"
Call: cohen.kappa1(x = x, w = w, n.obs = n.obs, alpha = alpha, levels =
levels,
    w.exp = w.exp)

Cohen Kappa and Weighted Kappa correlation coefficients and confidence
boundaries
                 lower estimate upper
unweighted kappa  0.68     0.73  0.78
weighted kappa    0.67     0.73  0.79

 Number of subjects = 448
[1] "Kappa entre Ref_A et Gemini1.5_2nd"
Call: cohen.kappa1(x = x, w = w, n.obs = n.obs, alpha = alpha, levels =
levels,
    w.exp = w.exp)

Cohen Kappa and Weighted Kappa correlation coefficients and confidence
boundaries
                 lower estimate upper
unweighted kappa  0.71     0.76  0.81
weighted kappa    0.72     0.77  0.83

 Number of subjects = 448
[1] "Kappa entre NoteboookLM_1st et NoteboookLM_2nd"
Call: cohen.kappa1(x = x, w = w, n.obs = n.obs, alpha = alpha, levels =
levels,
    w.exp = w.exp)

Cohen Kappa and Weighted Kappa correlation coefficients and confidence
boundaries
                 lower estimate upper
unweighted kappa  0.87     0.91  0.94
weighted kappa    0.89     0.93  0.96

 Number of subjects = 448
[1] "Kappa entre Ref_A et NoteboookLM_1st"
Call: cohen.kappa1(x = x, w = w, n.obs = n.obs, alpha = alpha, levels =
levels,
    w.exp = w.exp)

Cohen Kappa and Weighted Kappa correlation coefficients and confidence
boundaries
                 lower estimate upper
unweighted kappa  0.66     0.71  0.77
weighted kappa    0.65     0.72  0.78

 Number of subjects = 448
[1] "Kappa entre Ref_A et NoteboookLM_2nd"
Call: cohen.kappa1(x = x, w = w, n.obs = n.obs, alpha = alpha, levels =
levels,
    w.exp = w.exp)

Cohen Kappa and Weighted Kappa correlation coefficients and confidence
boundaries
                 lower estimate upper
unweighted kappa  0.70     0.75  0.80
weighted kappa    0.71     0.76  0.82
```

```
 Number of subjects = 448
[1] "Kappa entre LlaMA_1st et LlaMA_2nd"
Call: cohen.kappa1(x = x, w = w, n.obs = n.obs, alpha = alpha, levels =
levels,
    w.exp = w.exp)

Cohen Kappa and Weighted Kappa correlation coefficients and confidence
boundaries
                 lower estimate upper
unweighted kappa  0.81     0.85  0.89
weighted kappa    0.73     0.79  0.86

 Number of subjects = 448
[1] "Kappa entre Ref_A et LlaMA_1st"
Call: cohen.kappa1(x = x, w = w, n.obs = n.obs, alpha = alpha, levels =
levels,
    w.exp = w.exp)

Cohen Kappa and Weighted Kappa correlation coefficients and confidence
boundaries
                 lower estimate upper
unweighted kappa  0.75     0.79  0.84
weighted kappa    0.68     0.75  0.82

 Number of subjects = 448
[1] "Kappa entre Ref_A et LlaMA_2nd"
Call: cohen.kappa1(x = x, w = w, n.obs = n.obs, alpha = alpha, levels =
levels,
    w.exp = w.exp)

Cohen Kappa and Weighted Kappa correlation coefficients and confidence
boundaries
                 lower estimate upper
unweighted kappa  0.74     0.79  0.83
weighted kappa    0.72     0.78  0.83

 Number of subjects = 448
[1] "Kappa entre ChatGPT_o1_1st et ChatGPT-o1_2nd"
Call: cohen.kappa1(x = x, w = w, n.obs = n.obs, alpha = alpha, levels =
levels,
    w.exp = w.exp)

Cohen Kappa and Weighted Kappa correlation coefficients and confidence
boundaries
                 lower estimate upper
unweighted kappa  0.70     0.75  0.80
weighted kappa    0.75     0.80  0.85

 Number of subjects = 448
[1] "Kappa entre Ref_A et ChatGPT_o1_1st"
Call: cohen.kappa1(x = x, w = w, n.obs = n.obs, alpha = alpha, levels =
levels,
    w.exp = w.exp)

Cohen Kappa and Weighted Kappa correlation coefficients and confidence
boundaries
                 lower estimate upper
unweighted kappa  0.71     0.76  0.81
weighted kappa    0.71     0.77  0.82

 Number of subjects = 448
```

```
[1] "Kappa entre Ref_A et ChatGPT_o1_2nd"
Call: cohen.kappa1(x = x, w = w, n.obs = n.obs, alpha = alpha, levels =
levels,
    w.exp = w.exp)

Cohen Kappa and Weighted Kappa correlation coefficients and confidence
boundaries
                 lower estimate upper
unweighted kappa  0.60     0.65  0.71
weighted kappa    0.65     0.71  0.76

 Number of subjects = 448
[1] "Kappa entre ChatGPT_o1PRO_1st et ChatGPT_o1PRO_2nd"
Call: cohen.kappa1(x = x, w = w, n.obs = n.obs, alpha = alpha, levels =
levels,
    w.exp = w.exp)

Cohen Kappa and Weighted Kappa correlation coefficients and confidence
boundaries
                 lower estimate upper
unweighted kappa  0.94     0.97  0.99
weighted kappa    0.94     0.97  0.99

 Number of subjects = 448
[1] "Kappa entre Ref_A et ChatGPT_o1PRO_1st"
Call: cohen.kappa1(x = x, w = w, n.obs = n.obs, alpha = alpha, levels =
levels,
    w.exp = w.exp)

Cohen Kappa and Weighted Kappa correlation coefficients and confidence
boundaries
                 lower estimate upper
unweighted kappa  0.73     0.78  0.83
weighted kappa    0.74     0.79  0.85

 Number of subjects = 448
[1] "Kappa entre Ref_A et ChatGPT_o1PRO_2nd"
Call: cohen.kappa1(x = x, w = w, n.obs = n.obs, alpha = alpha, levels =
levels,
    w.exp = w.exp)

Cohen Kappa and Weighted Kappa correlation coefficients and confidence
boundaries
                 lower estimate upper
unweighted kappa  0.73     0.78  0.83
weighted kappa    0.73     0.79  0.84

 Number of subjects = 448
[1] "Kappa entre GrokV2_1st et GrokV2_2nd"
Call: cohen.kappa1(x = x, w = w, n.obs = n.obs, alpha = alpha, levels =
levels,
    w.exp = w.exp)

Cohen Kappa and Weighted Kappa correlation coefficients and confidence
boundaries
                 lower estimate upper
unweighted kappa  0.75     0.80  0.84
weighted kappa    0.72     0.78  0.84

 Number of subjects = 448
[1] "Kappa entre Ref_A et GrokV2_1st"
```

```
Call: cohen.kappa1(x = x, w = w, n.obs = n.obs, alpha = alpha, levels =
levels,
    w.exp = w.exp)

Cohen Kappa and Weighted Kappa correlation coefficients and confidence
boundaries
                 lower estimate upper
unweighted kappa  0.61     0.67  0.72
weighted kappa    0.60     0.66  0.73

 Number of subjects = 448
[1] "Kappa entre Ref_A et GrokV2_2nd"
Call: cohen.kappa1(x = x, w = w, n.obs = n.obs, alpha = alpha, levels =
levels,
    w.exp = w.exp)

Cohen Kappa and Weighted Kappa correlation coefficients and confidence
boundaries
                 lower estimate upper
unweighted kappa  0.71     0.76  0.81
weighted kappa    0.71     0.77  0.83

 Number of subjects = 448
[1] "Kappa entre DeepSeekV3_1st et DeepSeekV3_2nd"
Call: cohen.kappa1(x = x, w = w, n.obs = n.obs, alpha = alpha, levels =
levels,
    w.exp = w.exp)

Cohen Kappa and Weighted Kappa correlation coefficients and confidence
boundaries
                 lower estimate upper
unweighted kappa  0.87      0.9  0.94
weighted kappa    0.86      0.9  0.94

 Number of subjects = 448
[1] "Kappa entre Ref_A et DeepSeekV3_1st"
Call: cohen.kappa1(x = x, w = w, n.obs = n.obs, alpha = alpha, levels =
levels,
    w.exp = w.exp)

Cohen Kappa and Weighted Kappa correlation coefficients and confidence
boundaries
                 lower estimate upper
unweighted kappa   0.7     0.74  0.79
weighted kappa     0.7     0.76  0.81

 Number of subjects = 448
[1] "Kappa entre Ref_A et DeepSeekV3_2nd"
Call: cohen.kappa1(x = x, w = w, n.obs = n.obs, alpha = alpha, levels =
levels,
    w.exp = w.exp)

Cohen Kappa and Weighted Kappa correlation coefficients and confidence
boundaries
                 lower estimate upper
unweighted kappa  0.70     0.75  0.80
weighted kappa    0.69     0.75  0.81

 Number of subjects = 448
[1] "Kappa entre Gemini2.0_1st et Gemini2.0_2nd"
```

```
Call: cohen.kappa1(x = x, w = w, n.obs = n.obs, alpha = alpha, levels =
levels,
    w.exp = w.exp)

Cohen Kappa and Weighted Kappa correlation coefficients and confidence
boundaries
                 lower estimate upper
unweighted kappa  0.68     0.73  0.78
weighted kappa    0.74     0.79  0.85

 Number of subjects = 448
[1] "Kappa entre Ref_A et Gemini2.0_1st"
Call: cohen.kappa1(x = x, w = w, n.obs = n.obs, alpha = alpha, levels =
levels,
    w.exp = w.exp)

Cohen Kappa and Weighted Kappa correlation coefficients and confidence
boundaries
                 lower estimate upper
unweighted kappa  0.56     0.62  0.67
weighted kappa    0.57     0.63  0.69

 Number of subjects = 448
[1] "Kappa entre Ref_A et Gemini2.0_2nd"
Call: cohen.kappa1(x = x, w = w, n.obs = n.obs, alpha = alpha, levels =
levels,
    w.exp = w.exp)

Cohen Kappa and Weighted Kappa correlation coefficients and confidence
boundaries
                 lower estimate upper
unweighted kappa  0.68     0.73  0.78
weighted kappa    0.70     0.76  0.82

 Number of subjects = 448
```
