# Supplementary material for: Advancing AI-driven thematic analysis in qualitative research: a comparative study of nine generative models on Cutaneous Leishmaniasis data
Source: BMC Med Inform Decis Mak. 2025 Mar 10;25:124. doi: 10.1186/s12911-025-02961-5 (PMC11895178; doi:10.1186/s12911-025-02961-5)
Supplement: Supplementary file 8 — Supplementary Material 8: Additional file 3bis. Phase 1C 79 Students with CL PNU Jamovi results 31 12 2024 [file 12911_2025_2961_MOESM8_ESM.pdf]

Phase 1C Analysis of 79 students with CL coded P N U 31 12 2024

Résultats

Tables de contingence

Tables de contingence

	Ref_A			
**Gender**	**P**	**N**	**U**	**Total**
F	25	6	4	35
M	23	8	13	44
Total	48	14	17	79

Tests χ^2

	Valeur	p
**Test exact de Fisher**		0.135
**N**	79	

Tables de contingence

Tables de contingence

	ManA_1st			
**Gender**	**P**	**N**	**U**	**Total**
F	24	7	4	35
M	21	4	19	44
Total	45	11	23	79

Tests χ^2

	Valeur	p

**Test exact de Fisher**		0.00601
**N**	79	

Tables de contingence

Tables de contingence

	ManA_2nd			
**Gender**	**P**	**N**	**U**	**Total**
F	27	4	4	35
M	22	4	18	44
Total	49	8	22	79

Tests χ^2

	Valeur	p
**Test exact de Fisher**		0.0110
**N**	79	

Tables de contingence

Tables de contingence

	ClaudeSonnet_1st			
**Gender**	**P**	**N**	**U**	**Total**
F	26	4	5	35
M	21	5	18	44
Total	47	9	23	79

Tests χ^2

	Valeur	p
**Test exact de Fisher**		0.0269
**N**	79	

Tables de contingence

Tables de contingence

	ClaudeSonnet_2nd			
**Gender**	**P**	**N**	**U**	**Total**
F	26	4	5	35
M	21	4	19	44
Total	47	8	24	79

Tests χ^2

	Valeur	p
**Test exact de Fisher**		0.0190
**N**	79	

Tables de contingence

Tables de contingence

	NoteboookLM_1st			
**Gender**	**P**	**N**	**U**	**Total**
F	24	5	6	35
M	22	4	18	44
Total	46	9	24	79

Tests χ^2

	Valeur	p
**Test exact de Fisher**		0.0646
**N**	79	

Tables de contingence

Tables de contingence

	NoteboookLM_2nd			
**Gender**	**P**	**N**	**U**	**Total**
F	25	5	5	35
M	22	5	17	44
Total	47	10	22	79

Tests χ^2

	Valeur	ddl	p
χ^2	5.79	2	0.0554
**N**	79		

Tables de contingence

Tables de contingence

	Gemini1.5_1st			
**Gender**	**P**	**N**	**U**	**Total**
F	25	5	5	35
M	22	5	17	44
Total	47	10	22	79

Tests χ^2

	Valeur	ddl	p
χ^2	5.79	2	0.0554
**N**	79		

Tables de contingence

Tables de contingence

	Gemini1.5_2nd			

Gender	P	N	U	Total
F	24	6	5	35
M	23	4	17	44
Total	47	10	22	79

Tests χ^2

	Valeur	p
**Test exact de Fisher**		0.0433
**N**	79	

Tables de contingence

Tables de contingence

	LlaMA_1st			
**Gender**	**P**	**N**	**U**	**Total**
F	26	5	4	35
M	25	7	12	44
Total	51	12	16	79

Tests χ^2

	Valeur	p
**Test exact de Fisher**		0.182
**N**	79	

Tables de contingence

Tables de contingence

	LlaMA_2nd			
**Gender**	**P**	**N**	**U**	**Total**
F	26	5	4	35
M	23	9	12	44

Total	49	14	16	79

Tests χ^2

	Valeur	p
**Test exact de Fisher**		0.119
**N**	79	

Tables de contingence

Tables de contingence

	ChatGPT-o1_1st			
**Gender**	**P**	**N**	**U**	**Total**
F	26	4	5	35
M	22	3	19	44
Total	48	7	24	79

Tests χ^2

	Valeur	p
**Test exact de Fisher**		0.0182
**N**	79	

Tables de contingence

Tables de contingence

	ChatGPT-o1_2nd			
**Gender**	**P**	**N**	**U**	**Total**
F	25	5	5	35
M	19	10	15	44
Total	44	15	20	79

Tests χ^2

	Valeur	p
**Test exact de Fisher**		0.0433
**N**	79	

Tables de contingence

Tables de contingence

	ChatGPT-o1PRO_1st			
**Gender**	**P**	**N**	**U**	**Total**
F	26	4	5	35
M	23	3	18	44
Total	49	7	23	79

Tests χ^2

	Valeur	p
**Test exact de Fisher**		0.0318
**N**	79	

Tables de contingence

Tables de contingence

	ChatGPT-o1PRO_2nd			
**Gender**	**P**	**N**	**U**	**Total**
F	26	4	5	35
M	23	3	18	44
Total	49	7	23	79

Tests χ^2

	Valeur	p

**Test exact de Fisher**		0.0318
**N**	79	

Tables de contingence

Tables de contingence

	GrokV2_1st			
**Gender**	**P**	**N**	**U**	**Total**
F	24	6	5	35
M	23	6	15	44
Total	47	12	20	79

Tests χ^2

	Valeur	ddl	p
χ^2	4.05	2	0.132
**N**	79		

Tables de contingence

Tables de contingence

	GrokV2_2nd			
**Gender**	**P**	**N**	**U**	**Total**
F	26	4	5	35
M	24	4	16	44
Total	50	8	21	79

Tests χ^2

	Valeur	p
**Test exact de Fisher**		0.0820
**N**	79	

Tables de contingence

Tables de contingence

	DeepSeekV3_1st			
**Gender**	**P**	**N**	**U**	**Total**
F	25	5	5	35
M	23	4	17	44
Total	48	9	22	79

Tests χ^2

	Valeur	p
**Test exact de Fisher**		0.0525
**N**	79	

Tables de contingence

Tables de contingence

	DeepSeekV3_2nd			
**Gender**	**P**	**N**	**U**	**Total**
F	26	4	5	35
M	22	4	18	44
Total	48	8	23	79

Tests χ^2

	Valeur	p
**Test exact de Fisher**		0.0317
**N**	79	

Tables de contingence

Tables de contingence

	Gemini2.0_1st			
Gender	P	N	U	Total
F	25	4	6	35
M	18	7	19	44
Total	43	11	25	79

Tests χ^2

	Valeur	p
**Test exact de Fisher**		0.0188
**N**	79	

Tables de contingence

Tables de contingence

	Gemini2.0_2nd			
Gender	P	N	U	Total
F	26	4	5	35
M	22	6	16	44
Total	48	10	21	79

Tests χ^2

	Valeur	p
**Test exact de Fisher**		0.0728
**N**	79	

Références

[1] The jamovi project (2024). *jamovi*. (Version 2.6) [Computer Software]. Retrieved from https://www.jamovi.org.

[2] R Core Team (2024). *R: A Language and environment for statistical computing*. (Version 4.4) [Computer software]. Retrieved from https://cran.r-project.org. (R packages retrieved from CRAN snapshot 2024-08-07).
