# Supplementary material for: Advancing AI-driven thematic analysis in qualitative research: a comparative study of nine generative models on Cutaneous Leishmaniasis data
Source: BMC Med Inform Decis Mak. 2025 Mar 10;25:124. doi: 10.1186/s12911-025-02961-5 (PMC11895178; doi:10.1186/s12911-025-02961-5)
Supplement: Supplementary file 9 — Supplementary Material 9: Additional file 4bis. Phase 1C 63 Students with CL PNU Jamovi results 31 12 2024 [file 12911_2025_2961_MOESM9_ESM.pdf]

# Phase 1C Analysis of 63 students with CL coded P N U 31 12 2024

## Tables de contingence

Tables de contingence

| | Ref_A | | | |
|---|---|---|---|---|
| Gender | P | N | U | Total |
| F | 25 | 6 | 0 | 31 |
| M | 23 | 8 | 1 | 32 |
| Total | 48 | 14 | 1 | 63 |

Tests $\chi^2$

| | Valeur | p |
|---|---|---|
| **Test exact de Fisher** | | 0.658 |
| **N** | 63 | |

## Tables de contingence

Tables de contingence

| | ManA_1st | | | |
|---|---|---|---|---|
| Gender | P | N | U | Total |
| F | 24 | 7 | 0 | 31 |
| M | 21 | 4 | 7 | 32 |
| Total | 45 | 11 | 7 | 63 |

Tests $\chi^2$

| | Valeur | p |
|---|---|---|
| **Test exact de Fisher** | | 0.0158 |
| **N** | 63 | |

## Tables de contingence

Tables de contingence

| | ManA_2nd | | | |
|---|---|---|---|---|
| **Gender** | **P** | **N** | **U** | **Total** |
| F | 27 | 4 | 0 | 31 |
| M | 22 | 4 | 6 | 32 |
| Total | 49 | 8 | 6 | 63 |

Tests $\chi^2$

| | Valeur | p |
|---|---|---|
| **Test exact de Fisher** | | 0.0413 |
| **N** | 63 | |

## Tables de contingence

Tables de contingence

| | ClaudeSonnet_1st | | | |
|---|---|---|---|---|
| **Gender** | **P** | **N** | **U** | **Total** |
| F | 26 | 4 | 1 | 31 |
| M | 21 | 5 | 6 | 32 |
| Total | 47 | 9 | 7 | 63 |

Tests $\chi^2$

| | Valeur | p |
|---|---|---|
| **Test exact de Fisher** | | 0.127 |
| **N** | 63 | |

## Tables de contingence

Tables de contingence

| | ClaudeSonnet_2nd | | | |
|---|---|---|---|---|
| **Gender** | **P** | **N** | **U** | **Total** |
| F | 26 | 4 | 1 | 31 |
| M | 21 | 4 | 7 | 32 |
| Total | 47 | 8 | 8 | 63 |

Tests $\chi^2$

| | Valeur | p |
|---|---|---|
| **Test exact de Fisher** | | 0.109 |
| **N** | 63 | |

# Tables de contingence

Tables de contingence

| | NotebooookLM_1st | | | |
|---|---|---|---|---|
| **Gender** | **P** | **N** | **U** | **Total** |
| F | 24 | 5 | 2 | 31 |
| M | 22 | 4 | 6 | 32 |
| Total | 46 | 9 | 8 | 63 |

Tests $\chi^2$

| | Valeur | p |
|---|---|---|
| **Test exact de Fisher** | | 0.393 |
| **N** | 63 | |

# Tables de contingence

Tables de contingence

| | NotebooookLM_2nd | | | |
|---|---|---|---|---|
| **Gender** | **P** | **N** | **U** | **Total** |

| | | | | |
|---|---|---|---|---|
| F | 25 | 5 | 1 | 31 |
| M | 22 | 5 | 5 | 32 |
| Total | 47 | 10 | 6 | 63 |

Tests $\chi^2$

| | Valeur | p |
|---|---|---|
| **Test exact de Fisher** | | 0.280 |
| **N** | 63 | |

# Tables de contingence

Tables de contingence

| | **Gemini1.5_1st** | | | |
|---|---|---|---|---|
| **Gender** | **P** | **N** | **U** | **Total** |
| F | 25 | 5 | 1 | 31 |
| M | 22 | 5 | 5 | 32 |
| Total | 47 | 10 | 6 | 63 |

Tests $\chi^2$

| | Valeur | p |
|---|---|---|
| **Test exact de Fisher** | | 0.280 |
| **N** | 63 | |

# Tables de contingence

Tables de contingence

| | **Gemini1.5_2nd** | | | |
|---|---|---|---|---|
| **Gender** | **P** | **N** | **U** | **Total** |
| F | 24 | 6 | 1 | 31 |
| M | 23 | 4 | 5 | 32 |

| Total | 47 | 10 | 6 | 63 |
|---|---|---|---|---|

Tests $\chi^2$

| | Valeur | p |
|---|---|---|
| **Test exact de Fisher** | | 0.255 |
| **N** | 63 | |

# Tables de contingence

Tables de contingence

| | LlaMA_1st | | |
|---|---|---|---|
| **Gender** | **P** | **N** | **Total** |
| F | 26 | 5 | 31 |
| M | 25 | 7 | 32 |
| Total | 51 | 12 | 63 |

Tests $\chi^2$

| | Valeur | p |
|---|---|---|
| **Test exact de Fisher** | | 0.750 |
| **N** | 63 | |

# Tables de contingence

Tables de contingence

| | LlaMA_2nd | | |
|---|---|---|---|
| **Gender** | **P** | **N** | **Total** |
| F | 26 | 5 | 31 |
| M | 23 | 9 | 32 |
| Total | 49 | 14 | 63 |

Tests $\chi^2$

| | Valeur | p |
|---|---|---|
| **Test exact de Fisher** | | 0.365 |
| **N** | 63 | |

# Tables de contingence

Tables de contingence

| | ChatGPT-o1_1st | | | |
|---|---|---|---|---|
| **Gender** | **P** | **N** | **U** | **Total** |
| F | 26 | 4 | 1 | 31 |
| M | 22 | 3 | 7 | 32 |
| Total | 48 | 7 | 8 | 63 |

Tests $\chi^2$

| | Valeur | p |
|---|---|---|
| **Test exact de Fisher** | | 0.0903 |
| **N** | 63 | |

# Tables de contingence

Tables de contingence

| | ChatGPT-o1_2nd | | | |
|---|---|---|---|---|
| **Gender** | **P** | **N** | **U** | **Total** |
| F | 25 | 5 | 1 | 31 |
| M | 19 | 10 | 3 | 32 |
| Total | 44 | 15 | 4 | 63 |

Tests $\chi^2$

| | Valeur | p |
|---|---|---|

| | Valeur | p |
|---|---|---|
| **Test exact de Fisher** | | 0.210 |
| **N** | 63 | |

# Tables de contingence

Tables de contingence

| | ChatGPT-o1PRO_1st | | | |
|---|---|---|---|---|
| **Gender** | **P** | **N** | **U** | **Total** |
| F | 26 | 4 | 1 | 31 |
| M | 23 | 3 | 6 | 32 |
| Total | 49 | 7 | 7 | 63 |

Tests $\chi^2$

| | Valeur | p |
|---|---|---|
| **Test exact de Fisher** | | 0.197 |
| **N** | 63 | |

# Tables de contingence

Tables de contingence

| | ChatGPT-o1PRO_2nd | | | |
|---|---|---|---|---|
| **Gender** | **P** | **N** | **U** | **Total** |
| F | 26 | 4 | 1 | 31 |
| M | 23 | 3 | 6 | 32 |
| Total | 49 | 7 | 7 | 63 |

Tests $\chi^2$

| | Valeur | p |
|---|---|---|
| **Test exact de Fisher** | | 0.197 |
| **N** | 63 | |

# Tables de contingence

Tables de contingence

| | GrokV2_1st | | | |
|---|---|---|---|---|
| **Gender** | **P** | **N** | **U** | **Total** |
| F | 24 | 6 | 1 | 31 |
| M | 23 | 6 | 3 | 32 |
| Total | 47 | 12 | 4 | 63 |

Tests $\chi^2$

| | Valeur | p |
|---|---|---|
| **Test exact de Fisher** | | 0.742 |
| **N** | 63 | |

# Tables de contingence

Tables de contingence

| | GrokV2_2nd | | | |
|---|---|---|---|---|
| **Gender** | **P** | **N** | **U** | **Total** |
| F | 26 | 4 | 1 | 31 |
| M | 24 | 4 | 4 | 32 |
| Total | 50 | 8 | 5 | 63 |

Tests $\chi^2$

| | Valeur | p |
|---|---|---|
| **Test exact de Fisher** | | 0.519 |
| **N** | 63 | |

# Tables de contingence

Tables de contingence

| | DeepSeekV3_1st | | | |
|---|---|---|---|---|
| **Gender** | **P** | **N** | **U** | **Total** |
| F | 25 | 5 | 1 | 31 |
| M | 23 | 4 | 5 | 32 |
| Total | 48 | 9 | 6 | 63 |

Tests $\chi^2$

| | Valeur | p |
|---|---|---|
| **Test exact de Fisher** | | 0.313 |
| **N** | 63 | |

# Tables de contingence

Tables de contingence

| | DeepSeekV3_2nd | | | |
|---|---|---|---|---|
| **Gender** | **P** | **N** | **U** | **Total** |
| F | 26 | 4 | 1 | 31 |
| M | 22 | 4 | 6 | 32 |
| Total | 48 | 8 | 7 | 63 |

Tests $\chi^2$

| | Valeur | p |
|---|---|---|
| **Test exact de Fisher** | | 0.176 |
| **N** | 63 | |

# Tables de contingence

Tables de contingence

| | Gemini2.0_1st |
|---|---|

| Gender | P | N | U | Total |
|---|---|---|---|---|
| F | 25 | 4 | 2 | 31 |
| M | 18 | 7 | 7 | 32 |
| Total | 43 | 11 | 9 | 63 |

Tests $\chi^2$

| | Valeur | p |
|---|---|---|
| **Test exact de Fisher** | | 0.106 |
| **N** | 63 | |

## Tables de contingence

Tables de contingence

| | Gemini2.0_2nd | | | |
|---|---|---|---|---|
| **Gender** | **P** | **N** | **U** | **Total** |
| F | 26 | 4 | 1 | 31 |
| M | 22 | 6 | 4 | 32 |
| Total | 48 | 10 | 5 | 63 |

Tests $\chi^2$

| | Valeur | p |
|---|---|---|
| **Test exact de Fisher** | | 0.283 |
| **N** | 63 | |

## Références

**[1]** The jamovi project (2024). *jamovi*. (Version 2.6) [Computer Software]. Retrieved from https://www.jamovi.org.

**[2]** R Core Team (2024). *R: A Language and environment for statistical computing*. (Version 4.4) [Computer software]. Retrieved from https://cran.r-project.org. (R packages retrieved from CRAN snapshot 2024-08-07).
