# Supplementary material for: Advancing AI-driven thematic analysis in qualitative research: a comparative study of nine generative models on Cutaneous Leishmaniasis data
Source: BMC Med Inform Decis Mak. 2025 Mar 10;25:124. doi: 10.1186/s12911-025-02961-5 (PMC11895178; doi:10.1186/s12911-025-02961-5)
Supplement: Supplementary file 10 — Supplementary Material 10: Additional file 5. Prompts used in Phase 2‐1 and Phase 2‐2. Additional file 5bis. Phase 2‐1 Claude Sonnet 3.5 1st video demonstration. YouTube [32]. Additional file 5ter. Phase 2‐2 Gemini 2.0 Advanced 4th video demonstration. YouTube [33] [file 12911_2025_2961_MOESM10_ESM.pdf]

Prompt Phase 2 suivant Méthode 2-1 (Demande directe, sans apprentissage autour du contexte)

« Donner une synthèse qualitative suite à l'analyse globale des réponses suivantes des participants à propos de l'état psychologique probable de la personne portant la cicatrice de la leishmaniose cutanée.

Identifie et catégorise les thèmes et sous-thèmes présents dans chaque réponse. Ensuite, génère un rapport structuré présentant les thèmes et sous-thèmes principaux ainsi que leur fréquence d'occurrence. Enfin, calcule l'indice de Jaccard pour chaque thème par rapport aux données de référence fournies (sans les mentionner explicitement dans cette étape).

Instructions pour l'IA :

1. *Identifie les thèmes et sous-thèmes de l'ensemble de la base de données.*
2. *Présente un rapport structuré (de préférence sous forme de Matrice) avec les thèmes et sous-thèmes principaux ainsi que leur fréquence d'occurrence.*

***NB :** ces résultats seront comparés selon l'indice de Jaccard par rapport aux réponses types de référence. »*

Les 448 réponses des élèves sont les suivants : (Ajout en copier coller)

English translation:

Phase 2 Prompt Using Method 2-1 (Direct Request, Without Contextual Learning)

Instruction for AI in English:
"Provide a qualitative synthesis following the global analysis of the participants' responses concerning the probable psychological state of a person bearing a scar from cutaneous leishmaniasis.

1. Identify and categorize the themes and sub-themes present in each response.
2. Generate a structured report presenting the main themes and sub-themes along with their frequency of occurrence.
3. Finally, calculate the Jaccard index for each theme in relation to the provided reference data (without explicitly mentioning the reference data in this step).

Steps for the AI:

1. Identify themes and sub-themes from the entire database of responses.
2. Present a structured report (preferably in matrix form) including the main themes, sub-themes, and their frequency of occurrence.

Note: These results will be compared based on the Jaccard index against reference responses."

Participants' 448 responses are as follows: (Insert the responses via copy-paste)

<<<<<<<<<<<<<<<<<<<<<<<<<<<<<<<<<<<<<<<<<<<<<<<<<<<<<<<<<<<<<<<<<<<<<<<<<<<<<<

Prompt Phase 2 suivant Méthode 2-2 (Demande structurée basé sur un apprentissage autour du contexte et le suivi de la démarche d'analyse qualitative thématique)

L'analyse thématique est une méthode pour identifier, analyser et rapporter des motifs (thèmes) au sein des données. Voici les étapes typiques :

1. **Familiarisation** : Lire et relire les données pour se familiariser avec le contenu.
2. **Codage initial** : Identifier et coder les segments de texte pertinents pour la question de recherche.
3. **Recherche de thèmes** : Grouper les codes en thèmes potentiels.
4. **Révision des thèmes** : Vérifier et affiner les thèmes pour s'assurer qu'ils représentent bien les données.
5. **Définition et nomination des thèmes** : Définir et nommer clairement chaque thème.
6. **Production du rapport** : Rédiger le rapport final en expliquant les thèmes et en illustrant avec des extraits de données.

Ci-dessous le prompt général pour la réalisation de cette Phase 2 Méthode 2-2

"J'ai 448 réponses d'élèves sous forme de petites phrases que je voudrais analyser de manière qualitative en utilisant une analyse thématique. Voici les réponses sur le fichier Word.

Je veux que tu réalises les tâches suivantes :

1. Familiarise-toi avec les réponses et fournis un résumé global pour comprendre le contenu et le contexte.

2. Identifie et code les segments de texte pertinents avec des étiquettes courtes qui résument chaque segment.

3. Groupe les codes similaires en thèmes potentiels.

4. Révise et affine les thèmes pour t'assurer qu'ils représentent bien les données, et fournis des suggestions d'amélioration si nécessaire.

5. Définis et nomme clairement chaque thème et sous-thème

Un minimum de 20 thèmes et sous thèmes sont attendus.

Merci de m'aider à réaliser cette analyse complète tout en prenant en considération le contexte général sur la problématique et qui est présenté dans le Conceptual framework

Our concept of stigma is rooted in the framework proposed by Bos et al. [20], which was adapted from the one developed by Pryor et al. [21] (see Fig. 1). Stigma is categorized by these authors as belonging to one of four types. The public (or social) stigma is at the core of the model and refers to the social and psychological reactions of society to the person who has the stigmatized condition [22]. Self-stigma reflects the impact of stigma on the stigmatized person and is partly internalized through a reduction in self-worth and psychological distress. Stigma by association reflects the negative reactions to family and friends of stigmatized persons and their attitudes to this [23].

Then, once the stigma becomes institutionalized within society, the authors define the fourth category as “structural stigma”, when the ideological systems of society perpetuate the stigmatized status [21]. As stated above, those who internalize stigma often experience significant loss of self-esteem [22]. The relationship between self-concept and self-stigma is well documented in psychiatry. Stigmatized people have negative attitudes towards themselves as a reaction to their condition [24]. Self-concept should be distinguished from selfawareness and self-esteem. McConnell et al. [25] state that the awareness about the self is always influenced by the context. Over time, the person develops a self-concept, which interacts with self-esteem, self-knowledge, and selfawareness within the social context. In this article, we use the term self-concept as the cognitive description of one’s self (self-awareness) added to the opinion about one’sself (self-esteem).

References

20. Bos AER, et al. Stigma: advances in theory and research. Basic Appl Soc Psychol. 2013;35(1):1–9.

21. Pryor JB, et al. A dual-process model of reactions to perceived stigma. J Pers Soc Psychol. 2004;87(4):436–52.

22. Mittal D, et al. Empirical studies of self-stigma reduction strategies: a critical review of the literature. Psychiatr Serv. 2012;63(10):974–81.

23. Camp DL, Finlay WM, Lyons E. Is low self-esteem an inevitable consequence of stigma? An example from women with chronic mental health problems. Soc Sci Med. 2002;55(5):823–34.

24. Sirey JA, et al. Stigma as a barrier to recovery: perceived stigma and patientrated severity of illness as predictors of antidepressant drug adherence. Psychiatr Serv. 2001;52(12):1615–20.

English translation

Prompt Phase 2 using Method 2-2 (Structured request based on contextual learning and thematic qualitative analysis process)

Thematic analysis is a method for identifying, analyzing, and reporting patterns (themes) within data. The typical steps are as follows:

1. **Familiarization**: Reading and re-reading the data to become familiar with its content.
2. **Initial Coding**: Identifying and coding relevant segments of text linked to the research question.
3. **Searching for Themes**: Grouping the codes into potential themes.
4. **Reviewing Themes**: Verifying and refining the themes to ensure they accurately represent the data.
5. **Defining and Naming Themes**: Clearly defining and naming each theme.
6. **Producing the Report**: Writing the final report to explain the themes and illustrating them with data excerpts.

Below is the general prompt for conducting this Phase 2 using Method 2-2:

"I have 448 student responses in the form of short sentences that I would like to analyze qualitatively using thematic analysis. The responses are available in the Word document.

I would like you to perform the following tasks:

1. Familiarize yourself with the responses and provide a global summary to understand the content and context.
2. Identify and code relevant text segments with short labels that summarize each segment.
3. Group similar codes into potential themes.
4. Review and refine the themes to ensure they accurately represent the data, providing improvement suggestions if needed.
5. Clearly define and name each theme and sub-theme.

A minimum of 20 themes and sub-themes is expected.

Please help me complete this comprehensive analysis while taking into account the general context of the issue as presented in the conceptual framework."

Our concept of stigma is rooted in the framework proposed by Bos et al. [20], which was adapted from the one developed by Pryor et al. [21] (see Fig. 1). Stigma is categorized by these authors as belonging to one of four types. The public (or social) stigma is at the core of the model and refers to the social and psychological reactions of society to the person who has the stigmatized condition [22]. Self-stigma reflects the impact of stigma on the stigmatized person and is partly internalized through a reduction in self-worth and psychological distress. Stigma by association reflects the negative reactions to family and friends of stigmatized persons and their attitudes to this [23].

Then, once the stigma becomes institutionalized within society, the authors define the fourth category as “structural stigma”, when the ideological systems of society perpetuate the stigmatized status [21]. As stated above, those who internalize stigma often experience significant loss of self-esteem [22]. The relationship between self-concept and self-stigma is well documented in psychiatry. Stigmatized people have negative attitudes towards themselves as a reaction to their condition [24]. Self-concept should be distinguished from selfawareness and self-esteem. McConnell et al. [25] state that the awareness about the self is always influenced by the context. Over time, the person develops a self-concept, which interacts with self-esteem, self-knowledge, and selfawareness within the social context. In this article, we use the term self-concept as the cognitive description of one’s self (self-awareness) added to the opinion about one’sself (self-esteem).

References

20. Bos AER, et al. Stigma: advances in theory and research. Basic Appl Soc Psychol. 2013;35(1):1–9.

21. Pryor JB, et al. A dual-process model of reactions to perceived stigma. J Pers Soc Psychol. 2004;87(4):436–52.

22. Mittal D, et al. Empirical studies of self-stigma reduction strategies: a critical review of the literature. Psychiatr Serv. 2012;63(10):974–81.

23. Camp DL, Finlay WM, Lyons E. Is low self-esteem an inevitable consequence of stigma? An example from women with chronic mental health problems. Soc Sci Med. 2002;55(5):823–34.

24. Sirey JA, et al. Stigma as a barrier to recovery: perceived stigma and patientrated severity of illness as predictors of antidepressant drug adherence. Psychiatr Serv. 2001;52(12):1615–20.
