# Supplementary material for: Advancing AI-driven thematic analysis in qualitative research: a comparative study of nine generative models on Cutaneous Leishmaniasis data
Source: BMC Med Inform Decis Mak. 2025 Mar 10;25:124. doi: 10.1186/s12911-025-02961-5 (PMC11895178; doi:10.1186/s12911-025-02961-5)
Supplement: Supplementary file 11 — Supplementary Material 11: Additional file 6. Phase 2 Reference A Prompt Perplexity sub-themes generation [file 12911_2025_2961_MOESM11_ESM.pdf]

Original French request >>>> Prompt Perplexity Pro

Je souhaite que vous analysiez le texte suivant et que vous présentiez les thèmes principaux et les sous-thèmes sous forme de tableau. Le tableau doit inclure les colonnes suivantes : Thème principal, Sous-thème Référence A, et Brève explication de sous-thème de la référence A.

Le texte à analyser est la copie intégrale de la section résultats de l'article publié précédemment et qui est disponible sous format de fichier PDF téléchargé (Additional file 6bis).

Selon la figure, faites sortir les cinq thémes suivants (Self-concept, Body image, Social stigma, Self-stigma, Health seeking Behaviour) . Alors qu'on propose que les sous thèmes soient au nombre exact de 24.

Translation of the prompt

Prompt Perplexity Pro

I would like you to analyse the following text and present the main themes and sub-themes in a table format. The table should include the following columns: Main Theme, Sub-theme (Reference A), and a Brief Explanation of the Sub-theme from Reference A.

The text to be analysed is the complete results section of the previously published article, available as a downloadable PDF file (Additional file 6bis).

Based on the figure, please extract the following five themes: Self-Concept, Body Image, Social Stigma, Self-Stigma, and Health-Seeking Behaviour. The sub-themes are to number exactly 24.

Results generated by using this prompt as shown in the video demonstration

Tableau des 24 Sous thèmes issues de l'analyse thématique initiale mentionnée comme Référence A et ayant servi de comparaison de la précision du processus de synthèse qualitative.

Thème Principal	Sous-thème Référence A	Brève explication de sous thème de la référence A
Self-Concept	Self-Confidence	Perte de confiance en soi en raison des cicatrices
	Self-Esteem	Diminution de l'estime de soi liée à l'apparence
	Self-Awareness	Conscience accrue de l'apparence physique
	Self-Contempt	Dégoût de soi à cause des cicatrices
Body Image	Body Beauty	Préoccupation concernant la beauté corporelle
	Face Appearance	Importance de l'apparence du visage
	Scars Cosmetic Effects	Effets cosmétiques des cicatrices
Social Stigma	Family Relationship	Relations familiales affectées par la peur de contagion
	Avoidance by Others	Évitement par les autres à cause des cicatrices
	Social Contempt	Mépris social en raison des cicatrices
	Marriage Difficulties	Difficultés de mariage liées à l'apparence physique
	Fear of rejection	Peur du rejet social et de la contagion
Self-Stigma	Embarrassment	Sentiment de gêne lié aux cicatrices
	Shame	Honte de son apparence en public
	Anxiety	Anxiété due à la perception des cicatrices
	Sadness	Tristesse liée à la condition physique
	Depression	Dépression causée par les cicatrices
	Suicidal Ideas	Idées suicidaires associées aux cicatrices
Health Seeking Behaviour	Traditional Remedies	Recours aux remèdes traditionnels pour traiter les cicatrices
	Conventional Treatments	Traitements médicaux conventionnels souvent inefficaces
	Spiritual Healing	Acceptation spirituelle de la maladie (volonté de Dieu)
	Coping Strategies	Stratégies de coping pour dissimuler les cicatrices
	Psychological Support	Besoin de soutien psychologique pour faire face aux cicatrices
	Government Intervention	Appel à l'intervention gouvernementale pour des soins abordables

Translation of the results

Results generated by this prompt

The table below includes 24 sub-themes derived from the initial thematic analysis, referred to as Reference A, which served as a basis for comparison in the precision of the qualitative synthesis process.

Main Theme	Sub-theme (Reference A)	Brief Explanation of Sub-theme from Reference A
Self-Concept	Self-Confidence	Loss of confidence due to scarring.
	Self-Esteem	Decrease in self-esteem linked to physical appearance.
	Self-Awareness	Heightened awareness of physical appearance.
	Self-Contempt	Self-disgust caused by scars.
Body Image	Body Beauty	Concern about bodily beauty.
	Face Appearance	Importance of facial appearance.
	Scars Cosmetic Effects	Cosmetic effects of scarring.
Social Stigma	Family Relationship	Family relationships affected by fear of contagion.
	Avoidance by Others	Avoidance by others due to visible scars.
	Social Contempt	Social contempt resulting from scars.
	Marriage Difficulties	Marriage difficulties caused by physical appearance.
	Fear of Rejection	Fear of social rejection and contagion.
Self-Stigma	Embarrassment	Feelings of embarrassment due to scars.
	Shame	Shame regarding appearance in public.
	Anxiety	Anxiety stemming from the perception of scars.
	Sadness	Sadness related to physical condition.
	Depression	Depression caused by scarring.
	Suicidal Ideas	Suicidal thoughts associated with scars.
Health-Seeking Behaviour	Traditional Remedies	Use of traditional remedies for treating scars.
	Conventional Treatments	Conventional medical treatments often deemed ineffective.
	Spiritual Healing	Spiritual acceptance of the condition as the will of God.
	Coping Strategies	Coping strategies to conceal scars.

	Psychological Support	Need for psychological support to cope with scarring.
	Government Intervention	Call for government intervention to provide affordable care.
