# Supplementary material for: Advancing AI-driven thematic analysis in qualitative research: a comparative study of nine generative models on Cutaneous Leishmaniasis data
Source: BMC Med Inform Decis Mak. 2025 Mar 10;25:124. doi: 10.1186/s12911-025-02961-5 (PMC11895178; doi:10.1186/s12911-025-02961-5)
Supplement: Supplementary file 12 — Supplementary Material 12: Additional file 6bis. Phase 2 Reference A Text extracted from the primary article. Additional file 6ter. Phase 2 Reference A Perplexity results video demonstration. YouTube [35] [file 12911_2025_2961_MOESM12_ESM.pdf]

Phase 2 Méthode Référence A

Texte à analyser

Ref : Bennis, I., Thys, S., Filali, H. *et al.* Psychosocial impact of scars due to cutaneous leishmaniasis on high school students in Errachidia province, Morocco. *Infect Dis Poverty* **6**, 46 (2017). https://doi.org/10.1186/s40249-017-0267-5

The text analysis of the open-ended question about the possible CL impact is presented below in three thematic sections: 1) CL severity and body image; 2) CL scars and stigma; and 3) dealing with scars. Twenty percent of the boys (52/258) and thirteen percent of the girls (24/190) did not answer the open-ended question. CL severity and body image The students' perception of the severity of CL can be summarized in three interrelated words: "dangerous", "serious", and "deathly". Dangerous CL is perceived by many as dangerous because of the fear of contagion and the risk of transmitting CL to relatives: "It will lead to social isolation and the person will live away from others due to fear of contaminating them." (MT305). This fear includes the fear of being rejected by their own relatives: "The person affected is afraid from this disease as there is a risk it could worsen and not heal. He will be disturbed by its appearance if seen [by others] and be afraid that his friends and family will become distant from him and reject him for fear of being affected by the same disease." (MR081). Another boy wrote: "He will not be able to share meals with his family." (MT360). Serious One girl wrote: "The affected person will be disgusted to see himself in the mirror." (FT186). Another wrote: "Sometimes the affected people end up hating themselves." (FR044). Differences according to gender were observed in terms of use of the word "scar".Manygirlsbutveryfew boys thought that CL scars are considerably worse for a woman than for a man. According to our respondents, girls are more concerned about the effects of CL scars, the appearance of their face, and the beauty of their body. Three boys reported that men are more heavily affected by CL, as they lose their masculinity as a result of the disease. One boy evoked the need for psychological support: "... Also it causes the patient psychological and dermatological effects over a long period of time, which requires a visit to a psychiatrist." (MR121). One girl noted that both sexes suffered equally due to the disease (FR031). Deathly A few respondents thought (erroneously) that CL can have a fatal outcome and wrote that death is the natural outcome of the disease. Interestingly, others evoked a fatal outcome in the context of suicidal thoughts, as stated by one boy: "This disease often influences some people because it leaves scars from the beginning that lead a person to commit suicide." (MR123). In addition, four girls cited the terms "depression" and "suicidal ideations" in their answers. One girl noted: "Cutaneous leishmaniasis influences the psychological state and leads to death." (FT289). CL scars and stigma The fear of facing others was frequently mentioned by both sexes. "...The affected person cannot talk about his disease due to fear of being rejected by people," noted one boy (MR132). "The person affected cannot show this to his friends because they will not want to sit with him," said another boy (MR160). The attitude of friends and family influences the psychological state of the affected person, as reported by one girl: "She will have depression, a durable fear, and shame. She does not have the absolute courage to sit with her friends for fear of their mockery." (FT237). Additionally, girls noted components of self-stigma such as shame, embarrassment, depression, and self-contempt. One girl noted: "The affected person is very worried....and feels ashamed in the presence of people. She prohibits herself to go to rallies because of this disease." (FT 225). Another wrote: "The sequelae of leishmaniasis negatively influence the condition of the affected person. A psychological complex will develop and she will be ashamed to appear in front of friends because it is a mark of shame and contempt." (FT265). The answers show how self-stigma and social stigma are linked. When the scar is formed, people tend to stare at it and the gaze of others induces social rejection of the affected person. "It affects me every time I meet my friends. They look at my scar, it diminishes my value in front of people," reported a girl who experienced CL (FR009). Consequently, communication barriers emerge: "The scar is a mark of shame and contempt. The affected person will be unable to cope with society due to fear of social

discrimination and contempt.” (MT413). One girl noted: “She will become closed off (introverted) and she will not talk to people and she is not going to love looking at herself because of these scars.” (FT243). The affected person no longer has the same appearance as before, and she/he is now different from others. The negative behavior of others leads to a feeling of isolation and difficulty in everyday life: “There is a feeling of isolation and loneliness, and a lack of stability in daily life because of the negative attitude of society towards the patient,” wrote one boy (MR120). Another girl wrote: “For a girl when the disease leaves a mark on the face for example, the girl will think that is dangerous for her beauty, which will influence her psychological state. Especially in our traditional society that is absolutely not lenient towards those who have spots on the face because they think it is hereditary.” (FT194). CL can lead to ‘social death’ as explained by one girl: “Moroccan society is not merciful and judges people’s appearance.” (FT241). Indeed, some participants linked CL scars with diminished chances of getting married: “Often the scars in affected men and especially in affected women are a barrier to getting married because the scars are visible, something that is not tolerated by fiancés.” (FR049). Another girl noted: “Women have more fear on their faces, afraid that the young man who comes forward to ask for her hand will disappear after having seen these stains.” (FR001). A girl who previously experienced CL noted that the possibility of not getting married is a problem only for women: “She will be afraid about her future especially for the wedding. Meanwhile, in our society, an affected boy remains a man. There is no harm if he has scars.” (FT202). In addition, the fear to meet others leads to a lower selfconcept: “The girl is ashamed to show her face; scars can prevent her to leave the house, thereby increasing her psychological suffering” (FR018). “Why me?” is a question that a person affected by CL often asks: “Why am I the person who has this disease and carries this mark on the face?” reported a boy previously affected by CL (MR153). Dealing with scars Half of the respondents wrote that treatment for CL scars was available in hospitals from general practitioners or dermatologists. However, the affected person slowly understands after trying various treatments that these scars will never disappear: “The psychological state of the affected person can worsen after receiving treatment because the problem is that scars never disappear [even after treatment],” noted a boy previously affected by CL (MR116). “The fear and worry regarding the lack of treatment for this disease is the real problem for a person affected by it,” added a girl also previously affected by CL (FR011). The ineffectiveness of delayed treatment was also something stressed upon: “We need prompt treatment for this disease to avoid any effect on the psychological state of the person affected.” (MR174). In addition, one girl mentioned the problem of unaffordable treatment (FT224). Nevertheless, a number of responses included questions about what was indeed an effective treatment against CL scars. “Is there a way to heal the scars?” wrote one girl (FR 068). Another girl wrote: “Is there a treatment for scars? I wish there was a yes answer, there was a cure!” (FT195). Various coping strategies were described as a solution to deal with the scars, especially among girls, focusing on ways to hide the scars temporarily: “I am personally affected by this disease. I suffer from its consequences. The scar on my face has created a big problem in my life. I am obliged to put cream to try to hide it before going anywhere.” (FR035). Alternatively, spiritual factors such as ‘God’s will’ and ‘destiny’ were the third care-seeking explanations. Two boys and one girl mentioned that God decided who is to be affected by the disease and who will be healed. One boy also suggested these scars need to be accepted “... After a while, he will get used to those scars and it will become normal.” (MR096). Contrastingly, other students asked for direct government intervention: “This disease could leave psychological trouble in the affected person. That is why the government and the concerned commission must find a solution for this disease, it’s not the duty of the population,” wrote one boy (MR093). Another boy noted that the population along with the policymakers should be involved in the prevention of this dangerous disease (MT355).

Students' answers to the open-ended questions required the verbatim transcription in the Arabic language and then translation into French. The text analysis was performed with NVivo software version 10 (QSR International Melbourne Australia). The coding followed a deductive approach based on the conceptual framework (see Fig. 1). An inductive analysis was done to extend the generated codes (see Fig. 2) in order to define the factors which influenced the impact of CL scars on the patient's psychological state. Five major themes were elucidated: perception of body image, self-stigma, social stigma, selfconcept, and health-seeking behavior. Attributes of gender, area of residence, and personal CL experience were also used to classify answers in order to examine relationships within these qualitative data.

Self-concept	
Self-confidence	Self-esteem
Self-awareness	Self-contempt

Body image	
Body beauty	Face appearance
Scars cosmetic effects	

Social stigma	
Family relationship	Avoidance by others
Social contempt	Marriage difficulties
Fear	

Self-stigma	
Embarrassment	Shame
Anxiety	Sadness
Depression	Suicidal ideas

Health seeking behaviour	
Traditional remedies	Conventional treatments
Spiritual healing	Coping strategies
