# Supplementary material for: Advancing AI-driven thematic analysis in qualitative research: a comparative study of nine generative models on Cutaneous Leishmaniasis data
Source: BMC Med Inform Decis Mak. 2025 Mar 10;25:124. doi: 10.1186/s12911-025-02961-5 (PMC11895178; doi:10.1186/s12911-025-02961-5)
Supplement: Supplementary file 15 — Supplementary Material 15: Additional file 8. Prompt Phase 3A. Additional file 8bis. Phase 3A All A.I. Models results 2025-01-06-video demonstration. YouTube [36] [file 12911_2025_2961_MOESM15_ESM.pdf]

Phase 3 A prompts used

Reference sub-themes Phase 3 using the Phase 2 PDF files (Method 2-1 and Method 2-2 and Method Reference A)

Please replace the letter X with the appropriate letter mentioned in the downloaded files, then follow the model type of the matrix in the image capture to provide a detailed matrix in relation to the five Main themes, all the 24 sub-themes and the Brief explanation of the sub-theme of reference A, by presenting the results of X1, X2, X3, X4 and the association of X1+X2 (If present in a box, it is considered present in both X1 & X2) and also the association X3+X4. While specifying whether they are present or absent for each box in the columns of X1, X2, X3, X4 and the association X1 and X2 and the association X3 and X4 and the association X1+X2+X3+X4. Please pay close attention to the qualitative analysis of the sub-themes and the similarities between the sub-themes and do not miss any sub-themes that have the same meaning and connotation in relation to the 24 sub-themes of reference A. Carefully examine each document to accurately identify the presence or absence of each theme Note the variations in frequency and importance between documents Check for exact matches between themes rather than assuming their presence. Also, please add, at the bottom of the table, the sub-themes that are only mentioned in X1 or X2 or X3 or X4 and that are not directly mentioned in reference A. While trying to link these sub-themes to the sub-themes of reference A and to propose the most accurate matrix possible. Double check all data A for absent and P for present and present this in the form of an Excel type matrix.

Then a last verification

Based on the results of the additional sub-themes that may be linked to the reference A, recorrect the table with justification of any “A” to ”P” case change. Then, give only the (new insights from any new Main theme or new Subtheme that does not match directly or indirectly the explanation and information present into Reference A.
