# Supplementary material for: Advancing AI-driven thematic analysis in qualitative research: a comparative study of nine generative models on Cutaneous Leishmaniasis data
Source: BMC Med Inform Decis Mak. 2025 Mar 10;25:124. doi: 10.1186/s12911-025-02961-5 (PMC11895178; doi:10.1186/s12911-025-02961-5)
Supplement: Supplementary file 17 — Supplementary Material 17: Additional file 9. Phase 3B Code Python used to calculate Jaccard Index [file 12911_2025_2961_MOESM17_ESM.pdf]

Python coding used to calculate Jaccard Index

```python
import pandas as pd
file_path =
df = pd.read_excel(file_path, sheet_name='Sheet1')
print(df.head())
pd.set_option('display.max_rows', None)
pd.set_option('display.max_columns', None)
def calculate_jaccard_with_denominator_details(df, col1, col2):
    intersection = df[(df[col1] == 1) & (df[col2] == 1)].shape[0]
    union = df[(df[col1] == 1) | (df[col2] == 1)].shape[0]
    jaccard_index = intersection / union
    size_A = df[df[col1] == 1].shape[0]
    size_X = df[df[col2] == 1].shape[0]
    denominator = size_A + size_X - intersection
    print(f"Nombre d'éléments en commun (intersection) : {intersection}")
    print(f"Nombre total d'éléments uniques (union) : {union}")
    print(f"|A| (Nombre d'éléments dans A) : {size_A}")
    print(f"|X| (Nombre d'éléments dans X) : {size_X}")
    print(f"Dénominateur (|A| + |X| - |AnX|) : {denominator}")
    print(f"Calcul détaillé : {intersection} / {denominator} = {jaccard_index}\n")
    return jaccard_index
column_pairs = [
    ('A', 'B1'), ('A', 'B2'), ('A', 'B3'), ('A', 'B4'), ('A', 'B1_ B2'), ('A', 'B3_B4'), ('A', 'B1_B2_B3_B4'),
    ('A', 'C1'), ('A', 'C2'), ('A', 'C3'), ('A', 'C4'), ('A', 'C1_C2'), ('A', 'C3_C4'), ('A', 'C1_C2_C3_C4'),
    ('A', 'D1'), ('A', 'D2'), ('A', 'D3'), ('A', 'D4'), ('A', 'D1_D2'), ('A', 'D3_D4'), ('A', 'D1_D2_D3_D4'),
    ('A', 'E1'), ('A', 'E2'), ('A', 'E3'), ('A', 'E4'), ('A', 'E1_E2'), ('A', 'E3_E4'), ('A', 'E1_E2_E3_E4'),
    ('A', 'F1'), ('A', 'F2'), ('A', 'F3'), ('A', 'F4'), ('A', 'F1_F2'), ('A', 'F3_F4'), ('A', 'F1_F2_F3_F4'),
    ('A', 'G1'), ('A', 'G2'), ('A', 'G3'), ('A', 'G4'), ('A', 'G1_G2'), ('A', 'G3_G4'), ('A', 'G1_G2_G3_G4'),
    ('A', 'H1'), ('A', 'H2'), ('A', 'H3'), ('A', 'H4'), ('A', 'H1_H2'), ('A', 'H3_H4'), ('A', 'H1_H2_H3_H4'),
    ('A', 'K1'), ('A', 'K2'), ('A', 'K3'), ('A', 'K4'), ('A', 'K1_K2'), ('A', 'K3_K4'), ('A', 'K1_K2_K3_K4'),
    ('A', 'M1'), ('A', 'M2'), ('A', 'M3'), ('A', 'M4'), ('A', 'M1_M2'), ('A', 'M3_M4'), ('A', 'M1_M2_M3_M4'),
]
for col1, col2 in column_pairs:
    jaccard_index = calculate_jaccard_with_denominator_details(df, col1, col2)
    print(f'Jaccard ({col1}, {col2}) = {jaccard_index}\n')
```

```python
import pandas as pd

file_path = XXXXXX

df = pd.read_excel(file_path, sheet_name='Sheet1')

print(df.head())

pd.set_option('display.max_rows', None)

pd.set_option('display.max_columns', None)

def calculate_jaccard_with_denominator_details(df, col1, col2):

    intersection = df[(df[col1] == 1) & (df[col2] == 1)].shape[0]

    union = df[(df[col1] == 1) | (df[col2] == 1)].shape[0]

    jaccard_index = intersection / union

    size_A = df[df[col1] == 1].shape[0]

    size_X = df[df[col2] == 1].shape[0]

    denominator = size_A + size_X - intersection

    print(f"Nombre d'éléments en commun (intersection) : {intersection}")

    print(f"Nombre total d'éléments uniques (union) : {union}")
```

```
    print(f"|A| (Nombre d'éléments dans A) : {size_A}")
    print(f"|X| (Nombre d'éléments dans X) : {size_X}")
    print(f"Dénominateur (|A| + |X| - |A∩X|) : {denominator}")
    print(f"Calcul détaillé : {intersection} / {denominator} = {jaccard_index}\n")
    return jaccard_index
column_pairs = [
    ('A', 'B1'), ('A', 'B2'), ('A', 'B3'), ('A', 'B4'), ('A', 'B1_ B2'), ('A', 'B3_B4'), ('A', 'B1_B2_B3_B4'),
    ('A', 'C1'), ('A', 'C2'), ('A', 'C3'), ('A', 'C4'), ('A', 'C1_C2'), ('A', 'C3_C4'), ('A', 'C1_C2_C3_C4'),
    ('A', 'D1'), ('A', 'D2'), ('A', 'D3'), ('A', 'D4'), ('A', 'D1_D2'), ('A', 'D3_D4'), ('A', 'D1_D2_D3_D4'),
    ('A', 'E1'), ('A', 'E2'), ('A', 'E3'), ('A', 'E4'), ('A', 'E1_E2'), ('A', 'E3_E4'), ('A', 'E1_E2_E3_E4'),
    ('A', 'F1'), ('A', 'F2'), ('A', 'F3'), ('A', 'F4'), ('A', 'F1_F2'), ('A', 'F3_F4'), ('A', 'F1_F2_F3_F4'),
   ('A', 'G1'), ('A', 'G2'), ('A', 'G3'), ('A', 'G4'), ('A', 'G1_G2'), ('A', 'G3_G4'), ('A', 'G1_G2_G3_G4'),
   ('A', 'H1'), ('A', 'H2'), ('A', 'H3'), ('A', 'H4'), ('A', 'H1_H2'), ('A', 'H3_H4'), ('A', 'H1_H2_H3_H4'),
 ('A', 'K1'), ('A', 'K2'), ('A', 'K3'), ('A', 'K4'), ('A', 'K1_K2'), ('A', 'K3_K4'), ('A', 'K1_K2_K3_K4'),
('A', 'M1'), ('A', 'M2'), ('A', 'M3'), ('A', 'M4'), ('A', 'M1_M2'), ('A', 'M3_M4'), ('A', 'M1_M2_M3_M4'),
]
for col1, col2 in column_pairs:
    jaccard_index = calculate_jaccard_with_denominator_details(df, col1, col2)
    print(f'Jaccard ({col1}, {col2}) = {jaccard_index}\n')
```

The python results

B : LlaMA 3.1

Jaccard (A, B1) = 14 / (24 + 14 - 14) = 14 / 24 = 0.583
Jaccard (A, B2) = 13 / (24 + 13 - 13) = 13 / 24 = 0.542
Jaccard (A, B3) = 14 / (24 + 14 - 14) = 14 / 24 = 0.583
Jaccard (A, B4) = 7 / (24 + 7 - 7) = 7 / 24 = 0.292
Jaccard (A, B1_B2) = 16 / (24 + 16 - 16) = 16 / 24 = 0.667
Jaccard (A, B3_B4) = 15 / (24 + 15 - 15) = 15 / 24 = 0.625
Jaccard (A, B1_B2_B3_B4) = 19 / (24 + 19 - 19) = 19 / 24 = 0.792

C : NotebookLM

Jaccard (A, C1) = 13 / (24 + 13 - 13) = 13 / 24 = 0.542
Jaccard (A, C2) = 12 / (24 + 12 - 12) = 12 / 24 = 0.5
Jaccard (A, C3) = 14 / (24 + 14 - 14) = 14 / 24 = 0.583
Jaccard (A, C4) = 13 / (24 + 13 - 13) = 13 / 24 = 0.542
Jaccard (A, C1_C2) = 13 / (24 + 13 - 13) = 13 / 24 = 0.542
Jaccard (A, C3_C4) = 13 / (24 + 13 - 13) = 13 / 24 = 0.542
Jaccard (A, C1_C2_C3_C4) = 15 / (24 + 15 - 15) = 15 / 24 = 0.625

D : Gemini1.5 AdUltra

Jaccard (A, D1) = 13 / (24 + 13 - 13) = 13 / 24 = 0.542
Jaccard (A, D2) = 14 / (24 + 14 - 14) = 14 / 24 = 0.583
Jaccard (A, D3) = 14 / (24 + 14 - 14) = 14 / 24 = 0.583
Jaccard (A, D4) = 19 / (24 + 19 - 19) = 19 / 24 = 0.792
Jaccard (A, D1_D2) = 14 / (24 + 14 - 14) = 14 / 24 = 0.583
Jaccard (A, D3_D4) = 17 / (24 + 17 - 17) = 17 / 24 = 0.708
Jaccard (A, D1_D2_D3_D4) = 18 / (24 + 18 - 18) = 18 / 24 = 0.75

E : Claude 3.5 Sonnet

Jaccard (A, E1) = 15 / (24 + 15 - 15) = 15 / 24 = 0.625
Jaccard (A, E2) = 13 / (24 + 13 - 13) = 13 / 24 = 0.542
Jaccard (A, E3) = 17 / (24 + 17 - 17) = 17 / 24 = 0.708
Jaccard (A, E4) = 16 / (24 + 16 - 16) = 16 / 24 = 0.667
Jaccard (A, E1_E2) = 12 / (24 + 12 - 12) = 12 / 24 = 0.5
Jaccard (A, E3_E4) = 20 / (24 + 20 - 20) = 20 / 24 = 0.833
Jaccard (A, E1_E2_E3_E4) = 20 / (24 + 20 - 20) = 20 / 24 = 0.833

F : Chat GPTo1 PRO

Jaccard (A, F1) = 23 / (24 + 23 - 23) = 23 / 24 = 0.958
Jaccard (A, F2) = 23 / (24 + 23 - 23) = 23 / 24 = 0.958
Jaccard (A, F3) = 23 / (24 + 23 - 23) = 23 / 24 = 0.958
Jaccard (A, F4) = 24 / (24 + 24 - 24) = 24 / 24 = 1.0
Jaccard (A, F1_F2) = 23 / (24 + 23 - 23) = 23 / 24 = 0.958
Jaccard (A, F3_F4) = 24 / (24 + 24 - 24) = 24 / 24 = 1.0
Jaccard (A, F1_F2_F3_F4) = 24 / (24 + 24 - 24) = 24 / 24 = 1.0

G : Chat GPTo1

Jaccard (A, G1) = 21 / (24 + 21 - 21) = 21 / 24 = 0.875
Jaccard (A, G2) = 18 / (24 + 18 - 18) = 18 / 24 = 0.75
Jaccard (A, G3) = 23 / (24 + 23 - 23) = 23 / 24 = 0.96
Jaccard (A, G4) = 23 / (24 + 23 - 23) = 23 / 24 = 0.96
Jaccard (A, G1_G2) = 21 / (24 + 21 - 21) = 21 / 24 = 0.875
Jaccard (A, G3_G4) = 23 / (24 + 23 - 23) = 23 / 24 = 0.96
Jaccard (A, G1_G2_G3_G4) = 24 / (24 + 24 - 24) = 24 / 24 = 1.0

H : GrokV2

Jaccard (A, H1) = 22 / (24 + 22 - 22) = 22 / 24 = 0.92
Jaccard (A, H2) = 21 / (24 + 21 - 21) = 21 / 24 = 0.875
Jaccard (A, H3) = 21 / (24 + 21 - 21) = 21 / 24 = 0.875
Jaccard (A, H4) = 21 / (24 + 21 - 21) = 21 / 24 = 0.875
Jaccard (A, H1_H2) = 23 / (24 + 23 - 23) = 23 / 24 = 0.96
Jaccard (A, H3_H4) = 23 / (24 + 23 - 23) = 23 / 24 = 0.96
Jaccard (A, H1_H2_H3_H4) = 24 / (24 + 24 - 24) = 24 / 24 = 1.0

K : DeepSeek V3

Jaccard (A, K1) = 18 / (24 + 18 - 18) = 18 / 24 = 0.75
Jaccard (A, K2) = 15 / (24 + 15 - 15) = 15 / 24 = 0.625
Jaccard (A, K3) = 17 / (24 + 17 - 17) = 17 / 24 = 0.708
Jaccard (A, K4) = 24 / (24 + 24 - 24) = 24 / 24 = 1.0
Jaccard (A, K1_K2) = 20 / (24 + 20 - 20) = 20 / 24 = 0.833
Jaccard (A, K3_K4) = 24 / (24 + 24 - 24) = 24 / 24 = 1.0
Jaccard (A, K1_K2_K3_K4) = 24 / (24 + 24 - 24) = 24 / 24 = 1.0

M : Gemini2.0 Advanced

Jaccard (A, M1) = 19 / (24 + 19 - 19) = 19 / 24 = 0.792
Jaccard (A, M2) = 17 / (24 + 17 - 17) = 17 / 24 = 0.708
Jaccard (A, M3) = 21 / (24 + 21 - 21) = 21 / 24 = 0.875
Jaccard (A, M4) = 22 / (24 + 22 - 22) = 22 / 24 = 0.916
Jaccard (A, M1_M2) = 21 / (24 + 21 - 21) = 21 / 24 = 0.875
Jaccard (A, M3_M4) = 22 / (24 + 22 - 22) = 22 / 24 = 0.916
Jaccard (A, M1_M2_M3_M4) = 22 / (24 + 22 - 22) = 22 / 24 = 0.916
