# Supplementary material for: Advancing AI-driven thematic analysis in qualitative research: a comparative study of nine generative models on Cutaneous Leishmaniasis data
Source: BMC Med Inform Decis Mak. 2025 Mar 10;25:124. doi: 10.1186/s12911-025-02961-5 (PMC11895178; doi:10.1186/s12911-025-02961-5)
Supplement: Supplementary file 19 — Supplementary Material 19: Additional file 10bis. FGHK Additional themes & sub-themes insights issued from Phase 3A. Additional file 10ter. Grounded theory analysis 2025-01-06 video demonstration. YouTube [40] [file 12911_2025_2961_MOESM19_ESM.pdf]

The final prompt :

Trianguler ce que vous avez trouvez par rapport aux insights suivants tout en présentant un cadre conceptuel original et non classique :

New Insights: Main Themes and Subthemes
Beyond the corrections to the existing table, the thematic analysis reveals several new main themes and sub-themes not directly or indirectly addressed in Reference A. These insights shed light on additional psychological, social, and structural dimensions of the impact of CL.

1. Stigma by Association
This theme, describes the fear and negative perceptions experienced by individuals associated with someone suffering from CL. It extends beyond the individual with CL to their family and close circle.

Fear of transmitting the disease to loved ones: This anxiety contributes to self-imposed isolation and strained relationships, as individuals fear becoming a source of infection.

Negative reactions from family and friends: The fear of contagion can lead to avoidance and rejection from those closest to the individual, exacerbating social isolation and impacting support systems.

2. Structural Stigma
This theme highlights the impact of societal structures and systems on the experience of individuals with CL. It emphasizes the lack of adequate support, resources, and understanding at a broader level.

Criticism towards the healthcare system: Several responses express dissatisfaction with the healthcare system's handling of CL, pointing to limited access to specialized care, insufficient resources, and the high cost of treatment.

Lack of public awareness and education: There is a clear need for comprehensive public health campaigns to educate communities about CL, its transmission, and management. The absence of such initiatives perpetuates myths, fears, and discriminatory attitudes.

Government negligence and inadequate policies: Some responses highlight the perceived lack of attention from authorities in addressing the public health concerns associated with CL. They call for greater government intervention in terms of prevention, treatment access, and public awareness programs.

3. Gendered Experiences of Stigma
Sources678 consistently emphasize the heightened vulnerability of women and girls affected by CL. They face greater social pressure regarding physical appearance and marriageability, making the psychological impact of CL scars more severe.

Intensified fears related to marriage prospects: Women express profound anxieties about rejection by potential partners and the social stigma attached to visible scars, impacting their self-esteem and future prospects.

Heightened societal pressure on beauty and femininity: The presence of scars challenges traditional notions of beauty and femininity, leading to feelings of inadequacy and unworthiness. This reinforces the need for culturally sensitive interventions that address the specific concerns of women and girls.

4. Coping Mechanisms and Resilience
Despite the pervasive negative experiences, several individuals demonstrate resilience and employ diverse coping mechanisms to navigate the challenges posed by CL.

Minimization and Normalization: Some individuals adapt to their condition by downplaying its severity or considering it a common occurrence. This helps them maintain a sense of normalcy and manage the emotional distress.

Religious Faith and Spirituality: Reliance on faith, prayer, and acceptance of divine will emerges as a significant source of strength and coping for some individuals.

Concealment and Camouflage: Individuals actively seek to hide their scars using makeup, creams, clothing, or even considering surgical options. These strategies, while potentially helpful in managing social interactions, also reflect the intense pressure to conform to societal beauty standards.

These new insights, absent from Reference A, demonstrate the complex interplay of individual experiences, social perceptions, and systemic factors that contribute to the multifaceted impact of CL. Understanding these nuanced dimensions can inform the development of comprehensive interventions that address the medical, psychological, and social challenges associated with CL.

Beyond aligning sub-themes, the G documents introduce novel insights not directly or indirectly covered by Reference A.

These fall under broader themes:
1. Impact of CL on Mental Well-being: G documents, particularly G4, delve deeper into the potential for developing mental health disorders beyond depression and anxiety. The sub-themes:

"Development of Psychiatric Disorders"

"Risk of Depression and Anxiety Disorders"
highlight a more severe and nuanced understanding of CL's mental health implications. This suggests the need for psychological support extending beyond coping mechanisms to address potential clinical diagnoses and provide specialised mental healthcare.

2. Barriers to Seeking Help: G4 explicitly identifies factors hindering individuals from seeking medical help, which are not directly present in Reference A. These include:

"Shame Preventing from Seeking Help"5

"Fear of Stigmatisation Delaying Treatment"5

"Lack of Knowledge on Treatment Options"5
These barriers highlight the complex interplay between social stigma, individual emotions, and healthcare access.

Addressing these obstacles requires multifaceted interventions, including:

Public awareness campaigns to reduce stigma and encourage help-seeking.

Improving healthcare accessibility and affordability to alleviate financial barriers.

Providing clear and accessible information about CL, its treatment options, and support resources.

3. Physical Pain and Discomfort: While Reference A focuses on the visual impact of scars, G4 acknowledges the physical pain and discomfort associated with CL lesions. This aspect is reflected in the sub-themes:

"Physical Symptoms of the Disease"

"Pain and Discomfort of the Lesions"

"Impact on Physical Health Beyond Appearance"

This insight broadens the understanding of CL's impact beyond the psychosocial realm, emphasising the need to address the physical suffering and potential long-term health consequences. This necessitates a holistic approach to treatment and care, integrating pain management, wound care, and physical rehabilitation alongside psychological support and social interventions.

In conclusion, while the G documents largely align with the core themes of Reference A, they provide valuable new insights into the impact of CL, particularly regarding mental health, barriers to help-seeking, and the experience of physical pain. These findings enrich the understanding of CL's multifaceted consequences and underscore the need for comprehensive interventions encompassing medical, psychological, social, and educational aspects. This holistic approach is crucial for effectively addressing the burden of CL and improving the lives of those affected.

Beyond the revised matrix, the H documents reveal several crucial insights that expand upon the framework provided by Reference A, highlighting the multifaceted nature of CL's impact:

1. Impact on Physical Health:

Physical Symptoms and Discomfort: H4 explicitly mentions "physical symptoms" and "pain and discomfort of the lesions," highlighting that CL's impact extends beyond cosmetic concerns and involves tangible physical suffering. This adds a new dimension to understanding the burden of CL, emphasizing the need for holistic treatment addressing both physical and psychological well-being.

●

Impact on Physical Health Beyond Appearance: H4 underscores that the disease's impact extends beyond visible scars and can affect overall physical health. This broader perspective suggests potential long-term consequences requiring further investigation and comprehensive care that goes beyond addressing immediate symptoms.

2. Mental Health Risks:

Development of Psychiatric Disorders: H4 raises the alarming possibility of developing psychiatric disorders as a consequence of CL, indicating a potentially severe and long-lasting mental health impact. This underscores the urgent need for mental health support services and interventions tailored to individuals struggling with the psychological burden of CL.

Risk of Depression and Anxiety Disorders: H4 explicitly mentions the risk of these specific disorders, highlighting the need for proactive mental health screening and support for individuals diagnosed with CL. Early intervention and access to mental healthcare are crucial to mitigate the potential for long-term mental health challenges.

3. Barriers to Care:

Shame Preventing from Seeking Help: H4 identifies shame as a significant barrier to seeking treatment, emphasizing the social stigma associated with CL and its detrimental impact on help-seeking behaviour. This underscores the need for community-based interventions and awareness campaigns to destigmatize CL and encourage early diagnosis and treatment.

Fear of Stigmatization Delaying Treatment: Similarly, H4 highlights the fear of social stigma as a factor delaying treatment, further emphasizing the urgency of addressing societal perceptions and creating a supportive environment for individuals affected by CL. Public education and advocacy are crucial to combat stigma and promote timely access to care.

Lack of Knowledge on Treatment Options: H4 reveals a gap in knowledge regarding available treatment options, highlighting a critical need for educational interventions and accessible information dissemination about both conventional and traditional treatments. Empowering individuals with knowledge about their options can facilitate informed decision-making and improve treatment adherence.

These new insights from the H documents enrich the understanding of CL's impact, extending beyond the initial framework of Reference A. They underscore the need for a multidimensional approach to CL management, encompassing physical and mental health care, social support, and public awareness campaigns to combat stigma and improve access to care.

A crucial next step is to conduct a quantitative analysis of these new themes across a larger dataset. This would provide valuable insights into their prevalence and inform the development of targeted interventions and policies that effectively address the multifaceted challenges posed by CL.

For instance, understanding the prevalence of physical symptoms and the specific psychiatric disorders associated with CL can guide the development of specialized healthcare services. Similarly, quantifying the impact of shame and fear of stigma on treatment-seeking behavior can inform the design of community-based interventions and public awareness campaigns aimed at destigmatizing the disease and promoting early diagnosis and treatment. By integrating these quantitative findings with the qualitative insights gleaned from the H documents, a comprehensive and impactful approach to CL management can be developed, addressing the diverse needs of individuals affected by this complex disease.

After incorporating the linkages discussed above, there is one key theme emerging from the K documents that does not have a direct or indirect equivalent in Reference A:

Fear of Disease Progression and Health Consequences.
K4 explicitly mentions "fears related to physical health, including the fear of death." This fear goes beyond the social and psychological dimensions addressed in Reference A, focusing on the anxieties related to the disease's potential to cause physical deterioration, long-term health issues, and even mortality. This fear can significantly impact the individual's mental well-being and influence their healthcare-seeking behaviors.
This new theme provides a crucial addition to understanding the comprehensive impact of CL. It highlights the need to address not only the social stigma and self-perception issues but also the anxieties related to the disease's physical progression and potential health complications.
Therefore, to fully capture the nuances presented in the K documents, Reference A could be enhanced by incorporating this new main theme, **"Fear of Disease Progression and Health Consequences,"** with potential sub-themes like:

Fear of Physical Deterioration: Concerns about the disease causing physical changes and impacting mobility or functionality.

Fear of Long-Term Health Complications: Anxieties related to potential long-term impacts on health, such as organ damage or chronic conditions.

Fear of Mortality: The fear of the disease leading to death.

Incorporating this new theme and its sub-themes would provide a more holistic framework for understanding the multifaceted impact of CL, leading to more effective interventions and support strategies for those affected.
