# Supplementary material for: Advancing AI-driven thematic analysis in qualitative research: a comparative study of nine generative models on Cutaneous Leishmaniasis data
Source: BMC Med Inform Decis Mak. 2025 Mar 10;25:124. doi: 10.1186/s12911-025-02961-5 (PMC11895178; doi:10.1186/s12911-025-02961-5)

General introduction.

Drawing on the initial grounded theory analysis and incorporating new themes identified in documents g, h, and k, it becomes clear that cutaneous leishmaniasis (cl) is not merely an aesthetic concern. The findings highlight several additional dimensions (stigma by association, structural stigma, fears about disease progression, barriers to help-seeking) that enrich our understanding of the lived experiences of those affected and their social circles.

<u>Summary of new insights and their connection to the earlier analysis.</u>

1. Stigma by association.
    - fear of transmitting the disease to loved ones: this heightens isolation and a sense of guilt.
    - negative reactions from family and friends: being perceived as a “danger” to others intensifies feelings of loneliness.
      → these findings extend the notion of stigma beyond the individual, implicating the usual support networks (family, friends) in ways that can exacerbate isolation.
2. Structural stigma.
    - insufficient healthcare provision: dissatisfaction with the limited specialised resources, inadequate public information, and high costs of treatment.
    - lack of public awareness and education: a shortage of robust campaigns leads to the perpetuation of myths, fear, and discriminatory attitudes.
    - perceived government neglect: the authorities are thought to pay insufficient attention to cl, seeing it as “non-priority”.
      → in addition to interpersonal stigma, there is an institutional or systemic layer of marginalisation that increases vulnerability.
3. Gendered experiences of stigma.
    - fear of rejection in marriage: profound anxiety among women regarding physical appearance and social norms, particularly relating to visible scars.
    - heightened pressure regarding beauty standards: prominent scarring can signify “non-conformity” with cultural expectations, whereas men tend to face comparatively fewer aesthetic demands.
      → while earlier data already highlighted gender influences, the newer insights underscore how scarring can intensify psychological and social consequences, especially for young women.
4. Coping strategies and resilience.
    - minimisation and normalisation: some view cl as a common occurrence in order to preserve a sense of normality.
    - religious faith and spirituality: prayer and acceptance of divine will offer emotional support.
    - concealment and camouflage: makeup, creams, clothing, or potential surgical options are used to hide scars, reflecting the pressure to meet societal beauty standards.
      → these strategies, noted in the initial data, now appear further nuanced in explaining the deeper motives (avoiding exclusion, safeguarding self-image).
5. Physical dimensions and disease progression.
    - pain and discomfort: cl involves physical suffering that goes beyond mere appearance.
    - fear of long-term deterioration: concern about potential complications and, as noted in k4, even the prospect of death.

→ this widens the scope of analysis: cl is not solely an aesthetic stigma but also poses risks of chronic suffering and uncertainties regarding long-term health.

6. Barriers to help-seeking.
    - shame impeding consultation: a reluctance to make the disease public for fear of exacerbating stigma leads to delays in medical attention.
    - fear of stigmatisation upon diagnosis: this creates further isolation and postpones treatment.
    - lack of information on treatment options: lack of awareness heightens distress and fosters mistrust of the healthcare system.

    → such obstacles illustrate a multifaceted stigma – social, personal, and institutional.

Proposal for an original conceptual framework: the "fractal circle of vulnerabilities".

To capture the interplay of individual, social, and structural factors, we propose a conceptual framework referred to as the "fractal circle of vulnerabilities." This metaphor draws on the idea of multiple layers or levels of influence that intertwine and recur in patterns, mutually reinforcing one another.

1. Personal core: self-perception.
    - includes body image, psychological distress, and fears related to cl progression.
    - encompasses beliefs, faith, and coping mechanisms (minimisation, camouflage, etc.).
    - the initial "fracture" often arises from shame, sparking identity-based vulnerability (erikson, 1968).
2. Relational circle: stigma by association and family dynamics.
    - interactions with one's immediate circle, where the fear of infection or harm to others intensifies anxiety.
    - pressure from close contacts (for instance, partners, friends) that can either offer support or reinforce stigma.
    - a "fractal" replication of shame, since each individual in the circle may share or amplify the fear (goffman, 1963).
3. Socio-cultural sphere: gender norms and aesthetic imperatives.
    - issues surrounding marriage, femininity, masculinity, and the importance placed on beauty in social validation.
    - women and girls are especially vulnerable to stigma relating to scarring or lesions.
    - symbolic dimension: scars may be seen as signs of "impurity" (douglas, 1966).
4. Institutional and policy context: structural stigma.
    - deficiencies in public policies, limited awareness campaigns, and restricted healthcare access in some regions.
    - high costs or unavailability of treatments, with individuals feeling abandoned by the authorities.
    - weak or underdeveloped public health frameworks, leaving the population largely unsupported.
5. Fractal continuity and resilience.
    - each layer can further influence or magnify vulnerability, creating a reverberating effect.

- conversely, resources and support (for example, awareness programmes, community education, psychological assistance) can break or reduce one or more "loops" in the fractal circle.
- resilience thus emerges from the combined force of individual (faith, normalisation), family (close support), socio-cultural (community solidarity), and structural (strengthened health services) mechanisms.

Discussion and implications.
The "fractal circle of vulnerabilities" underscores how stigma operates on multiple levels: individuals feel judged by those around them, while the community is shaped by political structures, public health measures, and collective beliefs. The new themes (stigma by association, structural stigma, fear of serious disease progression, and so forth) build on and deepen our perspective, revealing the multi-faceted roots of distress linked to cl.

From a practical standpoint, the fractal approach calls for simultaneous action across different levels:

- psychological support and health education (personal core);
- mobilisation of families to reduce isolation (relational circle);
- social awareness, particularly regarding gender-based issues (socio-cultural sphere);
- strengthened public policies and improved healthcare accessibility (institutional framework).

Conclusion.
Triangulating the results demonstrates that cl is a complex phenomenon with psychological, social, structural, and physical dimensions. The "fractal circle of vulnerabilities" proposes a dynamic model that illustrates how each level of stigma and fear can ripple outward and intensify others. By integrating new themes (stigma by association, structural barriers, concerns about the disease's progression, etc.), this framework highlights the need for comprehensive strategies to alleviate distress and reinforce resilience among those affected.

Then with Napkin.ai this is the visualisation of the conceptual framework presented as Figure 2 into the manuscript.

Fractal Circle of Vulnerabilities

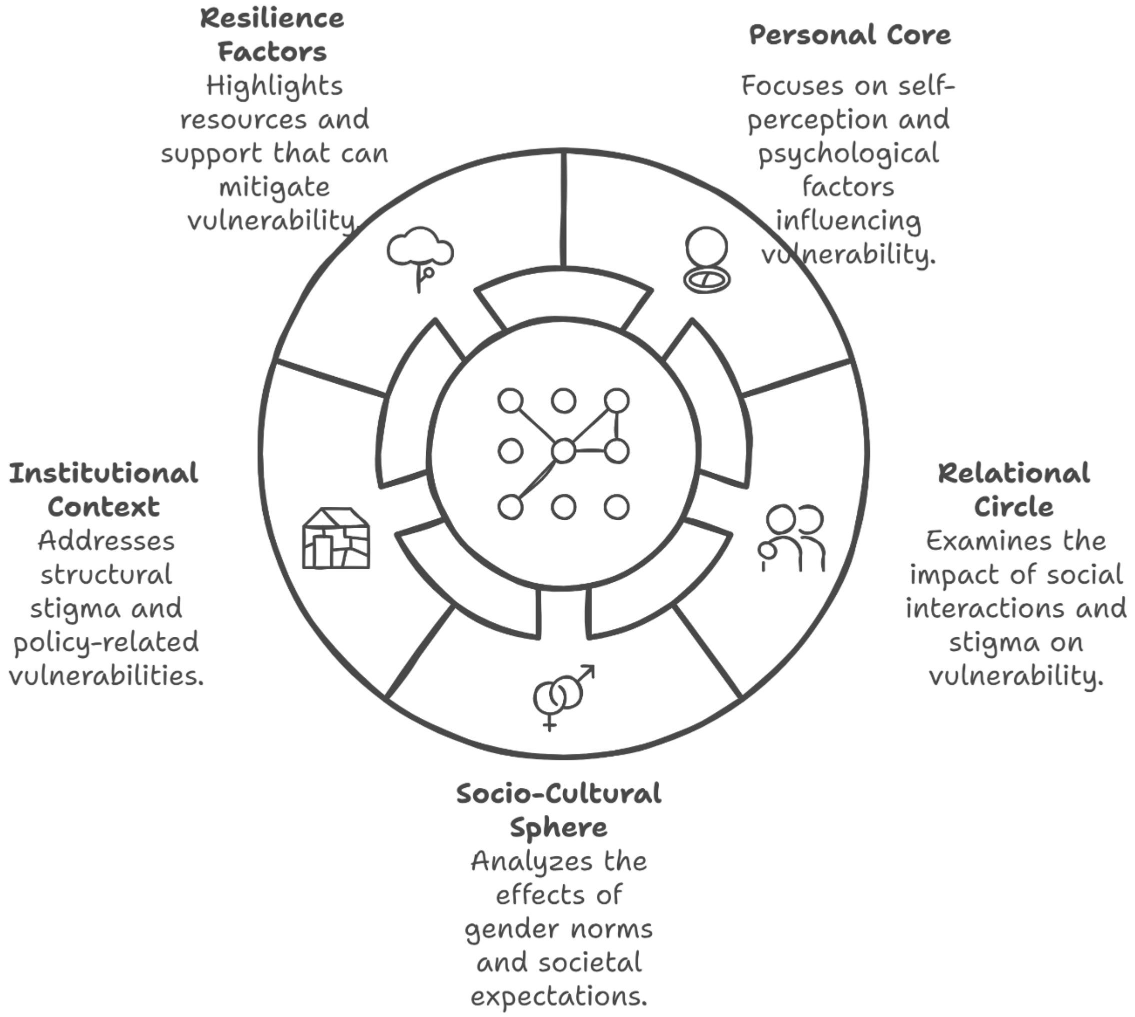

Supplement: Supplementary file 20 — Supplementary Material 20: Additional file 10qua. Phase 3C Grounded theory AI framework results [file 12911_2025_2961_MOESM20_ESM.pdf]
